# Wavelength-dependent rearrangements of an α-dione chromophore: a chemical pearl in a bis(hypersilyl) oyster†‡

**DOI:** 10.1039/d4sc00064a

**Published:** 2024-02-15

**Authors:** Gabriel Glotz, Manfred Drusgala, Florian Hamm, Roland C. Fischer, Nađa Došlić, Anne-Marie Kelterer, Georg Gescheidt, Michael Haas

**Affiliations:** a Institute of Inorganic Chemistry, Graz University of Technology Stremayrgasse 9/IV 8010 Graz Austria michael.haas@tugraz.at; b Institute of Physical and Theoretical Chemistry, Graz University of Technology Stremayrgasse 9/II 8010 Graz Austria; c Department of Physical Chemistry, Ruđer Bošković Institute Bijenička 54 Zagreb Croatia

## Abstract

The symmetric bissilyl-dione 3 reveals two well-separated n → π* absorption bands at *λ*_max_ = 637 nm (*ε* = 140 mol^−1^ dm^3^ cm^−1^) and 317 nm (*ε* = 2460 mol^−1^ dm^3^ cm^−1^). Whereas excitation of 3 at *λ* = 360/365 nm affords an isolable siloxyketene 4 in excellent yields, irradiation at *λ* = 590/630 nm leads to the stereo-selective and quantitative formation of the siloxyrane 5. These remarkable wavelength-dependent rearrangements are based on the electronic and steric properties provided by the hypersilyl groups. While the siloxyketene 4 is formed *via* a hitherto unknown 1,3-hypersilyl migration *via* the population of a second excited singlet state (S_2_, *λ*_max_ = 317 nm, a rare case of anti-Kasha reactivity), the siloxyrane 5 emerges from the first excited triplet state (T_1_*via* S_1_*λ*_max_ = 637 nm). These distinct reaction pathways can be traced back to specific energy differences between the S_2_, S_1_ and T_1_, an electronic consequence of the bissilyl substited α-dione (the “pearl”). The hypersilyl groups act as protective ‘‘oyster shell”, which are responsible for the clean formation of 4 and 5 basically omitting side products. We describe novel synthetic pathways to achieve hypersilyl substitution (3) and report an in-depth investigation of the photorearrangements of 3 using UV/vis, *in situ* IR, NMR spectroscopy and theoretical calculations.

## Abstract 2

Herein the authors report on the synthesis and photochemistry of a symmetric bissilyl-dione. This compound reveals a wavelength dependent and moreover highly selective photochemistry, which is based on the bissilyl substitution of the dione.

## Graphical abstract

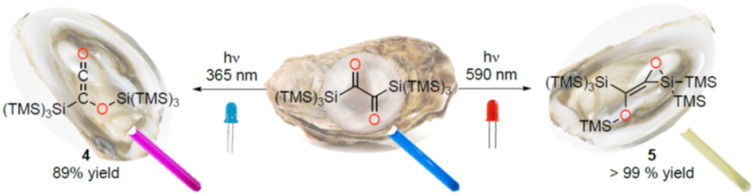

## Introduction

**Scheme 1:**
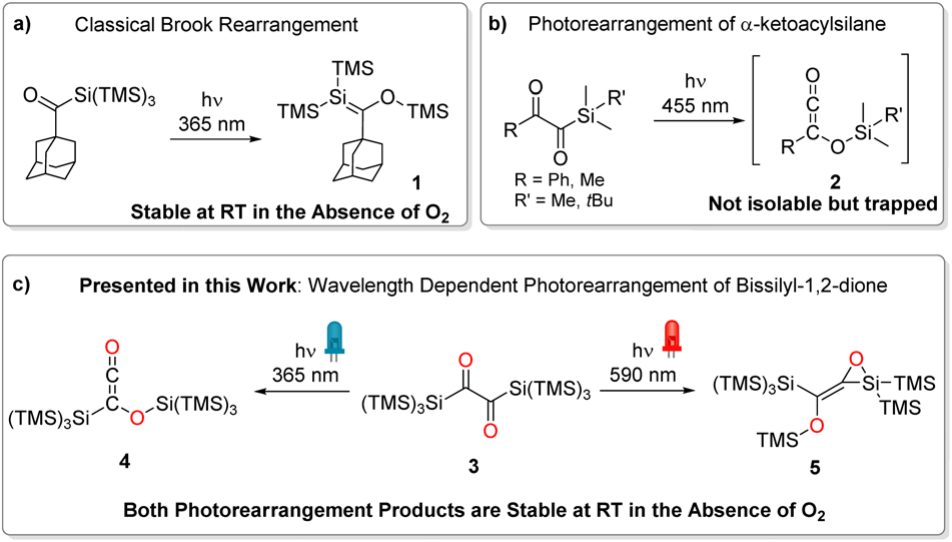
Different types of photorearrangements of acylsilanes. (a) Classical photo-Brook rearrangement, (b) photorearrangement of an α-ketoacylsilane, (c) wavelength dependent photorearrangement of 3 presented in this work.

α-Diones exhibit a wide range of photo-induced reactions that depend on various factors such as substituent character, geometry (including conformation, strain, and steric shielding), and electron distribution around the –C(O)–C(O)– moiety.^1^ As a result, butane-2,3-dione (biacetyl) reveals a distinctly different absorption spectrum than the strained bicylco[2.2.2]octene-2,3-dione^2^ or 1,2-diphenyl-ethane-1,2-dione (benzil),^3^ which again differs from 1,2-bis(2,4,6-trimethylphenyl)ethane-1,2-dione.^1,2,4,5^ Moreover, the through-bond interactions between the non-bonding orbitals of the carbonyl atoms result in the formation of two molecular orbitals with different orbital energies. Consequently, the absorption spectra of α-diones reveal two separated n → π* bands, one of which strongly overlaps with π → π* bands. Accordingly, this impairs selectively addressing such transitions and investigating wavelength-dependent photochemistry. It has been shown that silyl substituents at carbonyl moieties induce intriguing chemistry. Acylsilanes have evolved as widely used reagents in synthetic chemistry and as important intermediates in material science.^6^ Their photochemical reactivity has been extensively investigated, with roots dating back to the late 1970s.^7^ While simple acylsilanes undergo a photo-induced 1,2-silyl migration (photo-Brook rearrangement) forming siloxycarbenes,^8^ branched acylpolysilanes form metastable silenes *via* 1,3-silyl migration (compound 1, Scheme 1a).^9^ In contrast, α-diketones carrying one silane substituent yielding highly reactive siloxyketenes (compound 2, Scheme 1b) *via* scission of the Si–C bond followed by 1,3-silyl migration of the entire silyl group.^10^ An isolation of these molecules by a photochemical approach was not possible so far. However, Glorius and coworkers used various trapping agents (methanol, piperidine, and 4-toluenethiol) to underpin the formation siloxyketenes.^11^

Here we report on a novel synthetic pathway toward symmetrically bissilyl substituted α-dione exhibiting unique photorearrangements (Scheme 1c).

## Synthesis

**Scheme 2:**
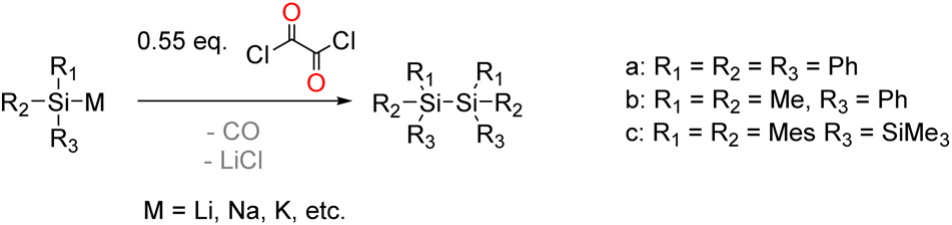
Synthesis of various silyl anions and their reaction with oxalyl chloride.

The starting point of our investigation was the synthesis of bissilyl substituted α-diones. To that end, several silyl anions were prepared and reacted with oxalyl chloride under various conditions. However, instead of the desired molecules, CO evolution together with formation of Si–Si bonds (see Scheme 2) was observed in all cases. Transmetalation to *e.g.* copper or zinc did not influence the reaction outcome (see ESI‡).

**Scheme 3:**
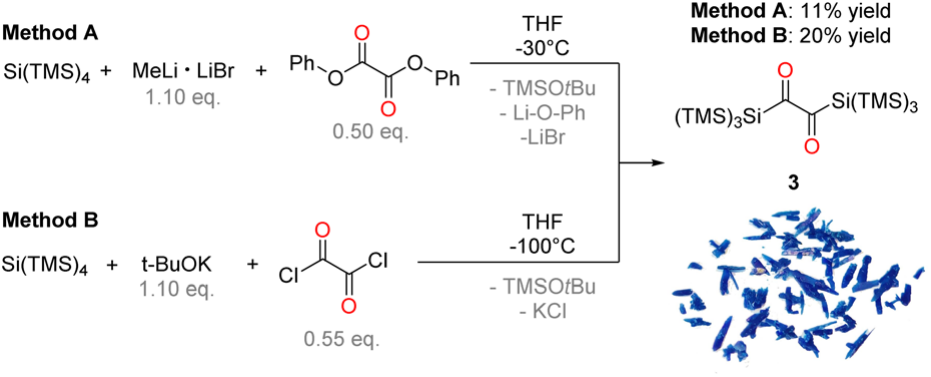
Synthesis of compound 3 by two different reaction approaches with a photo of crystals of 3.

**Fig. 1:**
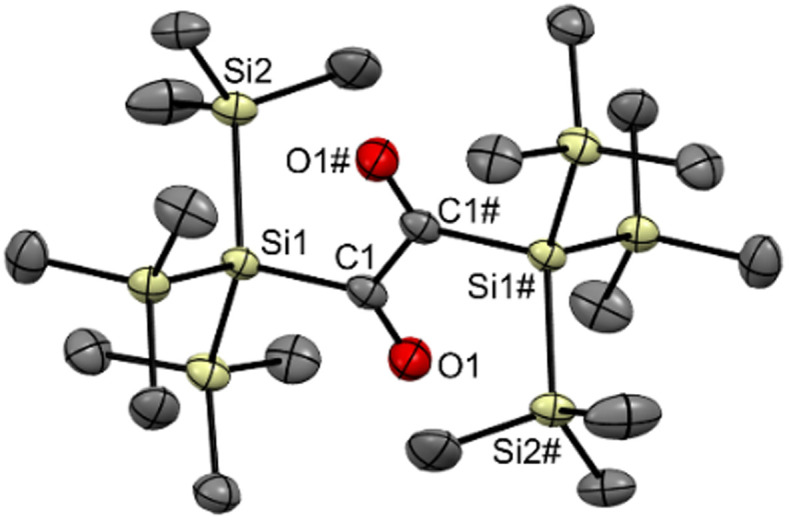
ORTEP representation for compound 3. Thermal ellipsoids are depicted at the 50% probability level. Hydrogen atoms are omitted for clarity. Selected bond lengths (Å) and bond angles (deg.) with estimated standard deviations: C1–C1# 1.539 (8), C1–O1 1.237 (5), Si1–C1 1.924 (4), Si1–Si2 2.355 (2), O1–C1–C1#–O1# 180.00.

Markedly, using alkali metal-substituted tris(trimethylsilyl)-silanides^12,13^ either with oxalyl chloride or diphenyl oxalate (methods A and B, Scheme 3, resp.) afforded bissilyl-dione 3. Optimization of the reaction conditions again revealed a delicate balance between the formation of 3 and 1,1,1,4,4,4-hexamethyl-2,2,3,3-tetrakis(trimethylsilyl)tetrasilane by metal-halide exchange of the silanide.^14^ Change of the solvent or counterion for the silanide formation did not increase the yield of 3. However, lowering the temperature effectively attenuated the metal-halide exchange reaction thereby increased the yield of 3 (20% at −100 °C *vs.* 3% at −30 °C). Compound 3 is air-stable and can be stored at room temperature in the dark for months without the observation of degradation products. Compound 3 crystallizes in the triclinic space group *P*1̄ with 4 molecules per unit cell. The carbonyl groups adopt a *trans* orientation and are coplanar (180° dihedral angle, Fig. 1). The ^1^H, ^13^C and ^29^Si NMR spectra underpin that 3 has the same connectivity in solution (see ESI‡).

### Spectroscopy

**Fig. 2:**
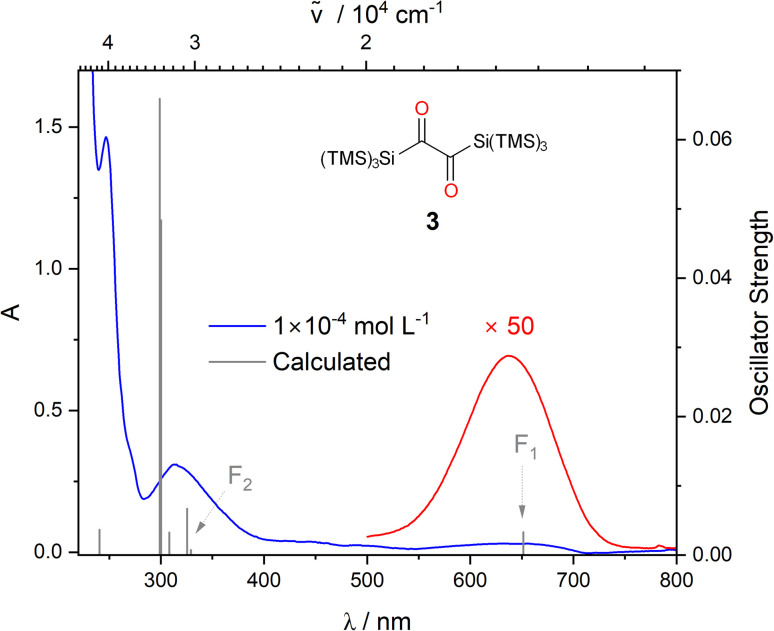
UV/vis spectra of 3, experimental spectra in DCM at two different concentrations (blue: 1 × 10^−4^ mol L^−1^, red: 5 × 10^−3^ mol L^−1^), the computed vertical transitions are given as bars. Solvent dependence of the long wavelength absorption band, and the orbital pictures for the two relevant transitions can be found in the ESI Fig. S1c and S48‡ respectively.

The UV/vis spectrum of 3 shows three characteristic bands centered at *λ* = 247, 317, and at *λ* = 637 nm (Fig. 2). TDDFT calculations reveal that the band at *λ* = 247 nm can be attributed to a π → π* transition, whereas the two rather distant bands at *λ* = 317 and at *λ* = 637 nm correspond to n → π* transitions. More precisely, calculations together with deconvolution of the experimental UV/vis spectra show that the band at 317 nm (*ε* = 2460 mol^−1^ dm^3^ cm^−1^) comprises 
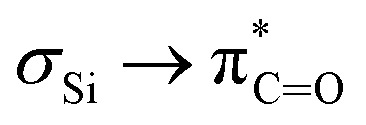
 and n → π* transitions (Fig. S1a‡ and F_2_ in Fig. 2). This spectral fingerprint, showing two n → π* bands separated from the π → π* band,^1,15^ is remarkable and is the unique basis for the photorearrangements of 3.

### Photo-induced reactions of 3

Many α-diones undergo α-cleavage to form acyl-type radicals and initiate radical polymerizations. An ideal technique to test if 3 fragments into radicals is ^1^H CIDNP (chemically induced dynamic nuclear polarization) spectroscopy.^16–18^ When short-lived radicals are formed, CIDNP spectra taken in the presence of butyl acrylate show polarized NMR signals (enhanced emission/absorption) and the formation of aldehydes. Performing such experiment with a 10 fold excess of butyl acrylate (*λ* = 355 nm, 3rd harmonic Nd:YAG laser), no polarized signals and no aldehydes were detected – essentially ruling out photo-induced homolytic cleavage of 3 (Fig. S2‡).

**Fig. 3:**
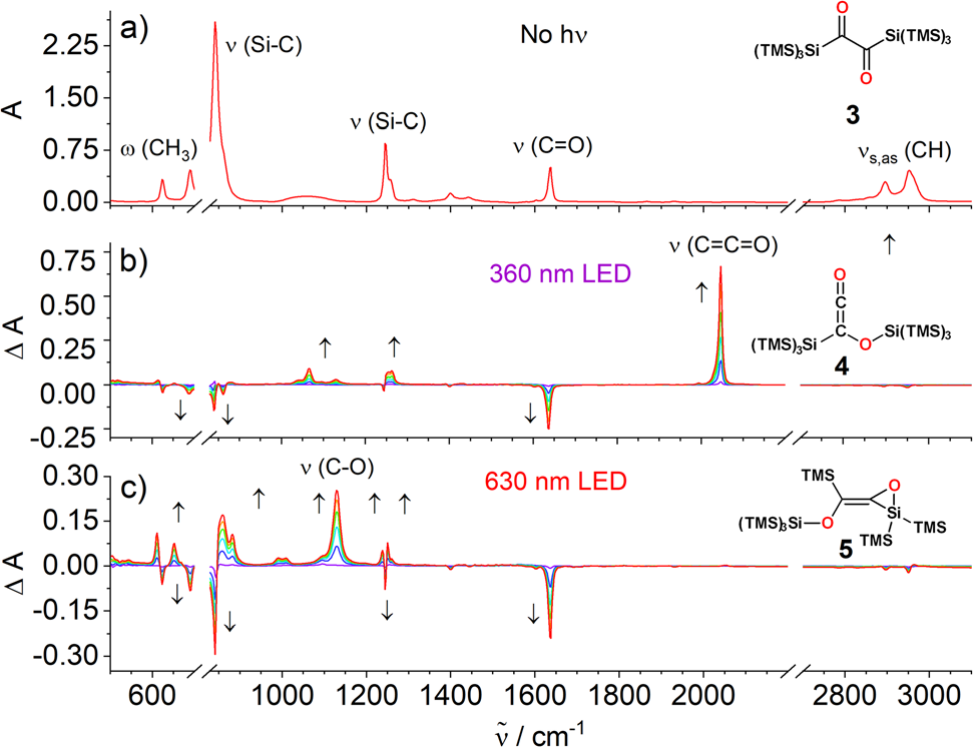
(a) Experimental IR spectra of 3 in CCl_4_. The 2200–2700 cm^−1^ region omitted for clarity. Time-resolved difference IR spectra during irradiation of 3 with (b) a LED having the emission maximum at 360 nm, and (c) LED having emission maximum at 630 nm. The solvent signals are subtracted from the spectra and the 700–828 cm^−1^ region is cut out because it is dominated by solvent absorption.

Owing to the two well-distinguishable n → π* absorption bands of 3 at *λ* = 317 and at *λ* = 637 nm (Fig. 2), we have used LEDs with emission maxima at *λ* = 360 and 365 nm (≈330 kJ mol^−1^) and at *λ* = 590 and 636 nm (≈190 kJ mol^−1^) to selectively and separately address the two distinct n → π* transitions (Fig. S4 and S14‡ for emission spectra of the respective LEDs). Infrared spectroscopy (IR) is the ideal method to monitor the conversions of carbonyl groups. It is highly sensitive for detecting changes at carbonyl groups because the vibrational frequencies of C=O groups are well distinguishable and characteristically change upon their conversions (^1^H NMR is hardly suitable here because the methyl hydrogens are too remote from the carbonyl groups).^19^ The IR spectrum of 3 in CCl_4_ at room temperature shows bands (Fig. 3a) at 2950 cm^−1^ and 2895 cm^−1^ (asymmetric and symmetric C–H stretching vibrations, respectively, *ν*_as,s C–H_), 1635 cm^−1^ (asymmetric C=O stretching vibration, *ν*_C=O_), and bands from Si–C stretching (*ν*_Si–C_) and methyl group wagging vibrations (*ω*_CH_3__, Fig. S3‡).

**Scheme 4:**
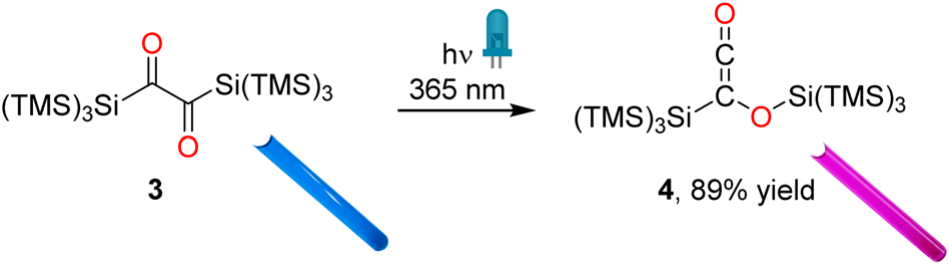
Photorearrangement of 3 to 4 using the LEDs with emission maxima centered at 365 nm. Photos of the corresponding NMR tubes are presented as insets.

**Fig. 4:**
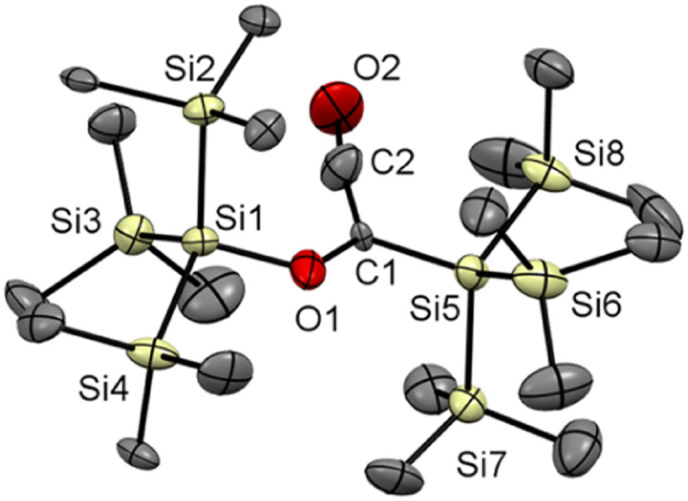
ORTEP representation for compound 4. Thermal ellipsoids are depicted at the 50% probability level. Hydrogen atoms are omitted for clarity. Selected bond lengths (Å) with estimated standard deviations: Si1–O1 1.77 (2), C1–O1 1.313 (15), Si5–C1 1.816 (19), C1–C2 1.379 (19), C2–O2 1.188 (12).

Irradiation of 3 at 360 nm causes a decrease of the C=O band (1635 cm^−1^) that correlates with the simultaneous appearance of a band at 2046 cm^−1^ (*ν*_C=C=O_), which we assign to the ketene group of siloxyketene 4 (Fig. 3b and S5‡).^20^ This comes along with intensity changes of Si–C vibration modes and a new band at 1065 cm^−1^ attributed to the C–O stretching vibration of 4. Analogous experiments at a preparative scale with 3 in C_6_D_6_ in a photoreactor using *λ* = 365 nm high-power LEDs revealed a highly selective reaction (^1^H NMR monitoring) with only minor amount of side products (Scheme 4 and Fig. S6‡). We were able to isolate 4 and obtain its crystal structure (Fig. 4) after dissolving the crude reaction mixture in *n*-pentane and cooling to −70 °C, which resulted in the precipitation of pink crystals in excellent yields of 89%.

The spectroscopic data are consistent with 4 (Fig. S27–S29‡), with one resonance in the ^29^Si NMR spectrum for the silicon atom bearing the ketene moiety at *δ* = −77.0 ppm, two signals at *δ* = −15.8 ppm and −11.8 for the six SiMe_3_ groups and one significantly downfield shifted signal at *δ* = 19.0 ppm for the silicon atom covalently bound to oxygen. Additionally, the ^13^C NMR spectrum revealed the characteristic shift at *δ* = 219.0 ppm for the ketene carbonyl group. Compound 4 can be stored under inert conditions for months at room temperature without detecting degradation products. The enhanced persistence can be traced back to the sterically demanding hypersilyl groups. Interestingly, Scheschkewitz and coworkers employed group 6 metal carbonyls as stabilizing entity to isolate a silylketene.^21^

Addressing the band at 637 nm with a *λ* = 630 nm LED produced distinctly different time-resolved difference IR spectra than irradiation at *λ* = 360 nm (Fig. 3c). Markedly, a band emerges at 1130 cm^−1^, attributable to the C–O bond stretching of 5 (*ν*_C–O_) with the C=O vibration band at 1630 cm^−1^ decreasing. Additionally, several new bands in the 850–950 cm^−1^ region appear indicating changes in the Si–C stretching vibrations together with several bands in the 500–650 cm^−1^ region, characteristic for Si–C and methyl group wagging vibrations (Fig. S15‡).

**Fig. 5:**
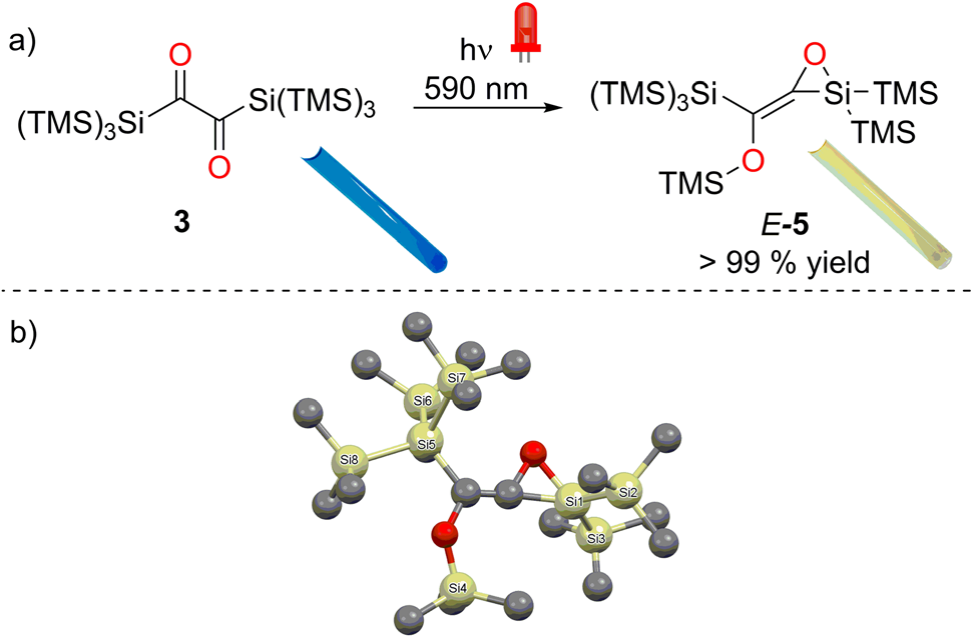
(a) Photorearrangement of 3 to 5 using the LED with emission maxima centered at *λ* = 590 nm, photos of NMR tubes for 3 and 5 are presented as inserts. (b) Structural Confirmation by X-ray analysis.

A preparative experiment with 3 in C_6_D_6_ using a photoreactor equipped with high-power *λ* = 590 nm LEDs revealed a highly selective reaction (Fig. 5a, ^1^H NMR spectra for the product formation 5 during the irradiation, Fig. S16‡). We were able to isolate product 5 as a yellowish oil (the melting point of the compound is around room temperature) with excellent yield (>99%, Fig. 5a). Crystallization from *n*-pentane at −70 °C afforded crystals, revealing the structure for 5 (in moderate quality, Fig. 5b). Despite the possibility of two configurational isomers of 5, the *trans* arrangement of the carbonyl groups found in 3, leads to the stereoselective formation of *E*-5. This is also in accordance with the photochemical mechanism (Fig. 9). The ^29^Si NMR spectrum showed five resonances. On the basis of previous experience with structurally related compounds, we were able to assign all signals (Fig. S30–S32‡).^22^

### Mechanistic considerations

**Scheme 5:**
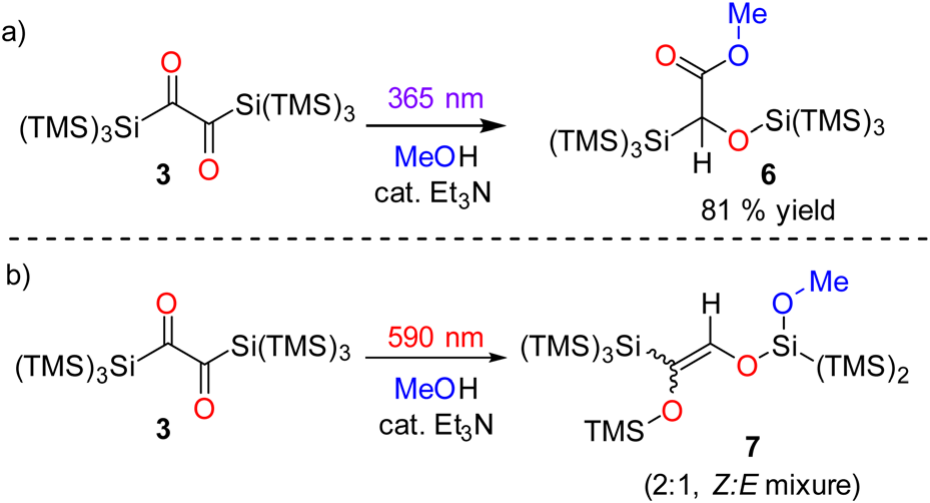
Methanol trapping of products during the photore-arrangement of 3 (a) at *λ* = 365 nm, and (b) at *λ* = 590 nm.

**Fig. 6:**
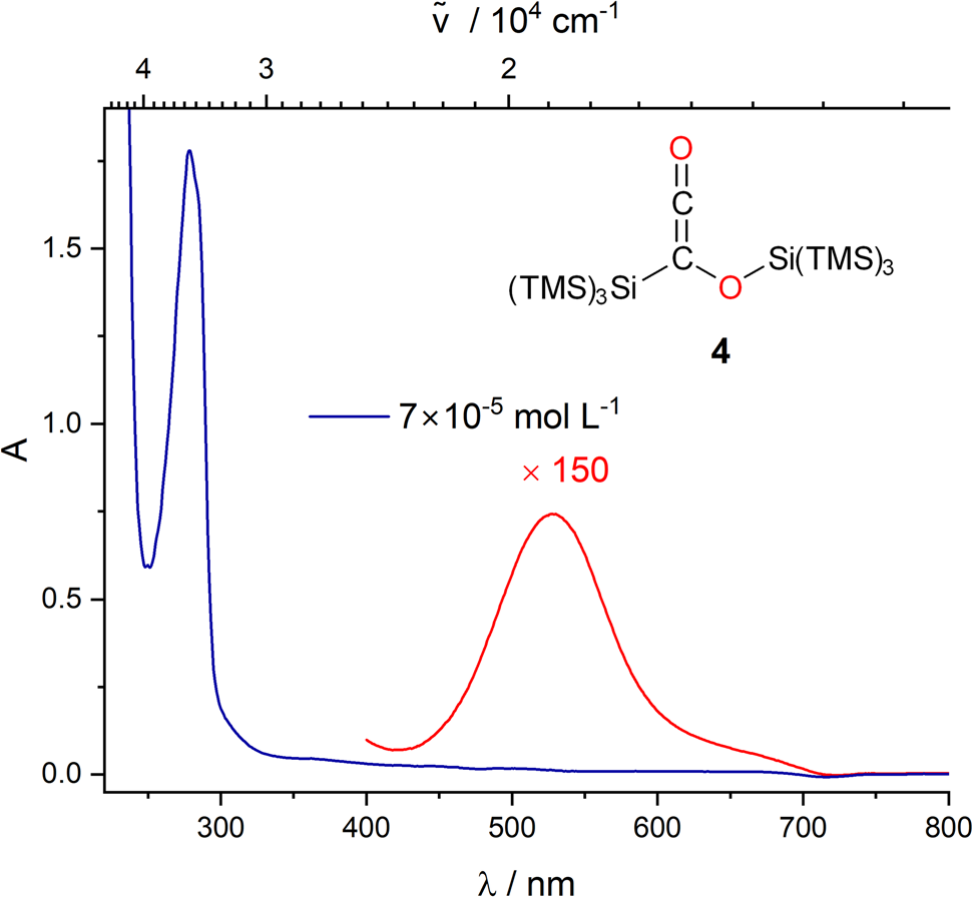
UV/vis spectra of 4 in THF at two different concentrations (blue: 7 × 10^−5^ mol L^−1^, red: 1 × 10^−2^ mol L^−1^).

We did not observe any emission upon excitation of 3 at 77 K or 293 K with 360 nm and 630 nm. This shows that radiative transitions do not compete with photorearrangements as relaxation pathway of excited states. To exclude the formation of siloxycarbenes, we performed irradiation experiments of 3 in an excess of dry methanol with catalytic amounts of triethylamine as base at both wavelengths (*λ* = 365 and 590 nm). The irradiation at *λ* = 365 nm showed a color change from blue to purple pointing towards the formation of 4. This was confirmed by NMR spectroscopy. Additionally, a new set of signals was observed, indicating that 4 slowly reacts with methanol forming a new product. The reaction was finished after 7 days, as indicated by NMR spectroscopy. We assume that the reason for this long reaction time is steric hindrance by the hypersilyl groups, shielding the ketene unit. The crude product was recrystallized from *n*-pentane to separate product 6 from small amounts of an uncharacterized side product (Scheme 5a). The spectroscopic data of 6 are consistent with the proposed structure (see Fig. S33–S35‡). Moreover, the UV/vis spectrum of 4 shows well-defined absorption maxima centered at *λ* = 279 nm and *λ* = 528 (*ε* = 82 mol^−1^ dm^3^ cm^−1^) nm (Fig. 6 and S21‡). The longest-wavelength absorption band of 4 overlaps with that of 3 (*λ* = 637 nm absorption, Fig. S22‡), thus 4 could potentially serve as an intermediate for the formation of 5. To test this hypothesis, we irradiated a solution of 4 in C_6_D_6_ with *λ* = 550 nm, 590 nm, and 630 nm LEDs (Fig. S23‡), but we could not detect any conversion neither by IR nor by NMR spectroscopy. Furthermore, we performed a methanol trapping experiment of 5 with catalytic amounts of triethylamine by irradiating the solution of 3 containing methanol and base at *λ* = 590 nm. While monitoring the conversion of 3 by NMR-spectroscopy, the selective formation of two products, as a *Z*/*E* isomeric mixture (2 : 1 ratio), *via in situ* ring opening of 5 was observed. Isolation of the main isomer as colorless oil was performed by preparative thin-layer chromatography. Analytical data corresponds well with the proposed structural arrangement. Additional evidence about the structural conformation of 7 was given by an HSQC NMR measurement, also confirming the direct bonding between the hydrogen atom and the carbon atom of the C=C bond (Scheme 5b, for further details, see Fig. S36–S42‡).

### Photochemical pathway from 3 → 4

**Fig. 7:**
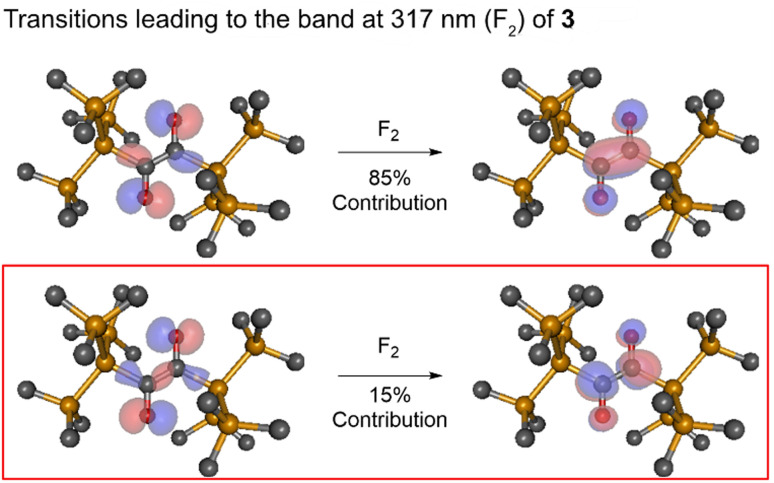
Dominant NTO pairs contributing to the S_2_ transitions of 3 computed at the Franck–Condon geometry. The second singlet vertical transition F_2_ (*cf.*Fig. 2) has two contributions of 
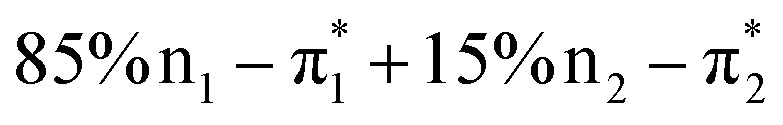
 (the latter being decisive for the rearrangement). Here n_1_ and n_2_ are the asymmetric and the symmetric linear combination (LCAO) of the two n orbitals, respectively, while 
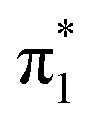
 is the positive and 
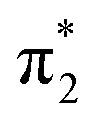
 is the negative LCAO of the carbonyl π* orbitals. The computations were performed with ADC(2)/cc-pVDZ.

**Fig. 8:**
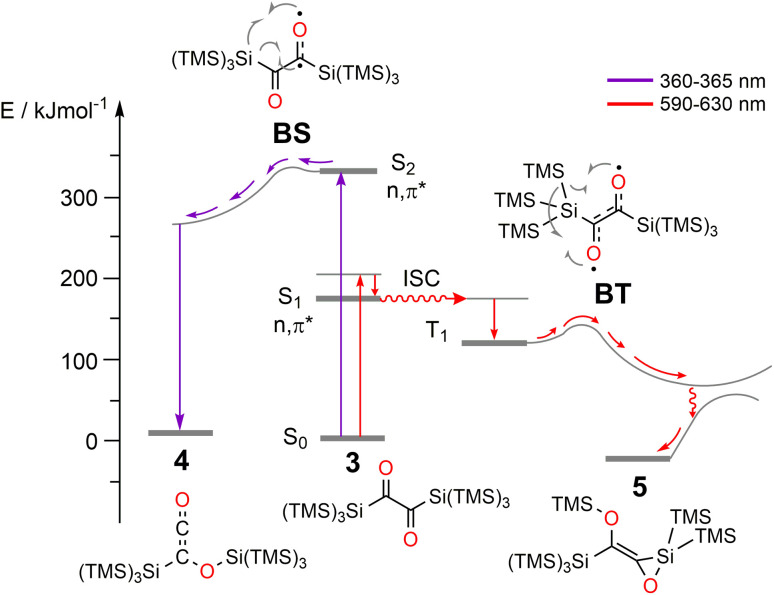
Schematic representation of Jablonski energy diagram describing wavelength dependent photorearrangements of 3. The energies are referenced to S_0_ state of 3.

To test whether the 3 → 4 process proceeds *via* a triplet or singlet pathway, we have explored if the reaction is quenched by the presence of oxygen. Experimentally, there was no oxygen influence on the reaction rate or outcome (Fig. S8‡), indicating that the reaction proceeds *via* a singlet state. Furthermore, a very low activation energy, *E*_a_ = 0.63 ± 0.35 kJ mol^−1^ (in the 292–188 K range, Fig. S9–S13‡) and first-order kinetics underpin this assumption. The experimental irradiation wavelengths of 360/365 nm, provide an excitation energy of *ca.* 330 kJ mol^−1^ (Fig. S7‡), necessary to induce the n → π* transition being a component of the band centered at *ca.* 317 nm (see Fig. S1‡). ADC(2) calculations show that this energy is sufficient to populate the second excited singlet state, S_2_ (n,π*) of 3 (calc. *E*_rel_ = 286 kJ mol^−1^). The corresponding natural transition orbitals (NTOs) are presented in Fig. 7 (see the ESI‡ for details). The energy gap between the first excited singlet state, S_1_ and S_2_ is substantial (≈167 kJ mol^−1^, ≈14 000 cm^−1^), in line with attenuated (inefficient) S_2_ → S_1_ internal conversion. This is consistent with the observation that basically no 5 is formed (*via* S_1_ → T_1_, *vide infra*). This phenomenon that the photo reaction proceeds from the higher excited state (S_*n*,_ where *n* > 1) is a rare example of anti-Kasha reactivity.^23^ The low activation barrier observed may also point to a conical intersection of the S_2_ state of 3 with the ground state towards 4. The S_2_ transition for this rearrangement is that from the n to the π* system with the decisive transition (with 15% contribution, depicted in the lower trace of Fig. 7) from the HOMO to the LUMO+1, or in other words from the symmetric linear combination of the n orbitals to the antisymmetric linear combination of the π* system at the C=O groups. Upon excitation to the S_2_ state, negative charge is translocated from the C=O to the C–C bond. Indeed, in the minimum of the S_2_ state, one of the C–O bonds is slightly elongated (C–O = 1.318 *vs.* 1.309 Å), and the central C–C bond is shortened by 0.127 Å compared with the ground state. This translates into a singlet-1,2-biradicaloid character (n,π*) at the C=O groups in S_2_ (Fig. S47‡ for bond lengths) allowing 1,3-hypersilyl group migration to the distal carbonyl oxygen (BS in Fig. 8, left side of the diagram) yielding 4. A classical Brook rearrangement would only yield a 1,3 migration of one trimethylsilyl group.

### Photochemical pathway from 3 → 5

In contrast to the above findings for 3 → 4, the conversion 3 → 5 at *ca.* 630 nm is quenched in the presence of oxygen pointing to the involvement of a triplet-state. This is substantiated by the doubling of the rate for the 3 → 5 reaction (*k*_3__→__5_ increase from 0.108 to 0.209 min^−1^) after adding CHBr_3_ to the solution – a clear demonstration of an external heavy atom effect on intersystem crossing (ISC) (Fig. S18‡). In addition, we have used a series of quenchers with various triplet state energies to delimit the T_1_ energy of 3 being *ca.* 115 kJ mol^−1^ (see ESI‡).^24^ The calculated ADC(2) energy of S_1_ state (n,π* character) is 167 kJ mol^−1^ and, the first excited triplet state (T_1_) has an energy of 140 kJ mol^−1^ (for details see the ESI‡). Accordingly, irradiation at *λ* = 590/630 nm (≈190 kJ mol^−1^) is sufficient to populate the S_1_ state, which then undergoes ISC to the triplet state T_1_ (Fig. S50‡). The latter represents a 1,4-triplet biradical (BT in Fig. 8), undergoing 1,4-trimethylsilyl migration, affording product *E*-5 with an activation barrier of *ca.* 27 kJ mol^−1^ (exp. based on 2 × *ν*_C–O_ ≈ 2260 cm^−1^, see Fig. S46–S49‡ for further details: calc. 29 kJ mol^−1^, DFT). The wavelength-dependent rearrangements are summarized on Fig. 8.

### General considerations on the photoreactivity of substituted α-diones

The aim of this section is to shed light on the electronic properties of the hypersilyl group, Si(SiMe_3_)_3_ with regard to an appropriate ordering of the electronic states and to specific shapes of the molecular orbitals, to cause the unexpected photo-reactivity described above.

**Fig. 9:**
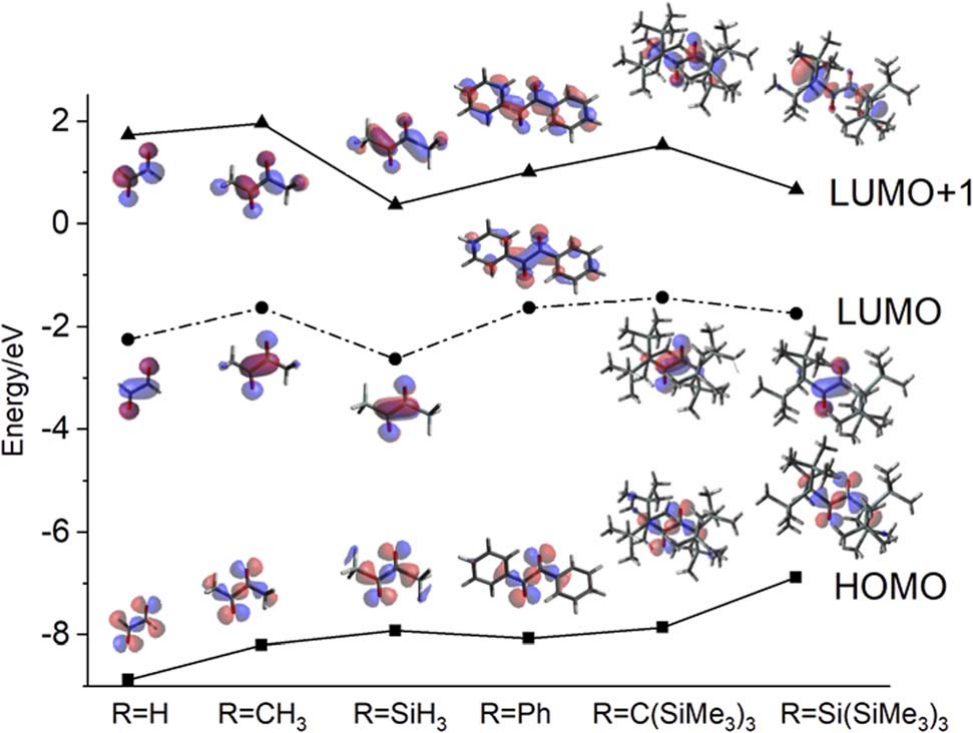
Energy diagram of HOMOs, LUMOs, and LUMOs+1 of selected symmetric α-diones.

The most remarkable observation is the “anti-Kasha” reactivity leading to the formation of 4. To this end, we will concentrate on the discussion of the HOMOs and the LUMOs+1 of exemplary α-diones. Glyoxal and biacetyl (Fig. 9, R = H, CH_3_, respectively) are of importance in atmospheric chemistry and their electronic structures have been reported.^25^ Whereas their HOMOs are of n-type for all derivatives, the LUMOs are of π* character with symmetric linear combination of the two C=O groups, changing to asymmetric linear combination of π* for the LUMOs+1. Replacing the methyl group of biacetyl by a phenyl group yields benzil, a popular type II photoinitiator. The phenyl ring causes a decrease of the S_2_ state energy as phenyl orbitals shift in creating a low-energy π*-type LUMO+1. The relevant π* orbital with the same symmetry as the LUMO in biacetyl becomes a higher virtual orbital but with the same energy in the series (see Fig. 9). The tris(trimethylsilyl)-methyl (R=C(SiMe_3_)_3_) derivative indicates orbital characters compatible with biacetyl (glyoxal) with the HOMO energy being shifted to a slightly less negative value and the LUMO+1 being somehow stabilized. Interestingly, silicon substitution leads to a significant stabilization of the LUMO and LUMO+1. Introduction of the sterically more demanding hypersilyl moiety (R=Si(SiMe_3_)_3_) destabilizes the HOMO even further, in line with the hypersilyl substituent being a clearly electron-donating substituent.^26^ At the same time, the LUMO+1 becomes stabilized *vs.* the carbon derivative diminishing the energy required to populate a corresponding state. Furthermore, the shape of the LUMO+1 indicates an increased electron density at the central π-dione moiety and at the Si atoms of the trimethylsilyl groups rationalizing the reaction depicted in Scheme 4 (see also Fig. 9). In addition, the bulkiness of the hypersilyl groups (Fig. 4) impairs the rotation around central C(O)–C(O) bond, effectively modulating the photoactivity of the 1,2-dione. Conformation-reactivity relationships of several 1,2-diones can be found in the respective literature.^1,27,28^ Furthermore, hypersilyl groups protect the central chromophore from bimolecular side reactions. The formation of 4 demonstrates that it is not only the electron donating and steric effect of the hypersilyl groups enabling this reactivity but also the specific impact on the energy of the LUMO+1, which enables the observation of the wavelength-dependent rearrangements of 3 to 4 and 5, like pearls in a protecting oyster shell.

## Conclusions

We have established a synthetic pathway towards the novel bishypersilyl-1,2-dione 3*via* coupling of alkali metal-substituted tris(trimethylsilyl)-silanides with oxalyl chloride or diphenyl oxalate. This molecule exhibits unique photo conversions, which are wavelength dependent. The symmetric hypersilyl substitution of the α-dione chromophore causes an exceptional ordering of orbitals leading to the presence of S_1_ and S_2_ states of n,π* character, connected with characteristic absorption bands centered at *λ*_max_ = 637 and *λ*_max_ = 317 nm, respectively. Selective irradiation of either of these bands yields siloxirane 5 or siloxyketene 4, respectively. The mechanism of the wavelength dependent photorearrangements are summarized in Fig. 8. This reactivity is caused by two principal factors: (i) the electronic effect of the hypersilyl groups (on the HOMO, LUMO and the LUMO+1) and, (ii), the bulkiness of the Si(SiMe_3_)_3_ substituents, which impair rotation around central C(O)–C(O) bond and bimolecular reactions thus promoting intramolecular rearrangements. On one hand, the electron distribution in the LUMO+1 rationalizes the formation of 4, on the other hand, the biradicaloid character at the oxygens of the triplet state of 3 explains the TMS migration toward one oxygen atom and the O–Si ring closure (Fig. 8). It is remarkable that the hypersilyl substituent causes that the small α-dione chromophore becomes an orthogonal two-wavelength responsive system with one displaying anti-Kasha reactivity. These observations indicate the scope of hypersilyl substituents for developing photoresponsive molecules with unusual properties.

## Data availability

PBEh-3c geometries of 3,4,5 and the transition states as well as the optimized MP2 and SOS-ADC(2) geometries with their respective energies as Cartesian coordinates in xyz format.

## Author contributions

G. G. and M. D. were equally responsible for experimental investigations. G. G. performed formal analysis, visualization, data presentation, and writing the original draft (lead). F. H., N. D. and A. K. were responsible for calculations. R. F. measured and analyzed the single crystal X-ray structures. G. Ge. was responsible for manuscript editing. M. H. was in charge of methodology and conceptualization, review and editing of the manuscript (lead), project administration, and funding acquisition.

## Conflicts of interest

There are no conflicts to declare.

## Supplementary Material

SC-015-D4SC00064A-s001

SC-015-D4SC00064A-s002

SC-015-D4SC00064A-s003
