# Supplementary material for: Wavelength-dependent rearrangements of an α-dione chromophore: a chemical pearl in a bis(hypersilyl) oyster
Source: Chem Sci. 2024 Feb 15;15(12):4427–33. doi: 10.1039/d4sc00064a (PMC10952070; doi:10.1039/d4sc00064a)
Supplement: SC-015-D4SC00064A-s002 [file SC-015-D4SC00064A-s002.pdf]

Electronic Supplementary Material (ESI) for Chemical Science.
This journal is © The Royal Society of Chemistry 2024

Wavelength-Dependent Rearrangements of an *α*-Dione Chromophore: A Chemical Pearl in a Bis(hypersilyl) Oyster

Gabriel Glotz,[‡][b] Manfred Drusgala,[‡][a] Florian Hamm,[b] Roland C. Fischer,[a] Nađa Došlić,[c] Anne-Marie Kelterer,[b] Georg Gescheidt*[b] and Michael Haas*[a]

[a] DI Manfred Drusgala, Prof. Roland C. Fischer and Dr. Michael Haas
Institute of Inorganic Chemistry, Graz University of Technology
Stremayrgasse 9/IV, 8010 Graz (Austria)
E-mail: michael.haas@tugraz.at

[b] Dr. Gabriel Glotz, Florian Hamm, BSc, Prof. A. Kelterer, Prof. G. Gescheidt
Institute of Physical and Theoretical Chemistry, Graz University of Technology
Stremayrgasse 9/II, 8010 Graz (Austria)
E-mail: g.gescheidt-demner@tugraz.at

[c] Dr. Nađa Došlić
Department of Physical Chemistry, Ruđer Bošković Institute,
Bijenička 54, Zagreb (Croatia)

[‡] These authors contributed equally to this work.

Abstract: The symmetric bissilyl-dione, **3** reveals two well-separated n→π* absorption bands at λ_{max} = 637 nm (ε = 140 mol^{-1} dm^3 cm^{-1}) and 317 nm (ε = 2460 mol^{-1} dm^3 cm^{-1}). Whereas excitation of 3 at *λ* = 360/365 nm affords an isolable siloxyketene **4** in excellent yields, irradiation at *λ* = 590-630 nm leads to the stereo-selective and quantitative formation of the siloxyrane **5**. These remarkable wavelength-dependent rearrangements are based on the electronic and steric properties provided by the hypersilyl groups. While the siloxyketene **4** is formed via a hitherto unknown 1,3-hypersilyl migration via the population of a second excited singlet state (S_2, λmax = 317 nm, a rare case of Anti-Kasha reactivity), the siloxyrane **5** emerges from the first excited triplet state (T_1 via S_1 λmax = 637 nm). These distinct reaction pathways can be traced back to specific energy differences between the S_2, S_1 and T_1, an electronic consequence of the bissilyl substited α-dione (the "pearl"). The hypersilyl groups act as protective ''oyster shell'', which are responsible for the clean formation of **4** and **5** basically omitting side products. We describe novel synthetic pathways to achieve hypersilyl substitution (3) and report an in-depth investigation of the photorearrangements of **3** using UV/Vis, in-situ IR, NMR spectroscopy and theoretical calculations.

Table of Contents

General Information ... 3
Synthesis ... 3
Large-scale irradiation experiments ... 3
UV/Vis Spectroscopy ... 3
FT-IR Spectroscopy ... 3
NMR and CIDNP Spectroscopy ... 3
Mass spectrometry ... 3
DFT Calculations ... 4
ADC(2) Calculations ... 4
Experimental Procedures ... 4
Synthesis of 3 with diphenyl oxalate ... 4
Synthesis of 3 with oxalyl chloride ... 4
Synthesis of 4 ... 5
Synthesis of 5 ... 5
Photolysis Experiments of 3 in the presence of MeOH at 365 nm ... 5
Photolysis Experiments of 3 in the presence of MeOH at 590 nm ... 5
Photochemical Investigations ... 7
Photochemical Investigation of 3 ... 7
Irradiation of 3 with light source having an emission center at 360 - 365 nm ... 9
Irradiation of 3 with light sources having emission maxima at 590 - 630 nm ... 14
Photochemical Investigation of 4 ... 17
Irradiation of 4 with light sources having an emission maxima 590 / 630 nm ... 18
NMR Spectroscopy ... 19
NMR spectra of 3 ... 19
NMR spectra of 4 ... 22
NMR spectra of 5 ... 25
NMR spectra of 6 ... 28
NMR spectra of 7 ... 31
X-Ray Crystallography ... 38
Crystallographic Table ... 38
Calculations ... 39
Computed IR Spectra for compound 3,4 and 5 ... 41
Computed rearrangement mechanism of 3 ... 43
Photochemical Pathway from 3→4: ... 43
References ... 46

General Information

Synthesis

All experiments were performed under a nitrogen atmosphere using standard Schlenk techniques. Solvents were dried using a column solvent purification system.[1] Me_3SiCl (≥ 99%), $SiCl_4$ (99%), KO*t*Bu (> 98%), oxalyl chloride (≥ 98%), methyllithium-lithium bromide complex solution 1.5M in diethyl ether, toluene-d_8 (99 atom%, D) and benzene-d_6 (99.5 atom%, D) were used without any further purification. Methanol was dried with magnesium and iodine according to published procedures[2]. For the measurement of air-sensitive samples, benzene-d_6 was additionally dried above a sodium/potassium alloy at 12-hour reflux. Tetrakis(trimethylsilyl)silane[3] was prepared according to published procedures.

Melting points were determined using the Stuart SMP50 apparatus and are uncorrected. Elemental analyses were carried out on a Hanau Vario Elementar EL apparatus.

Large-scale irradiation experiments

Photochemical experiments were run on a self-made photo reactor with arrays of various light-emitting diodes (LED) having an emission spectrum centered at 365 nm, 405 nm, 550 nm, and 590 nm, respectively. The photo reactor setup comprises 24 LEDs per defined wavelength. Performed experiments were run with a total electrical output power of 25W. Additionally, an integrated cooling system is keeping a constant reaction temperature of 23°C.

UV/Vis Spectroscopy

UV/Vis spectra were acquired either using a TIDAS UV/Vis spectrometer equipped with optical fibers and a 1024-pixel diode-array detector (J&M Analytik AG, Essingen, Germany), or a Perkin Elmer Lambda 5 spectrometer.

FT-IR Spectroscopy

The steady-state and time-resolved FT-IR spectra in solution were recorded on a Bruker Alpha spectrometer running OPUS 7.5 software in transmission mode. The custom-made IR cell was placed inside the spectrometer and the IR spectra were averaged over 22 scans (25 s each) in the range from 500 - 4000 cm^{-1} at a resolution of 4 cm^{-1}. If necessary, the Savitzky Golay smoothing and baseline correction were applied to the spectra. The low-power LEDs (> 50 mW) were purchased from Roithner Laser Technik Gmbh, The LED is driven in a constant current mode using Keithley 224 programmable current source, allowing for precise control and high stability of the light output. Solid samples were recorded on a Bruker Alpha-P Diamond ATR Spectrometer.

NMR and CIDNP Spectroscopy

CIDNP (chemically induced dynamic nuclear polarization) NMR experiments were carried out on a 200 MHz Bruker AVANCE DPX spectrometer equipped with a custom-made CIDNP probe head. A Quantel Nd-YAG Brilliant B laser (355 nm, ∼50 mJ per pulse, pulse length 8–10 ns) operating at 20 Hz was employed as the light source. The pulse sequence of the experiment consists of a series of 180° radio-frequency (RF) pulses to suppress the NMR signals of the parent compounds, the laser flash, the 90° RF detection pulse, and the acquisition of the free induction decay (FID). "Dummy" CIDNP spectra employing the same pulse sequence but without the laser pulse were always measured. Samples were prepared in toluene-d_8 and deoxygenated by bubbling with nitrogen before the experiment. Chemical shifts (δ) are reported in ppm relative to tetramethylsilane or using the residual methyl signal of deuterated toluene as an internal reference (δH = 2.08 ppm). If necessary, line broadening (1 Hz, exponential) was applied to the spectra.

^{1}H, ^{13}C, and ^{29}Si – NMR spectra were recorded either on a Varian INOVA 400, Varian INOVA 300, a 200 MHz Bruker AVANCE DPX or a Bruker Avance 300 MHz spectrometer in benzene-d_6 and referenced versus tetramethylsilane using the internal ^{2}H-lock signal of the solvent.

Mass spectrometry

Mass spectra were run on an HP 5971/A/5890-II GC/MS coupling (HP 1 capillary column, length 25 m, diameter 0.2 mm, 0.33 mm poly(dimethylsiloxane). HRMS spectra were run on a Kratos Profile mass spectrometer.

DFT Calculations

Density functional theory was used for the theoretical calculations in this paper using the PBEh-3c functional,[4] which is a composite method including the PBE0 functional with additional dispersion applying Grimme's D3 method with Becke-Johnson damping plus geometrical counterpoise method to account for the basis set superposition error. The PBEh-3c method applies the def2-mSVP basis set. The program ORCA5.0.3 [5] was used for the calculations applying standard parameters. The geometry of **3** was optimized from the crystal structure. All other geometries were designed by the respective dissociation, rearrangement, or Brook rearrangement of various groups (TMS, $Si(TMS)_3$) towards the next or next-neighboring carbonyl group. The harmonic frequencies were calculated to confirm all minima and transition states on the potential energy surface. IR spectra were generated by broadening the harmonic frequencies by a Lorentzian of 4 cm^{-1} width, which is the experimental broadening. To account for anharmonicity, the peaks were then scaled with respective factors below 1500 cm^{-1} (0.93) and above 1500 cm^{-1} (0.9021) to best compare with the available experimental FT-IR spectra. For the optimized geometries of **3** and **4**, the UV spectra were generated after vertical excitation of 15 states by applying the time-dependent DFT (TDDFT) method using the PBEh-3c method and Gaussian broadening of 15 nm. The relevant orbitals were depicted by the program gabedit[6] with contour values of 0.05 a.u. To localize the transition states (TS) on the reaction path **3→5**, the climbing-image nudged elastic band (CI-NEB) method as implemented in ORCA5.0.3 was used. For the 10 points (including both minima) on the NEB reaction pathway, single point energy calculations were then performed to estimate the excited state on the CI-NEB pathway, including the TS.

ADC(2) Calculations

To further investigate the mechanism, and for the optimization of the biradicalic second excited singlet state, a multireference method must be used. Starting from the DFT minimum of **3,** ground state geometries were optimized with the Møller-Plesset second order method (MP2) using the cc-pVDZ basis set.[7] To unravel the photochemical mechanisms, we have performed extensive excited-state computations using the algebraic diagrammatic construction second-order (ADC(2)) method[8] with the cc-pVDZ basis set.(1) ADC(2) provides excited state properties comparable to the coupled cluster singles and doubles methods (CC2) at a significantly lower computational cost. The excited S1, S2 and T1 states were optimized with the spin opposite scaling approach of ADC(2) (SOS-ADC(2)) with the same basis set. Excited states have been characterized in terms of natural transition orbitals,[9] computed by retaining only singly excited coefficients of the ADC(2) wave functions.[10] ADC(2) calculations were performed with the quantum chemistry packages Turbomole 7.5.[11]

Experimental Procedures

Synthesis of 3 with diphenyl oxalate

To a solution of 5.00 g tetrakis(trimethylsilyl)silane (15.58 mmol; 1.00 eq.) in 50 mL THF, 11.43 mL (17.14 mmol; 1.10 eq.) methyllithium-lithiumbromide complex solution 1.5M in diethyl ether was added via a syringe at room temperature. The solution was stirred for one hour. After full conversion (monitored by ^{29}Si NMR-spectroscopy with a D_2O capillary) the reaction solution was added to 1.93 g diphenyl oxalate (7.80 mmol; 0.50 eq.) in 50 mL THF at -30° and afterwards allowed to warm up to room temperature. The solution was added to 200 mL of saturated NH_4Cl solution. After phase separation, three-fold washing of the aqueous phase with 100 mL of Et_2O, drying of the combined organic layers with Na_2SO_4 and evaporation of the solvents in vacuum, the product was purified via flash column chromatography (*n*-heptane) to result a blue crystalline solid.

Yield: 480 mg (871 µmol; 11 %) of analytically pure **3** as blue crystalline solid.

Synthesis of 3 with oxalyl chloride

To a solution of 1.00 g tetrakis(trimethylsilyl)silane (3.12 mmol; 1.00 eq.) in 30 mL THF, 385 mg (3.34 mmol; 1.10 eq.) potassium tert-butoxide was added. The solution was stirred for one hour. After full conversion (monitored by ^{29}Si NMR-spectroscopy with a D_2O capillary) the reaction solution was rapidly added to 147 µl oxalylchloride (1.71 mmol; 0.55 eq.) in 20 mL THF at –100°C. Subsequently, 150 mL of 10% H_2SO_4 solution were added immediately and the reaction solution was allowed to warm up to room temperature. After phase separation, three-fold washing of the aqueous phase with 60 mL of Et_2O, drying of the combined organic layers with Na_2SO_4 and evaporation of the solvents in vacuum, the product was purified via flash column chromatography (*n*-heptane) to result a blue crystalline solid.

Yield: 171 mg (0.31 mmol; 20 %) of analytically pure **3** as blue crystalline solid.
mp: 130-132 °C. **Anal. Calc.** (%) for $C_{20}H_{54}O_2Si_8$: C, 43.57; 9.87 H, Found: C, 43.46; H, 9.83. **^{1}H-NMR** Data (benzene-d_6, tetramethylsilane, ppm): 0.32 (s, 54H,-Si-(C*H*$_3$)$_3$).**^{13}C-NMR** Data (benzene-d_6, tetramethylsilane, ppm): 238.90 (*C*=O), 1.62 (-Si-(*C*H$_3$)$_3$). **^{29}Si-NMR** Data (benzene-d_6, tetramethylsilane, ppm): –10.7 (-*Si*Me$_3$), –79.0 (*Si*-SiMe$_3$). **UV/Vis**: λ [nm] (ε [mol^{-1} dm^3 cm^{-1}]) = 637 (140), 317 (2460), 247 (14650). **IR** (neat): ν(C=O) = 1635 cm^{-1}.

Synthesis of 4

An NMR tube was charged with 85 mg **3** (154 µmol; 1.00 eq.) and dissolved in 700µl of benzene-d_6. The NMR-tube was irradiated at 365 nm for 45 minutes. After full conversion (monitored by ^{1}H-NMR spectroscopy), the solvent of the sample was evaporated and the crude product was recrystallized from *n*-pentane.
Yield: 76 mg (138 µmol; 89 %) of analytically pure **4** as pink crystalline solid.
mp: 111 °C. **Anal. Calc.** (%) for $C_{20}H_{54}O_2Si_8$: C, 43.57; 9.87 H, Found: C, 43.41; H, 9.90. **^{1}H-NMR** Data (benzene-d_6, tetramethylsilane, ppm): 0.33, 0.29 (s, each 27H,-(Si-(C*H*$_3$)$_3$)$_3$). **^{13}C-NMR** Data (benzene-d_6, tetramethylsilane, ppm): 219.04 (*C*=O), 64.36 (-*C*=C=O), 1.22, 0.56 (-(Si-(*C*H$_3$)$_3$)$_3$). **^{29}Si-NMR** Data (benzene-d_6, tetramethylsilane, ppm): 19.0 (-O-*Si*-(SiMe$_3$)$_3$), −11.8 (-Si-(*Si*Me$_3$)$_3$), −15.8 (-O-Si-(*Si*Me$_3$)$_3$), −77.0 (-*Si*-(SiMe$_3$)$_3$. **UV/Vis**: λ [nm] (ε [mol^{-1} dm^3 cm^{-1}]) = 528 (82), 279 (25432). **IR** (neat): ν(C=C=O) = 2042 cm^{-1}. **GC-MS** [m/e (relative intensity)] 550.3 (12.8%, M$^+$), 315.1 (22.1%, $[Si_5C_{11}H_{27}O]^+$), 190.9 (17.6%, $[OSi(SiMe_3)_2]^+$), 147.1 (53.9%, $[(SiMe_3)_2]^+$), 116.9 (13.8%, $[OSiSiMe_3]^+$), 73.0 (100.0%, $[SiMe_3]^+$)

Synthesis of 5

An NMR tube was charged with 104 mg **3** (189 µmol; 1.00 eq.) and dissolved in 700µl of benzene-d_6. The NMR-tube was irradiated at 590 nm for 60 minutes. After full conversion monitored by NMR spectroscopy, the solvent of the sample was evaporated yielding the product as yellowish oil.

Yield: 104 mg (189 µmol; 100 %) of analytically **5** as yellowish oil.
Anal. Calc. (%) for $C_{20}H_{54}O_2Si_8$: C, 43.57; 9.87 H, Found: C, 43.73; H, 9.88. **^{1}H-NMR** Data (benzene-d_6, tetramethylsilane, ppm): 0.48 (s, 27H, -C-Si-(Si-(C*H*$_3$)$_3$)$_3$), 0.29 (s, 9H, -O-Si-(C*H*$_3$)$_3$), 0.16 (s, 18H, -Si-(Si-(C*H*$_3$)$_2$).**^{13}C-NMR** Data (benzene-d_6, tetramethylsilane, ppm): 142.38 (-O-*C*=C-), 136.29 (-O-C=*C*-), 2.23 (-Si-(Si(*C*H$_3$)$_3$)$_3$), 2.14 (-O-Si(*C*H$_3$)$_3$), −1.42 (-Si-(Si(*C*H$_3$)$_3$)$_2$). **^{29}Si-NMR** Data (benzene-d_6, tetramethylsilane, ppm): 13.6 (-O-*Si*Me$_3$), −12.1 (-Si-(*Si*Me$_3$)$_3$), −14.6 (-Si-(*Si*Me$_3$)$_2$), −25.6 (-*Si*-(SiMe$_3$)$_2$), −80.3 (-*Si*-(SiMe$_3$)$_3$). **IR** (neat): ν(Si-O) = 1244 cm^{-1}. **GC-MS** [m/e (relative intensity)] 550.2 (12.2%, M$^+$), 315.1 (14.3%, $[Si_5C_{11}H_{27}O]^+$), 190.9 (14.7%, $[OSi(SiMe_3)_2]^+$), 147.1 (58.8%, $[(SiMe_3)_2]^+$), 73.0 (100.0%, $[SiMe_3]^+$),

Photolysis Experiments of 3 in the presence of MeOH at 365 nm

An NMR tube was charged with 110 mg **3** (200 µmol; 1.00 eq.), 203 µl MeOH (5.11 mmol, 25 eq.), 3 drops of Et_3N (catalytic amount) and 500µl of benzene-d_6. The NMR-tube was irradiated at 365 nm for 60 minutes. After full conversion from **3** to **4**, monitored by ^{1}H-NMR spectroscopy, the sample was stored in the absence of light and air for 7 days. After full conversion of **4** with MeOH, monitored by ^{1}H-NMR spectroscopy, the solvents were evaporated and the crude product was recrystallized from n-pentane.

Yield: 95 mg (162 µmol; 81.32 %) of analytically **6** as colorless crystalline solid.
mp: 114-118 °C. **Anal. Calc.** (%) for $C_{21}H_{58}O_3Si_8$: C, 43.24; 10.02 H, Found: C, 43.10; H, 9.98. **^{1}H-NMR** Data (benzene-d_6, tetramethylsilane, ppm): 4.59 (s, 1H, -C*H*, 3.35 (s, 3H, -OC*H*$_3$), 0.38, 0.31 (s, each 27H, -(Si-(C*H*$_3$)$_3$)$_3$. **^{13}C-NMR** Data (benzene-d_6, tetramethylsilane, ppm): 175.60 (-*C*=O), 70.39 (-*C*-C=O), 50.82 (-O-CH$_3$), 1.90 (-C-Si-(Si-(*C*H$_3$)$_3$)$_3$), 1.63 (-O-Si-(Si-(*C*H$_3$)$_3$)$_3$). **^{29}Si-NMR** Data (benzene-d_6, tetramethylsilane, ppm): 7.8 (-O-Si(*Si*Me$_3$)$_3$), −12.1 (-Si-(*Si*Me$_3$)$_3$), −15.7 (-O-*Si*-(*Si*Me$_3$)$_3$), −65.9 (-*Si*-(SiMe$_3$)$_3$). **IR** (neat): ν(C=O) = 1735 cm^{-1}. **GC-MS** [m/e (relative intensity)] 509.3 (100.0%, [M$^+$-SiMe$_3$]), 205.0 (58.9%, $[Si_2C_8H_{21}O_2]^+$), 173.0 (22.3%, $[Si_3C_6H_{18}]^+$), 131.0 (8.1%, $[Si_2C_4H_{12}O]^+$), 73.0 (82.4%, $[SiMe_3]^+$).

Photolysis Experiments of 3 in the presence of MeOH at 590 nm

Two NMR tubes were charged with 186 mg **3** (337 µmol; 1.00 eq.), 205 µl MeOH (5.06 mmol, 15.00 eq.), 3 drops of Et_3N (catalytic amount) and 850µl of benzene-d_6, respectively. The NMR-tubes were irradiated at 590 nm for 120 minutes. After full conversion monitored by ^{1}H-NMR spectroscopy, revealing a *cis-trans* isomerism in a ratio of 2:1, the solvents of the samples were evaporated and the main isomer (*Isomer 2*) was isolated via preparative thin-layer chromatography (*n*-pentane).

Isomer 1:
^{1}H-NMR Data (benzene-d_6, tetramethylsilane, ppm): 7.16 (s, 1H, =C-*H*, superimposed by benzene-d_6), 3.34 (s, 3H, -O-C*H*$_3$), 0.39 (s, 27H, -Si-(Si-(C*H*$_3$)$_3$)$_3$), 0.34 (s, 9H, -O-Si-(C*H*$_3$)$_3$), 0.32 (s, 18H, -Si-(Si-(C*H*$_3$)$_3$)$_2$). **^{13}C-NMR** Data (benzene-d_6, tetramethylsilane, ppm): 141.60 (-O-*C*=C-H), 138.82 (-O-C=*C*-H), 53.84 (-O(*C*H$_3$)), 3.05 (-Si-(Si(*C*H$_3$)$_3$)$_3$), 2.53 (-O-Si(*C*H$_3$)$_3$), 0.53 (-Si-(Si(*C*H$_3$)$_3$)$_2$). **^{29}Si-NMR** Data (benzene-d_6, tetramethylsilane, ppm): 9.1 (-O-*Si*Me$_3$), 6.7 (-O-*Si*-(SiMe$_3$)$_2$), -13.2 (-Si-(*Si*Me$_3$)$_3$), -21.1 (-O-Si-(*Si*Me$_3$)$_2$), -80.7 (-*Si*-(SiMe$_3$)$_3$).

Isomer 2:
Yield: 119 mg (203 µmol; 30.11 %) of analytically **7a** as colorless oil.
Anal. Calc. (%) for $C_{21}H_{58}O_3Si_8$: C, 43.24; 10.02 H, Found: C, 43.39; H, 10.03.

^{1}H-NMR Data (benzene-d_6, tetramethylsilane, ppm): 7.27 (s, 1H, =C-*H*), 3.52 (s, 3H, -O-C*H*$_3$), 0.41 (s, 27H, -Si-(Si-(C*H*$_3$)$_3$)$_3$), 0.28 (s, 9H, -O-Si-(C*H*$_3$)$_3$), 0.28 (s, 18H, -Si-(Si-(C*H*$_3$)$_3$)$_2$). ^{13}C-NMR Data (benzene-d_6, tetramethylsilane, ppm): 141.60 (-O-*C*=C-H), 138.82 (-O-C=*C*-H), 53.49 (-O(*C*H$_3$)), 2.23 (-Si-(Si(*C*H$_3$)$_3$)$_3$), 1.39 (-O-Si(*C*H$_3$)$_3$), -0.69 (-Si-(Si(*C*H$_3$)$_3$)$_2$). ^{29}Si-NMR Data (benzene-d_6, tetramethylsilane, ppm): 15.48 (-O-*Si*Me$_3$), 14.2 (-O-*Si*-(SiMe$_3$)$_2$), -12.4 (-Si-(*Si*Me$_3$)$_3$), -22.3 (-O-Si-(*Si*Me$_3$)$_2$), -84.2 (-*Si*-(SiMe$_3$)$_3$). **HSQC-NMR** Data (benzene-d_6, tetramethylsilane, ppm) [^{1}H-signal, ^{13}C-signal]: 7.27, 138.24 (=*C-H*), 3.51, 52.98 (-O-*CH*$_3$), 0.41, 2.71 (-Si-(Si-(*CH*$_3$)$_3$)$_3$), 0.27, 1.02 (-O-Si-(*CH*$_3$)$_3$), 0.26, -1,16 (-Si-(Si-(*CH*$_3$)$_3$)$_2$). **IR** (neat): ν(Si-O) = 1241 cm^{-1}. **GC-MS** [m/e (relative intensity)] 73.0 (100.0%, $SiMe_3$), 205.1 (83.8%, $(SiMe_3)_2SiOMe$), 147.1 (47,5%, $(SiMe_3)_2$ +2H), 175.1 (37.9%, $Si(SiMe_3)_2$ +2H), 117.0 (31.6%, $OSiSiMe_3$), 131.1 (31.5%, $COSiSiMe_3$ +2H), 279 (29.7%, $(SiMe_3)_3SiOMe$), 221.1 (28.5%, $OSi(SiMe_3)_2(OMe)$), 191.1 (19.6%, $OSi(SiMe_3)_2$ +H).

Photochemical Investigation of 3

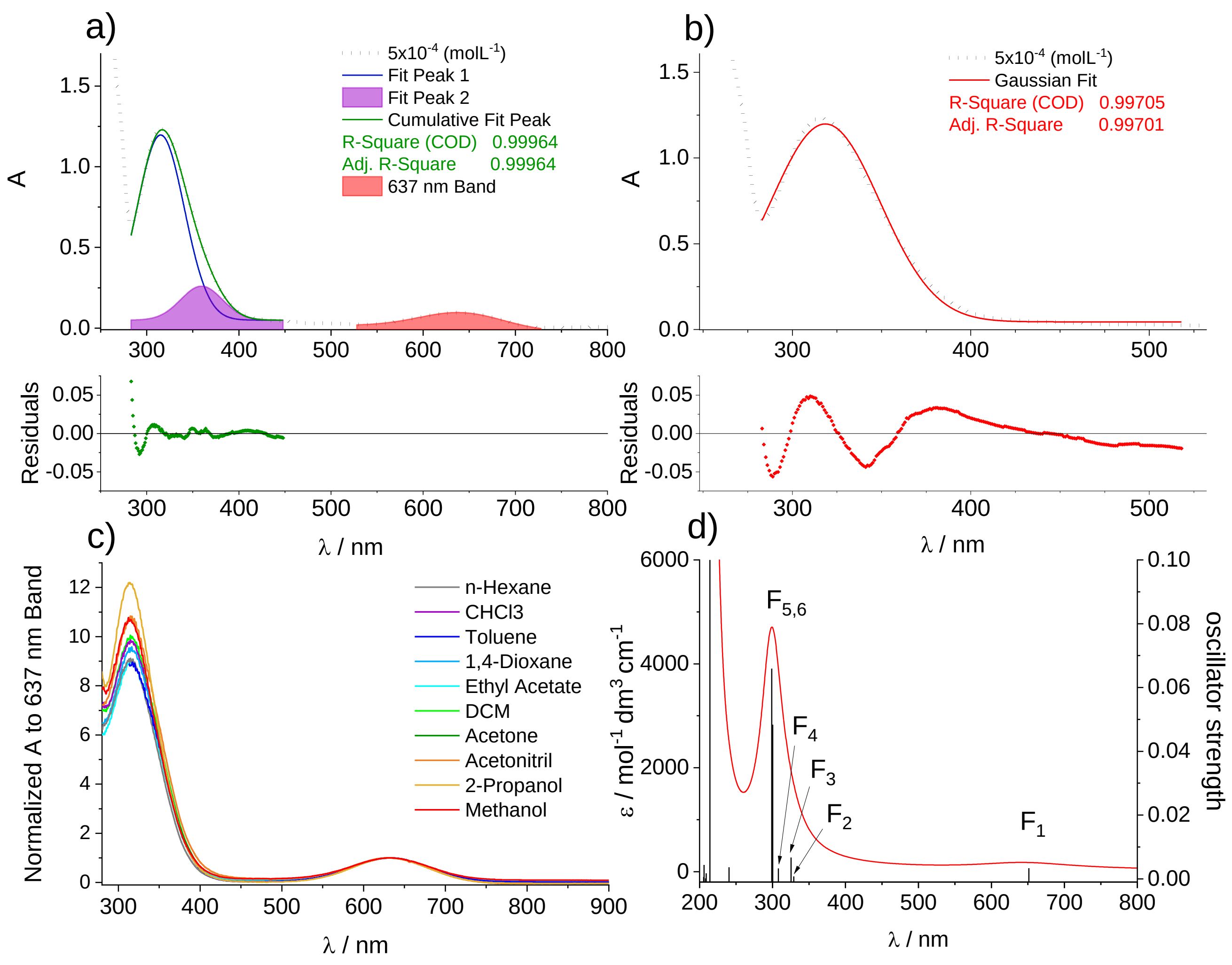

Figure S1. Deconvoluted experimental UV/vis spectrum of **3**. a) Fitting two gaussian peaks for 315 nm band (Blue Fit Peak 1 maximum at 315 nm, assigned to various $\sigma_{Si} \rightarrow \pi_{C=O}{}^*$ transitions and Purple Fit Peak 2 with maximum at 360 nm, assigned to $n \rightarrow \pi^*$ transition. b) Fitting only one gaussian peak for 317 nm band. Comparing residuals in a) and b) shows that deconvolution of 317 nm band with two gaussian peaks fits better to the experimental data. c) Solvent dependence of **3**, normalized intensity to the long wavelength absorption band, and d) calculated UV/vis spectrum of **3** with assignment of the vertical excitations.

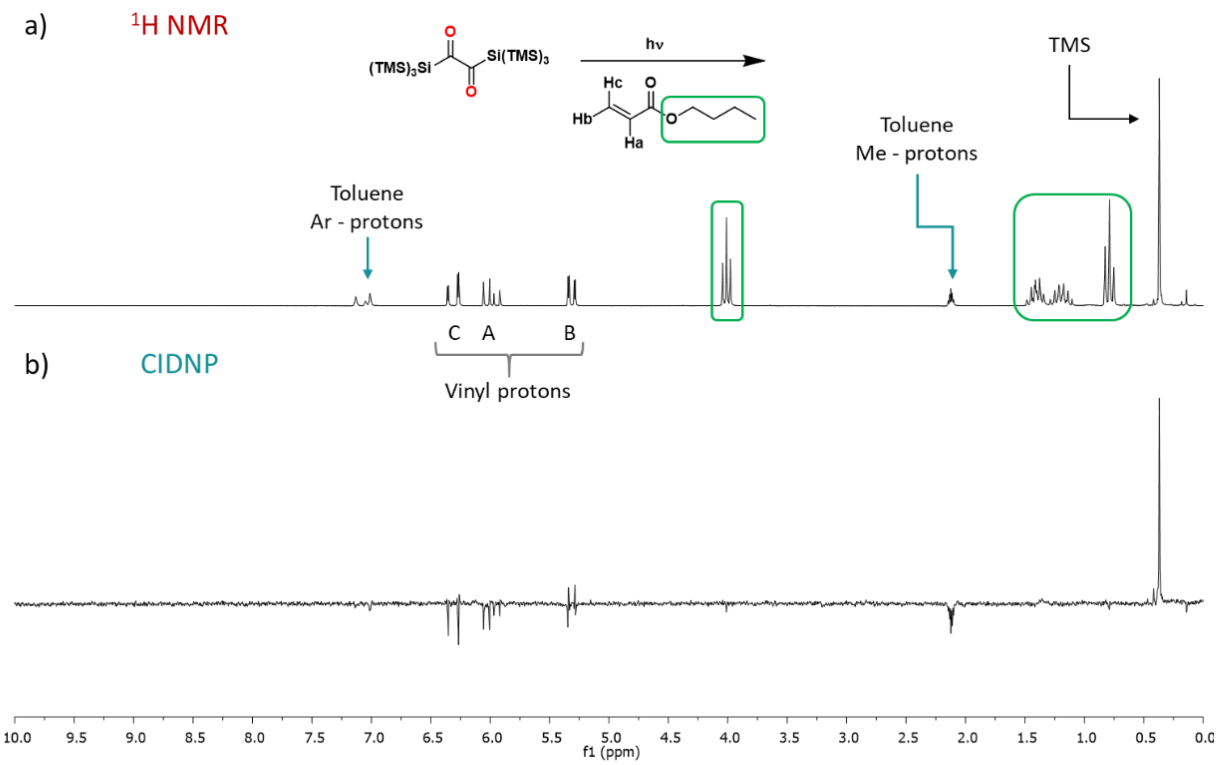

Figure S2. a) ^{1}H-NMR and b) CIDNP spectrum (excitation at λ = 355 nm, ca. 50 mJ per pulse) of **3** in the presence of butyl acrylate (10 mM **4**, 50 mM BA in toluene-d_8). All of the polarizations visible in CIDNP spectra are present in the dummy spectra as well.

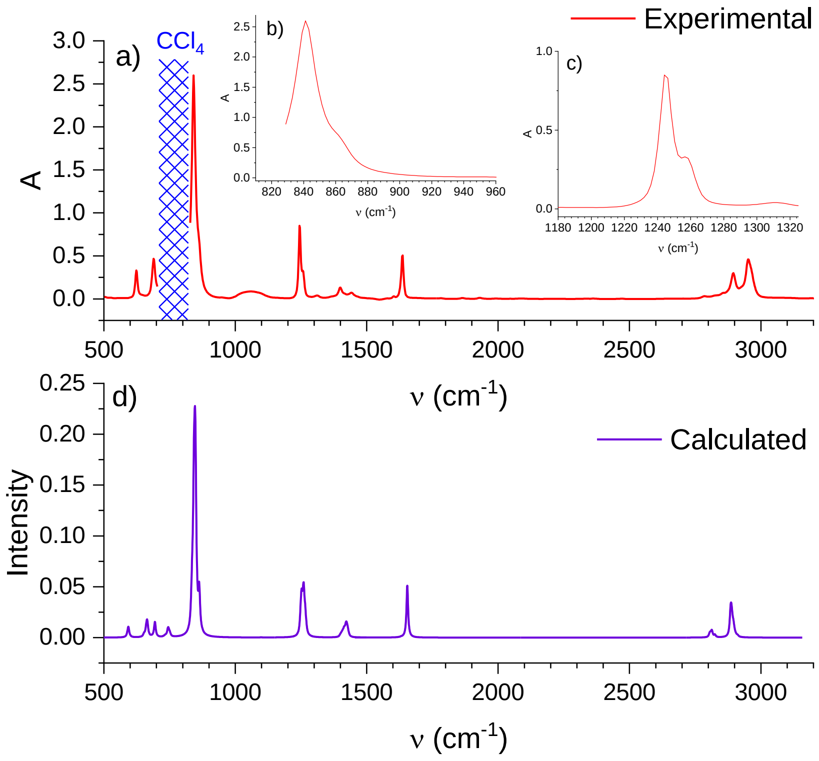

Figure S3. a) The Steady-State FT-IR spectra of **3** (0.05 molL^{-1}) in CCl_4 (solvent subtracted), 711 – 827 cm^{-1} range is inaccessible due to the strong solvent absorption, b) zoomed view of 810 – 960 cm^{-1} range, c) zoomed view of 1180 – 1325 cm^{-1} range, d) The calculated IR spectrum of **3**.

Irradiation of 3 with light source having an emission center at 360 - 365 nm

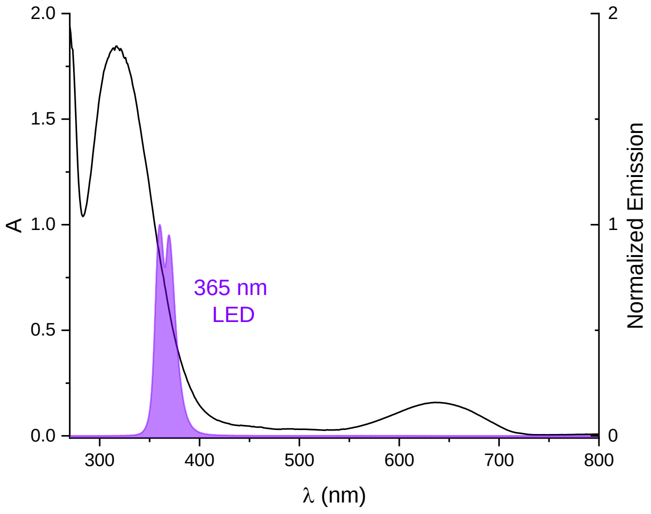

Figure S4. UV/Vis spectra of **3** overlapped with the normalized emission spectra of LED having an emission center at 365 nm.

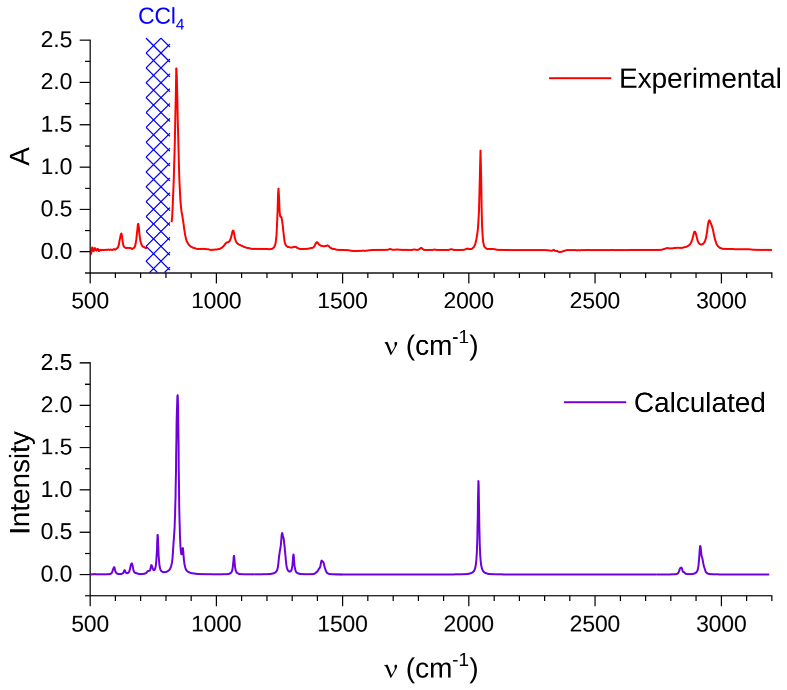

Figure S5. a) The experimental FT-IR spectra of **4** in CCl_4 (solvent subtracted), b) the calculated IR spectrum of **4**.

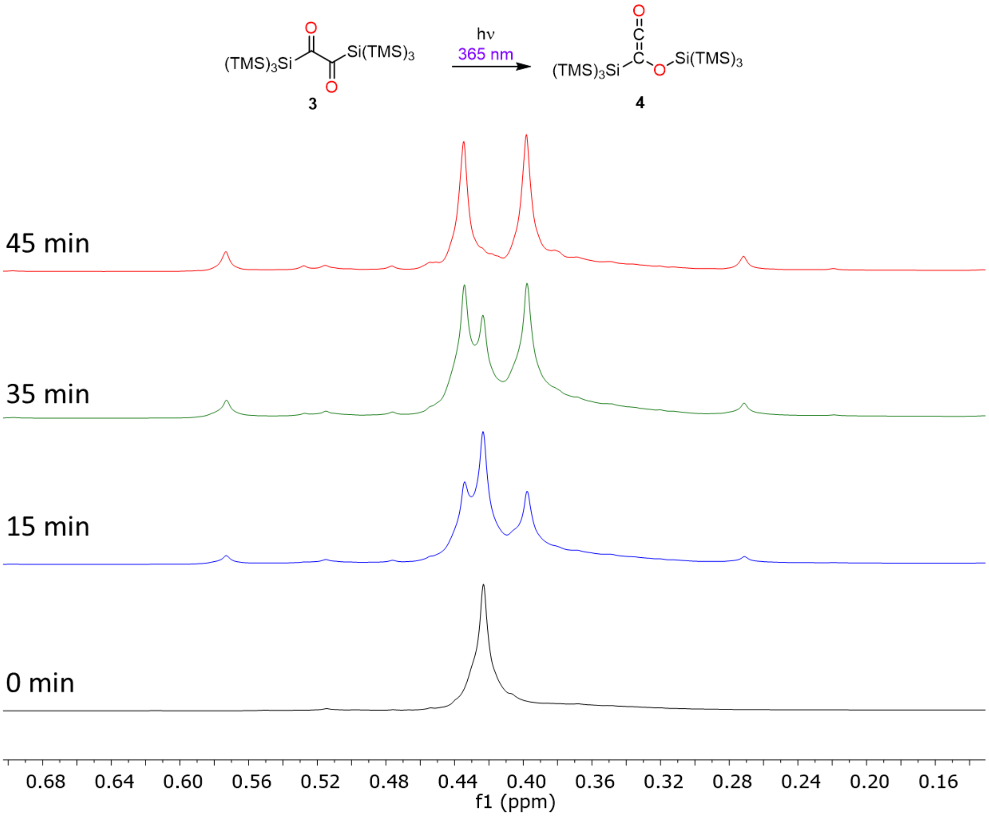

Figure S6. The ^{1}H NMR spectra for the product formation of **4** during the irradiation of **3** with LED with an emission maximum at 365 nm (benzene-d_6 solution, vs ext. tetramethylsilane, ppm).

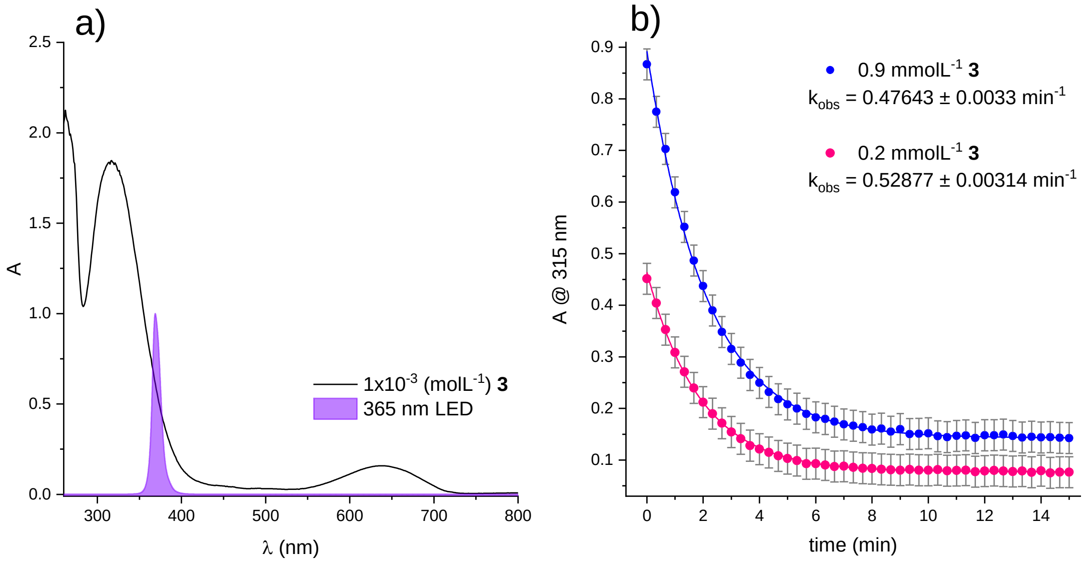

Figure S7. a) Overlap of UV/Vis spectra of **3** with used LED (emission maxima cantered at 365 nm). b) Absorbance at 315 nm *vs* time for the irradiation of **3** at two different concentrations (0.9 mM and 0.2 mM) with LED having an emission maximum at 365 nm. n-hexane used as a solvent.

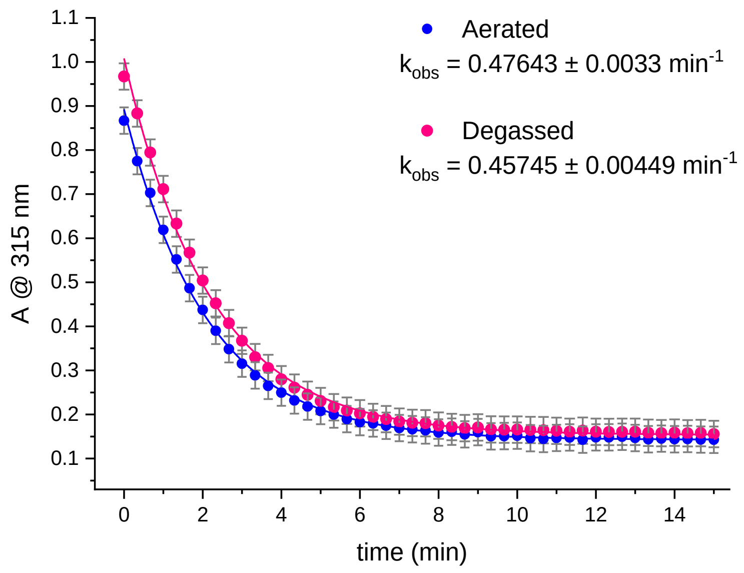

Figure S8. Absorbance at 315 nm vs. time for the irradiation of **3** of the degassed sample (argon purged for 5 min) and aerated sample, with LED having an emission maximum at 365 nm. *n*-hexane used as a solvent.

The experiment evaluating the dependence of **3**→**4** rate on temperature was performed in a custom-made setup depicted on Figure S9. Briefly light from the LED (50 W rated electrical power, supplied by 34V/1A using Rigol DP821 power supply, emission maximum cantered at 365 nm, emission shown on Figure S7) was coupled to the liquid waveguide using the aspheric condenser lens. The light was further guided through the quart rod with 45° angle cut at the bottom with aluminum mirror mounted at the end. Purpose designed cuvette holder fixing the quartz rod, mirror and the cuvette in place was 3D printed and additionally coated with optical Teflon on the inner side. The cuvette holder is immersed in a cooling bath, temperature of which is measured using the digital thermometer. The temperature was adjusted using either room temperature acetonitrile or liquid nitrogen cooled mixture of liquid and solid acetonitrile (for -45°), or methanol (for -85°C). the purpose of the custom setup was to ensure constant and reproducible light intensity and wavelength (the emission spectra of LED strongly depends on the temperature) independent on the temperature. The sample in the cuvette, after thermal equilibration in the bath, was irradiated for the desired time followed by taking it out from the thermal bath (if necessary, it was warmed up to the room temperature) and measured the UV/Vis spectra. This procedure was repeated for all of the time and temperature points measured. All of the sample manipulations were performed under low intensity red light.

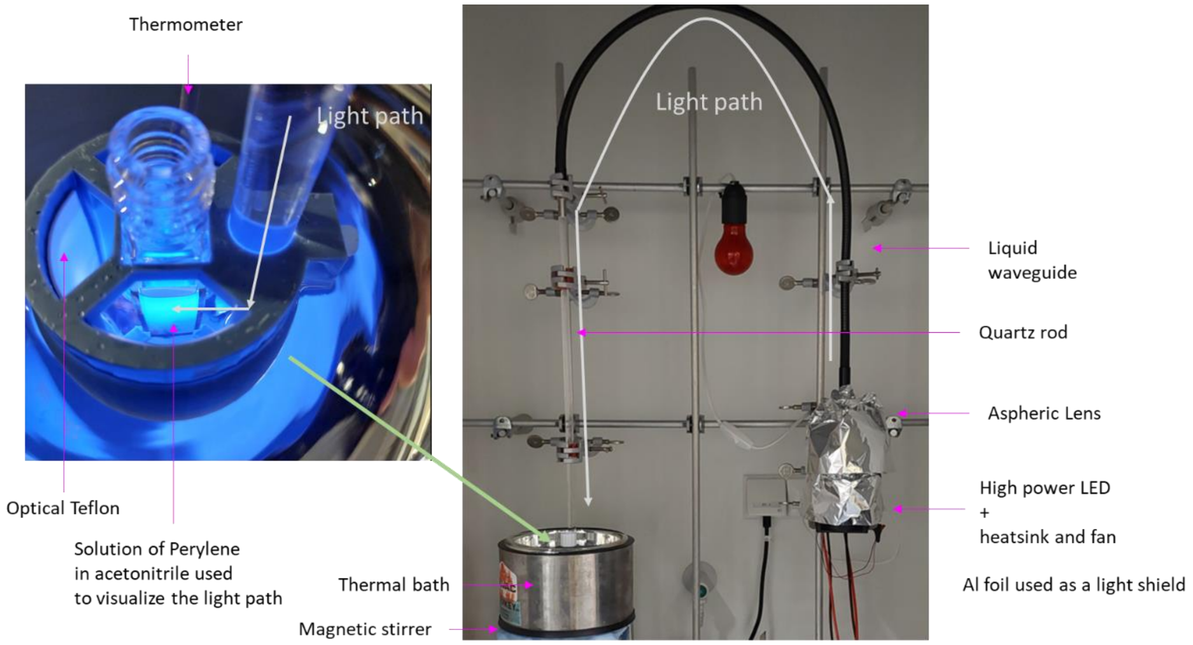

Figure S9. The experimental setup used for temperature dependent kinetic studies.

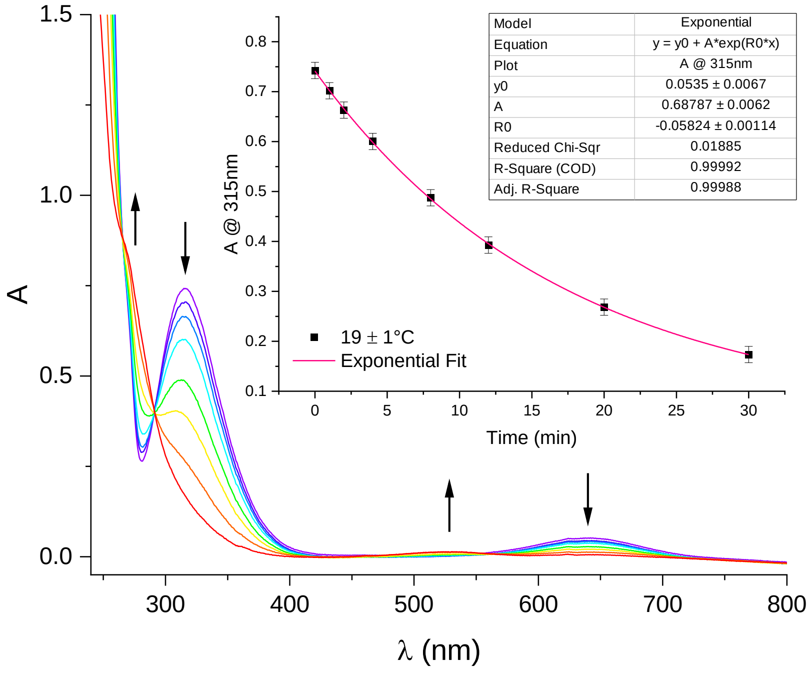

Figure S10. UV/Vis spectra vs. time for the irradiation of **3** at 19 ± 1°C (non-degassed sample, *n*-pentane as a solvent), with LED having an emission maximum at 365 nm. Note isosbestic point at 292 nm (indicating **3** to **4** conversion without intermediates). Insert shows absorbance at 315 nm vs. time.

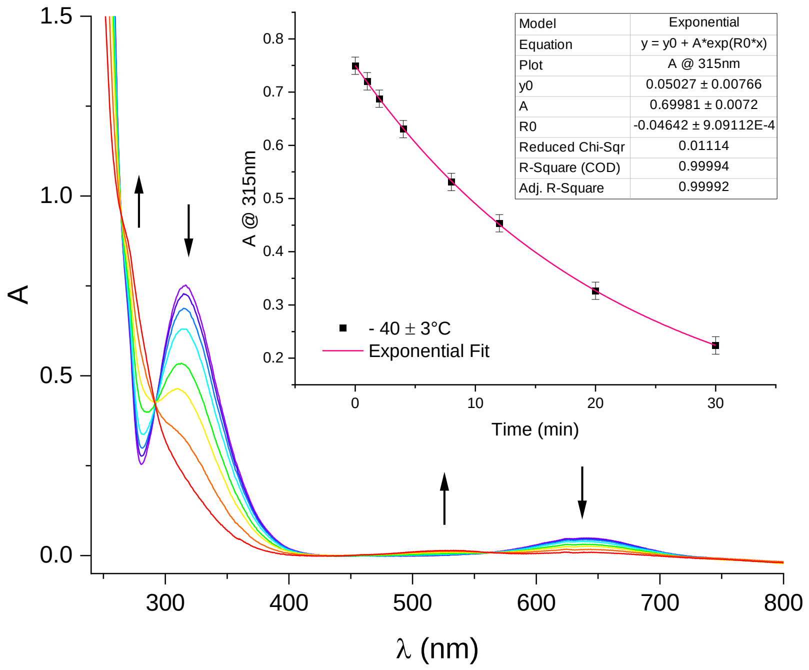

Figure S11. UV/Vis spectra vs. time for the irradiation of **3** at -40 ± 3°C (non-degassed sample, *n*-pentane as a solvent), with LED having an emission maximum at 365 nm. Note isosbestic point at 292 nm (indicating **3** to **4** conversion without intermediates). Insert shows absorbance at 315 nm vs. time.

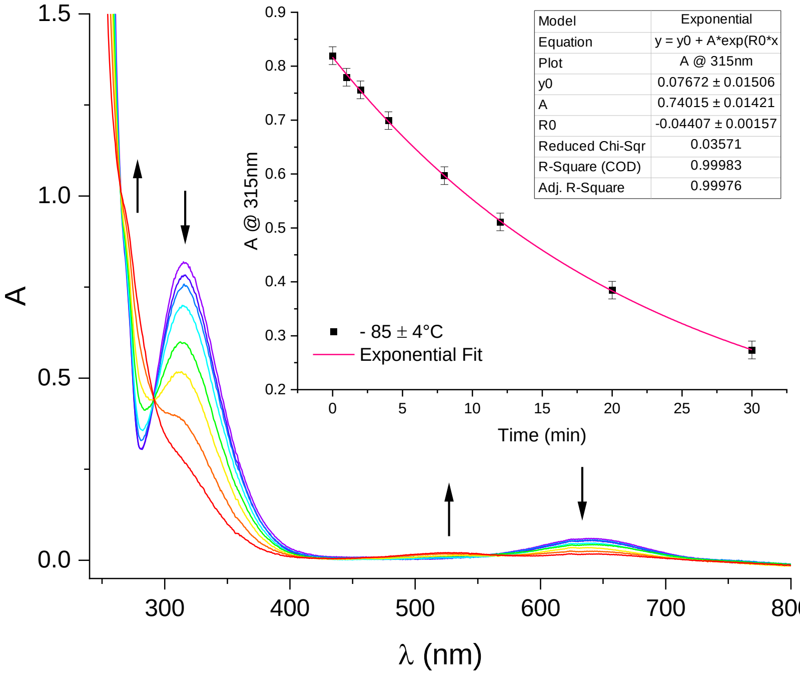

Figure S12. UV/Vis spectra vs. time for the irradiation of **3** at -85 ± 4°C (non-degassed sample, *n*-pentane as a solvent), with LED having an emission maximum at 365 nm. Note isosbestic point at 292 nm (indicating **3** to **4** conversion without intermediates). Insert shows absorbance at 315 nm vs. time.

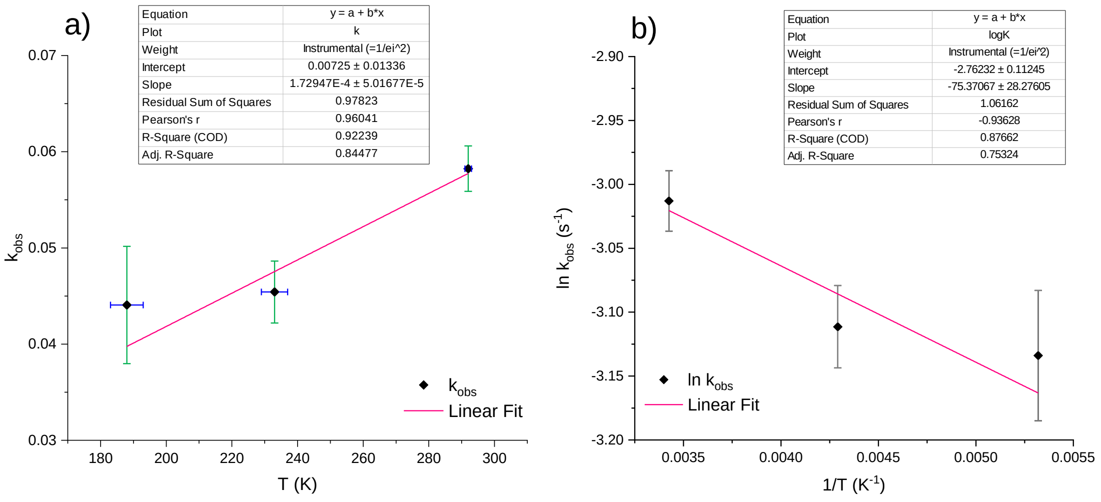

Figure S13. Temperature dependence for **3**→**4** conversion. A) Plot of k_{obs} vs. temperature in K, and b) Arrhenius plot of Ln k_{obs} vs. 1/T, from the plot E_a of 0.63 $KJmol^{-1}$ was obtained.

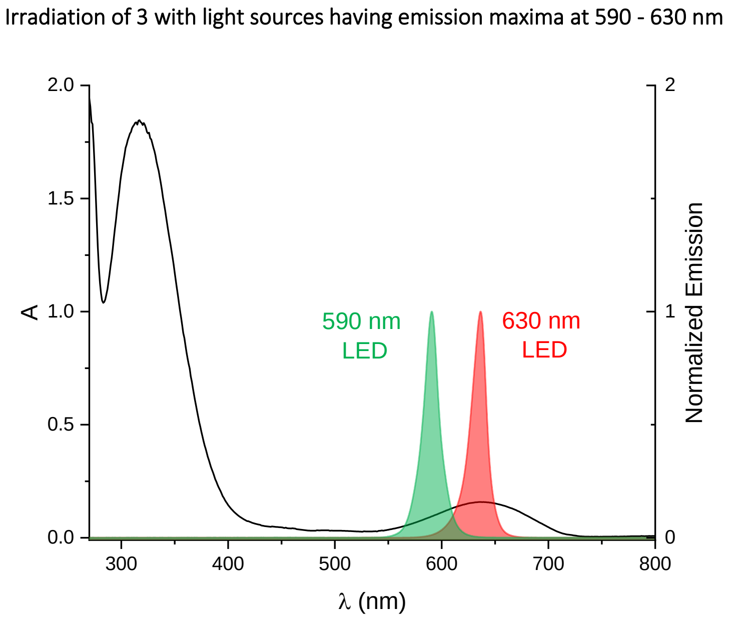

Figure S14. UV/Vis spectra of **3** overlapped with the normalized emission spectra of LED having an emission maximum of 590nm and 630 nm.

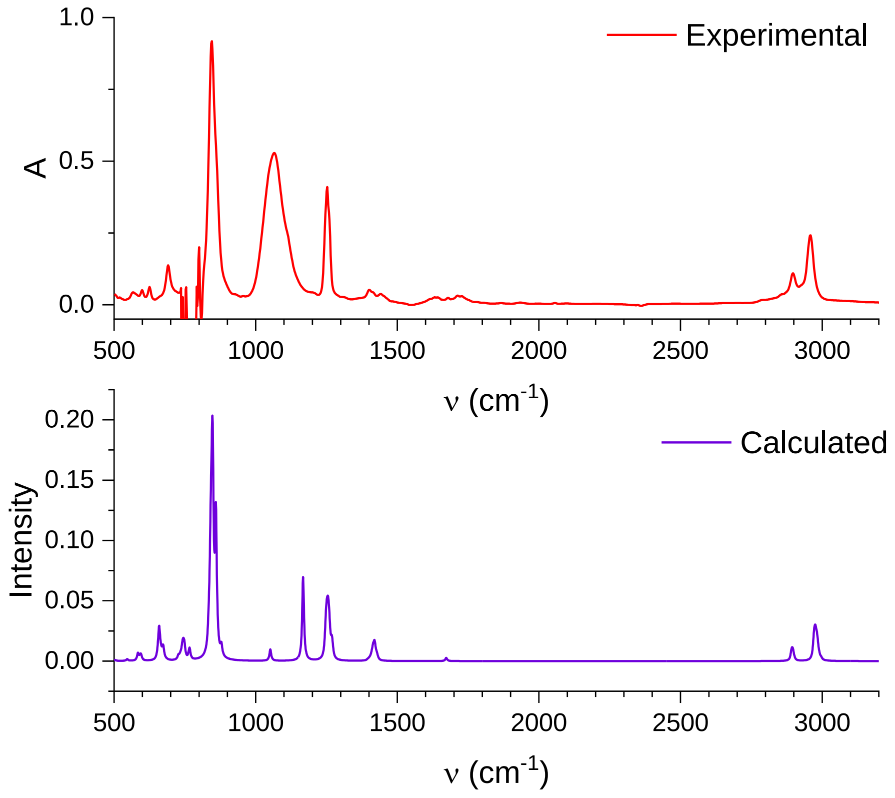

Figure S15. a) The experimental FT-IR spectra of **5** in CCl_4 (solvent subtracted), b) the calculated IR spectrum of **5**.

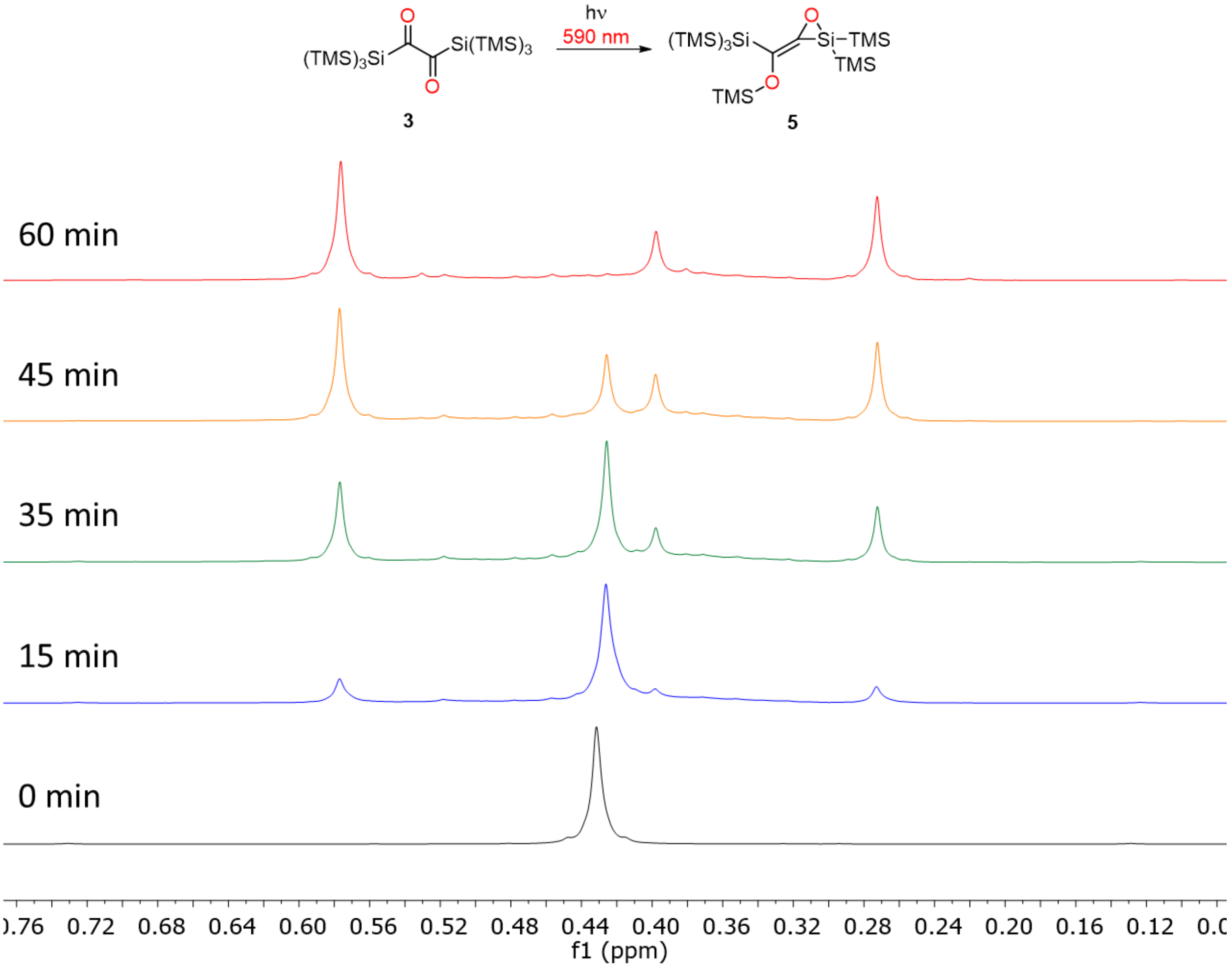

Figure S16. The ^{1}H-NMR spectra for the product formation **5** during the irradiation of **3** with LED having emission maxima at 590 nm (benzene-d6 solution, vs ext. tetramethylsilane, ppm).

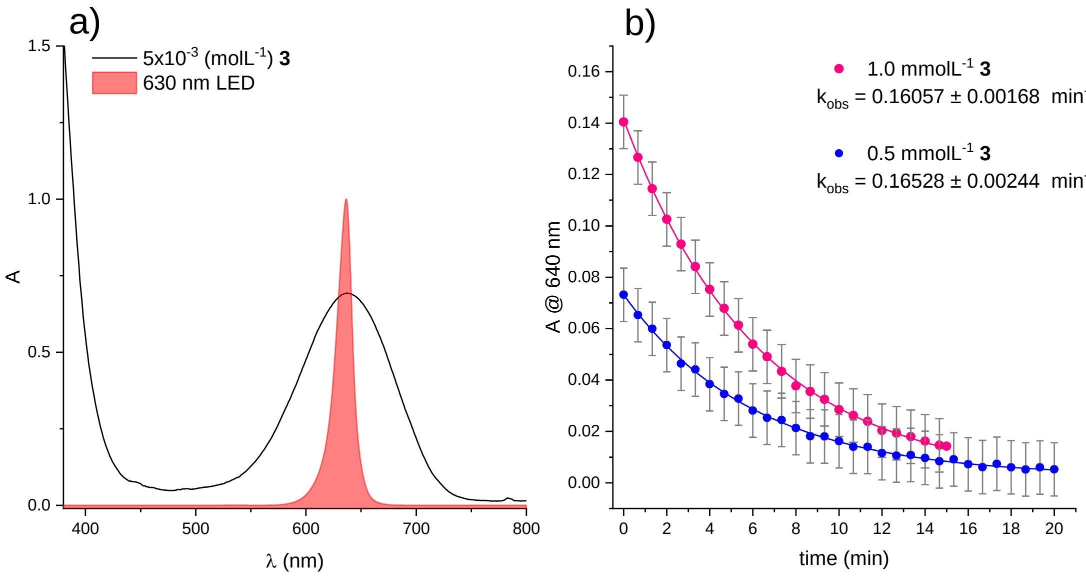

Figure S17. a) Overlap of UV/vis spectra of 3 with used LED (emission maxima cantered at 630 nm). b) absorbance at 640 nm vs. time for the irradiation of **3** at two different concentrations (1.0 mM and 0.5 mM) with LED having an emission maximum at 630 nm. *n*-Hexane used as a solvent.

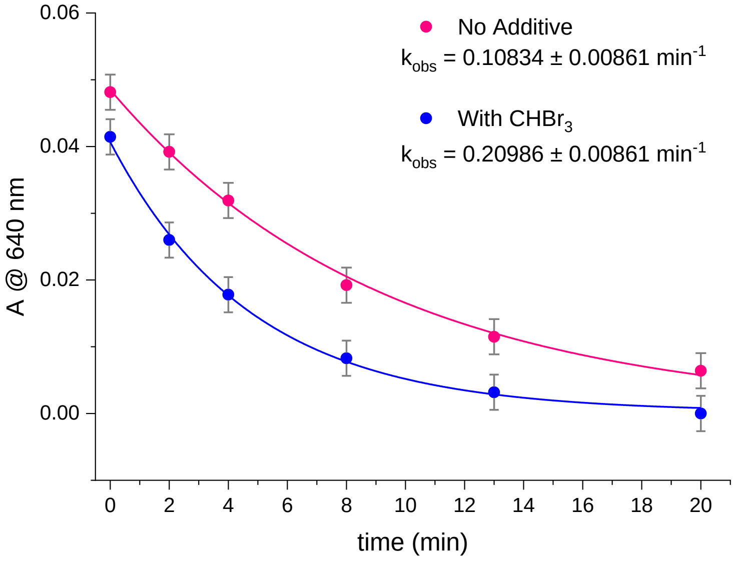

Figure S18. Absorbance at 640 nm vs. time for the irradiation of **3** (without any additive and with addition of 33% v:v of $CHBr_3$ to the solvent) of the degassed sample (argon purged for 5 min), with LED having an emission maximum at 630 nm. *n*-Hexane used as a solvent.

The triplet quenching experiment herein performed is based on the competition between the **3**→**5** conversion (Equation 1, Figure S19) and the energy transfer between **3** and the triplet quencher (Equation 2, Figure S19). The principle is described in details in the literature.[12] Briefly, the **3**→**5** conversion is initiated by irradiation of the solution with high power LED having an emission maximum centered at λ = 630 nm, the conversion is monitored by following the absorbance at λ = 640 nm vs time. The reaction solution contains between 0.3 – 0.4 molL^{-1} of **3** (depending on the experiment), in 3 mL of solvent (1:2, v:v $CHBr_3$/Hexane) and 0 eq (for control) or 10 eq of the respective triplet quencher (triplet quenchers do not show appreciable absorption at λ = 630 nm). The obtained absorbance at λ = 640 nm vs. time plots (showing exponential decrease of **3**) were used to calculate the rate of **3**→**5**. The schematic Jablonski diagram of the process is presented on Figure S20. The rate of **3**→**5** conversion was increased using the external heavy atom effect of $CHBr_3$ as a cosolvent (33 % v:v). Assuming the energy of triplet state of quencher higher than that of **3** the rate of **3**→**5** conversion will not be affected as the energy transfer is highly endergonic and thus is not expected to occur (blue Q in Figure S19a and blue line S19b). If on the other the triplet state energy of triplet quencher is below that of **3** upon population of the triplet state of **3** the energy transfer occurs to the triplet state of quencher (green Q in Figure S19a) thus depopulating the triplet state of **3** leading to the overall lower rate of **3**→**5** conversion (green line in Figure S19b). The intermediate case (black Q, Figure S19a and black line Figure S19b) is when the triplet energy of the quencher is approximately the same as that of **3** and the ratio between processes (1) and (2) is the same.

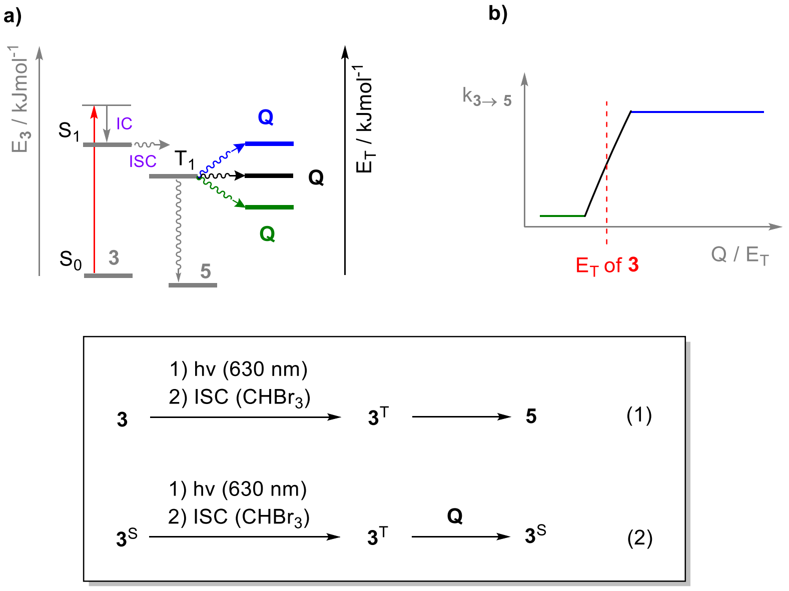

Figure S19. a) Schematic Jablonski diagram of triplet state quenching, b) schematic description of expected change in **3**→**5** rate vs. triplet state energy of the quencher. The equations of the two processes are sown below the figure.

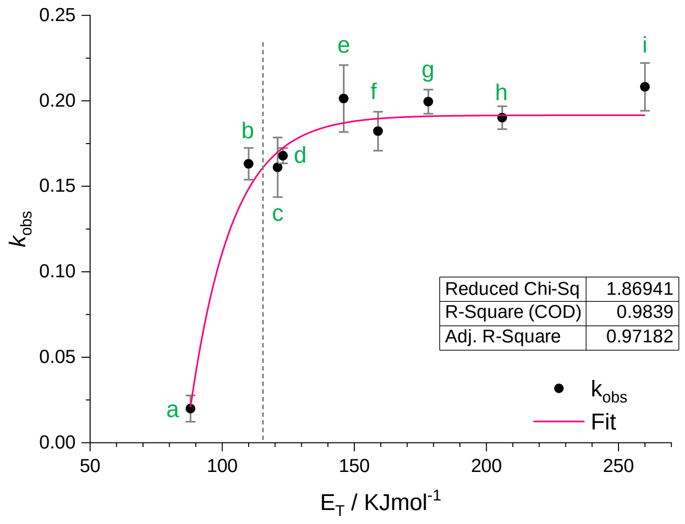

Figure S20. Triplet quenching, plot of k_{obs} vs. triplet energy of the quencher, T_Q (E_T / Kjmol^{-1}). Used triplet quenchers (a → i) with associated triplet energies in brackets: a = β-Carotene (88)[13], b = Rubrene (110)[14], c = *Z*-Azobenzene (121)[15], d = Tetracene (123)[16], e = Perylene (148)[17], f = Ferrocene (159)[13], g = Anthracene (178)[18], h = *E*-Stilbene (206)[19], and i = Phenanthrene (260)[15].

Photochemical Investigation of 4

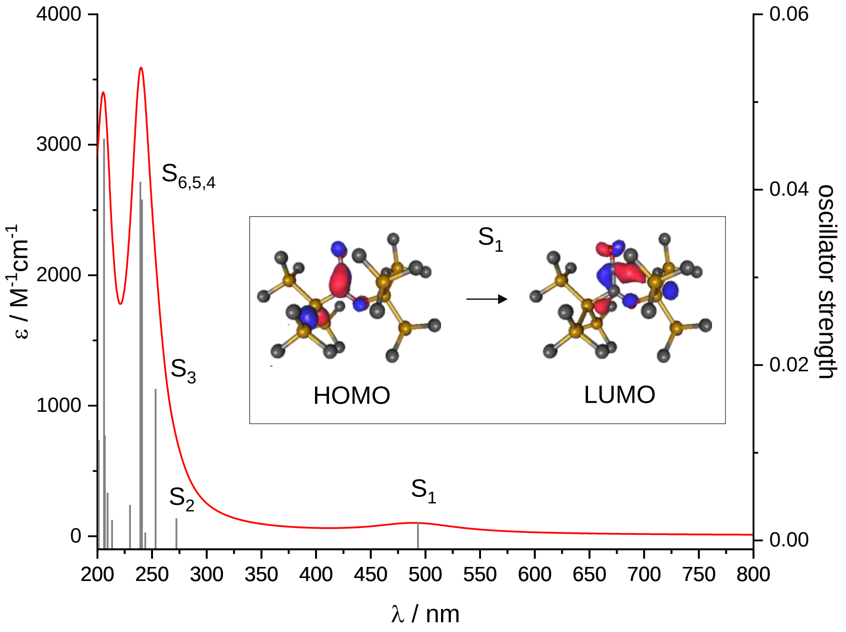

Figure S21. Calculated PBEh-3c UV/Vis spectrum of **4**. The vertical lines represent the oscillator strength of the individual bands, relevant orbitals are presented as an insert.

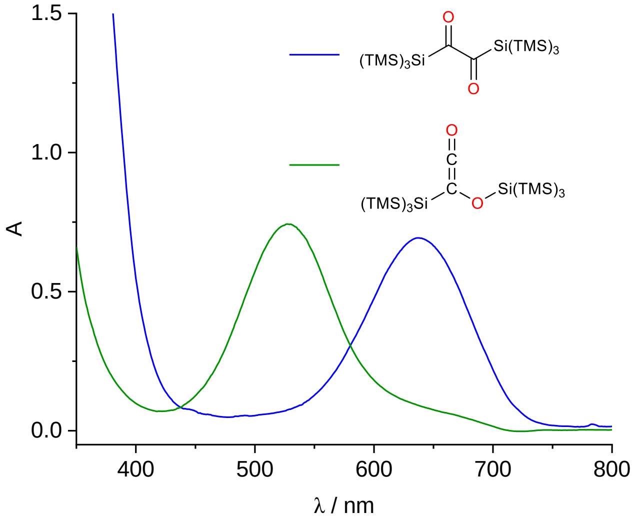

Figure S22. The overlap of **3** and **4** UV/Vis spectra

Irradiation of 4 with light sources having an emission maxima 590 / 630 nm

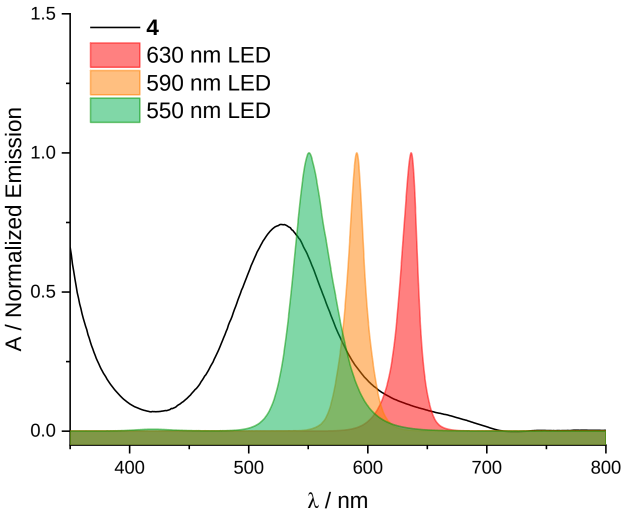

Figure S23. UV/Vis spectra of **4** overlapped with the normalized emission spectra of LED with an emission maximum at 550, 590, and 630 nm.

NMR spectra of 3

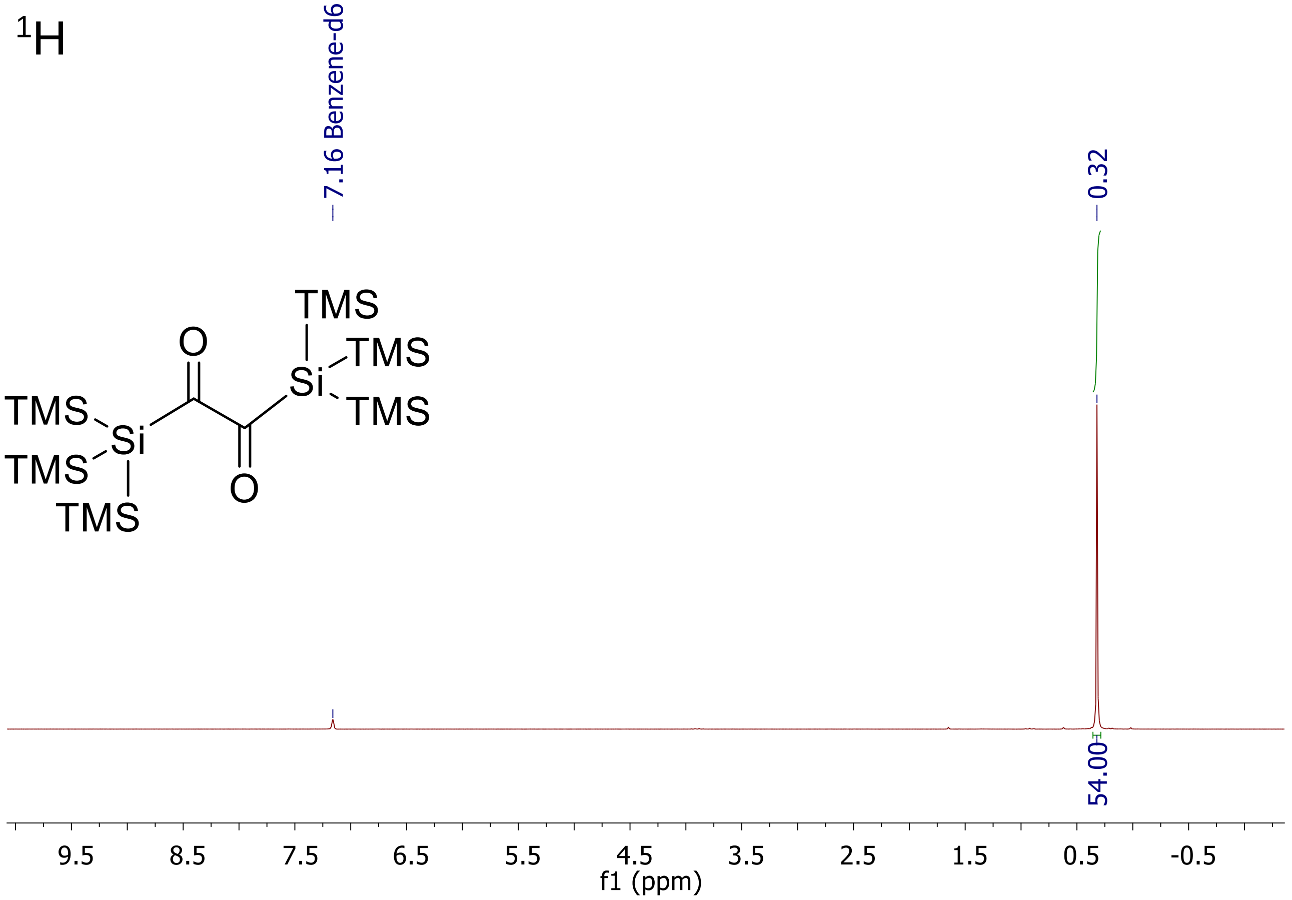

Figure S24. ^{1}H NMR spectrum of **3** (benzene-d_6 solution, vs ext. tetramethylsilane, ppm)

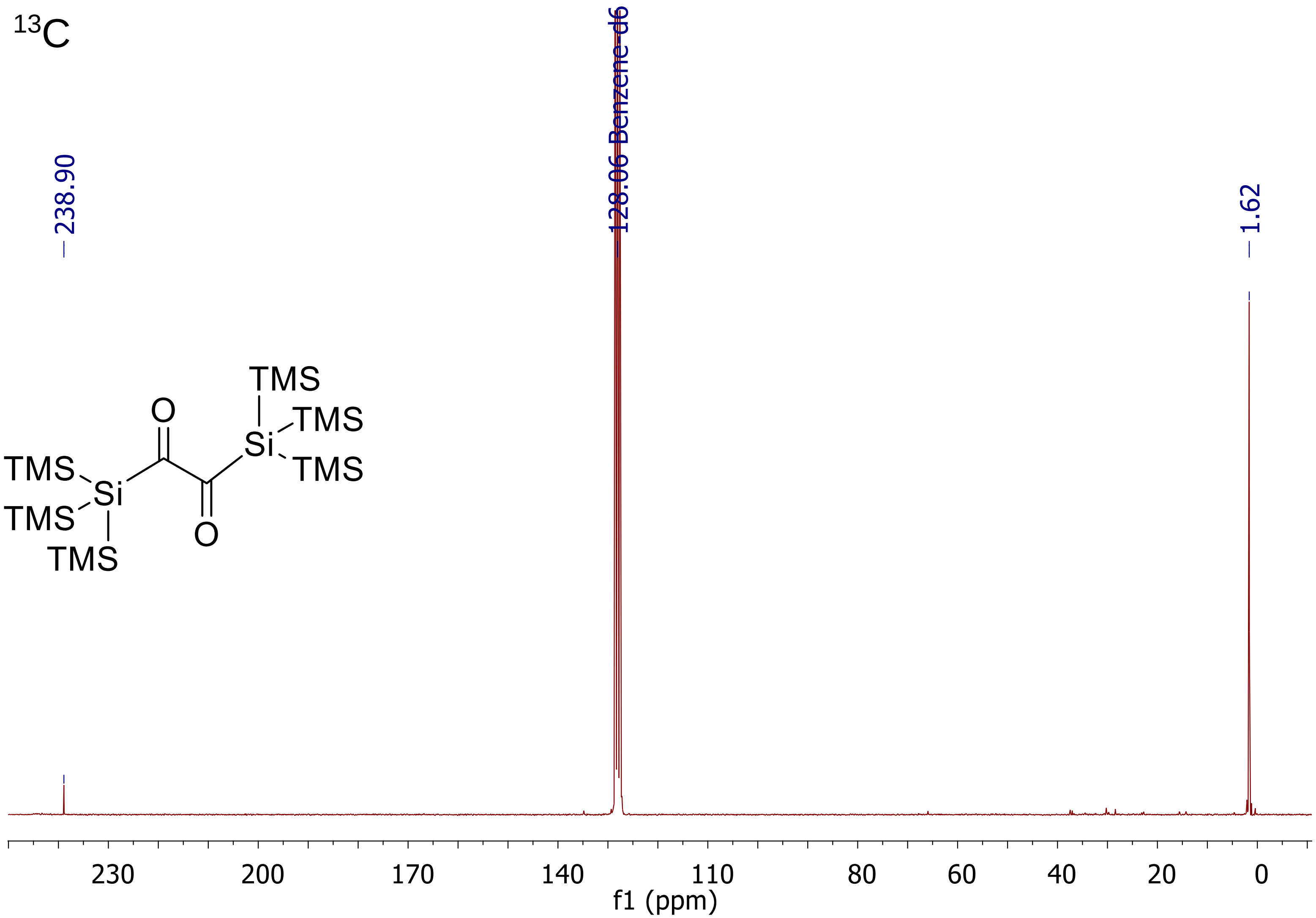

Figure S25. ^{13}C NMR spectrum of **3** (benzene-d_6 solution, vs ext. tetramethylsilane, ppm)

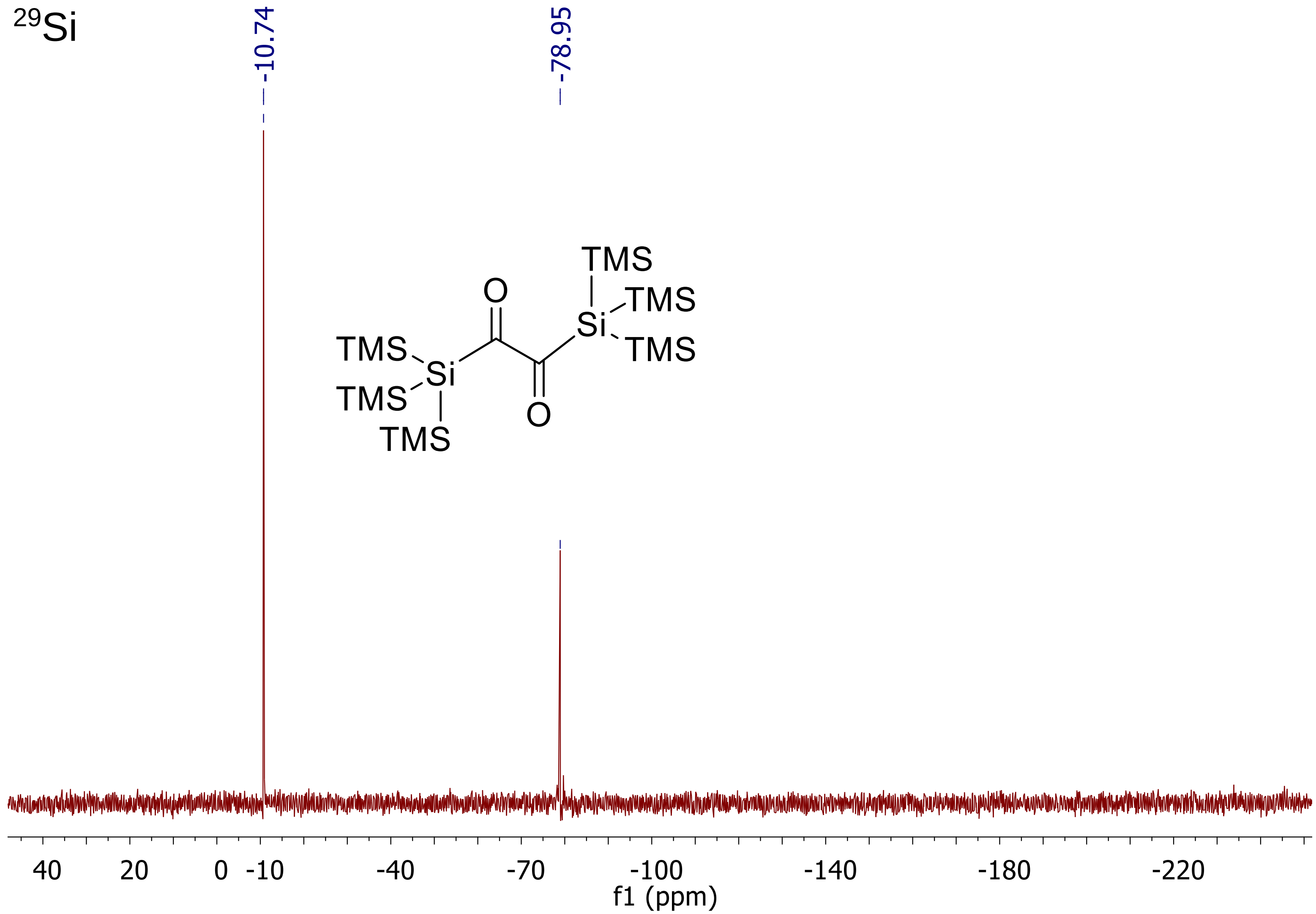

Figure S26. ^{29}Si NMR spectrum of **3** (benzene-d_6 solution, vs ext. tetramethylsilane, ppm)

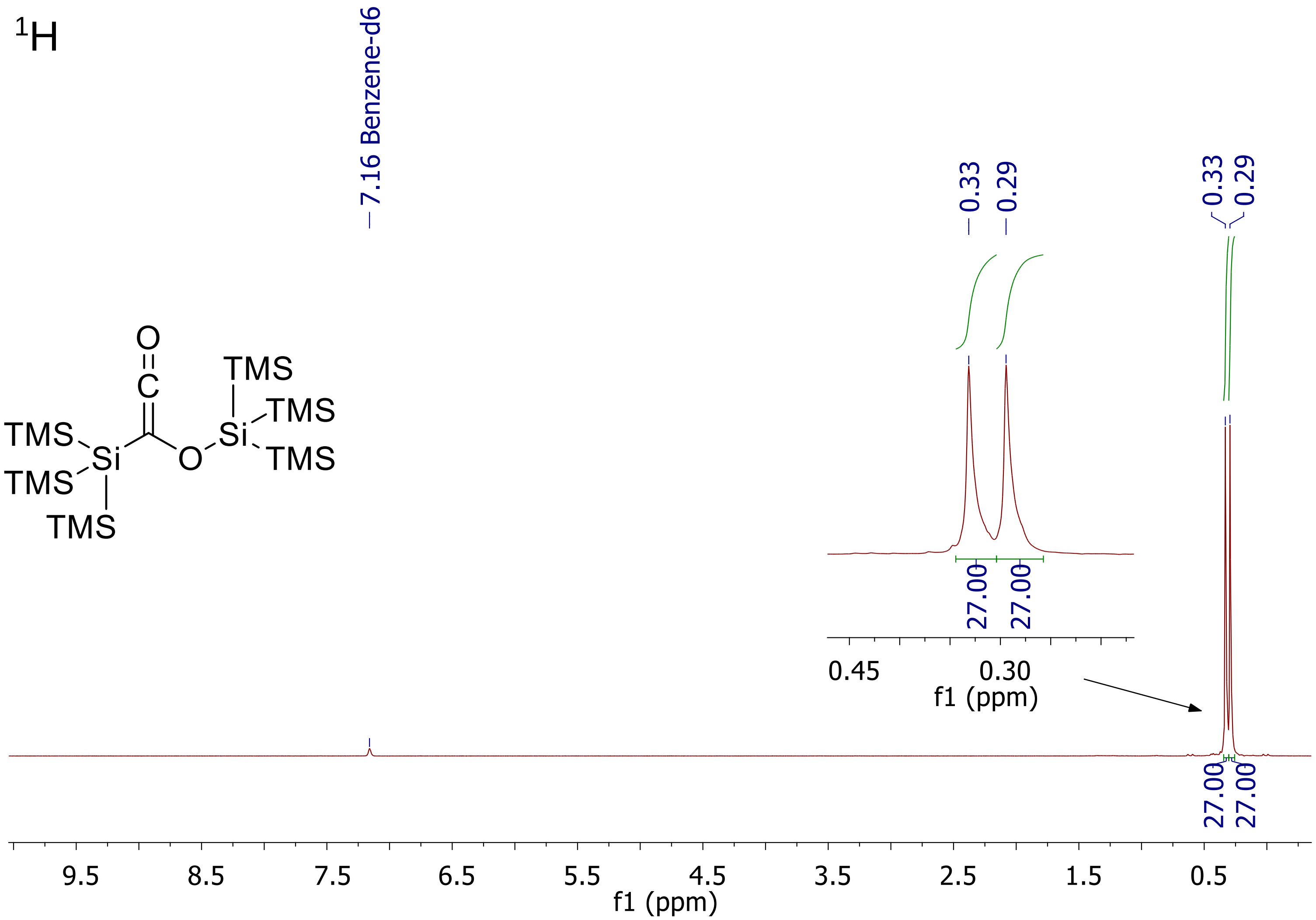

Figure S27. ^{1}H NMR spectrum of **4** (benzene-d_6 solution, vs ext. tetramethylsilane, ppm)

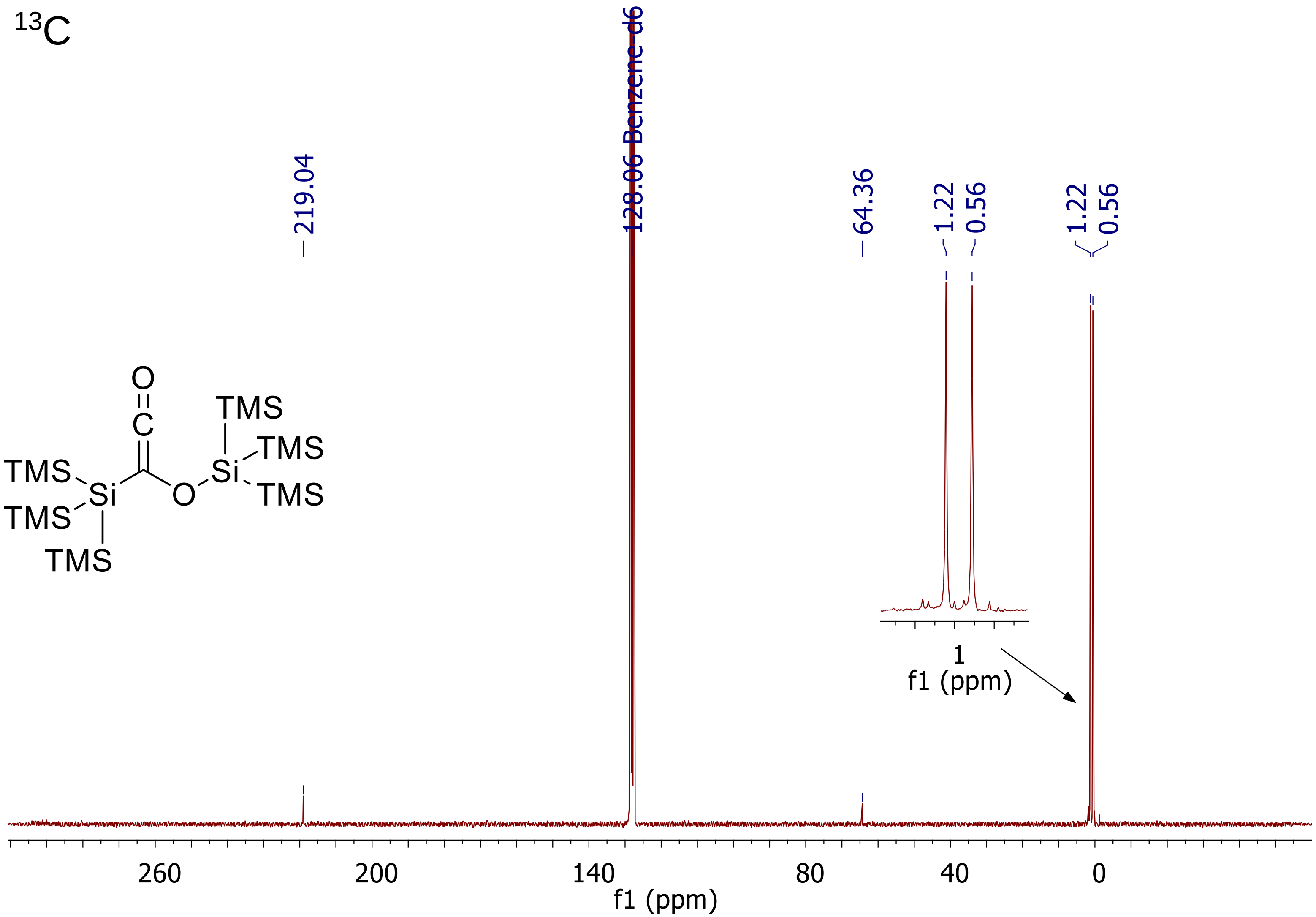

Figure S28. ^{13}C NMR spectrum of **4** (benzene-d_6 solution, vs ext. tetramethylsilane, ppm)

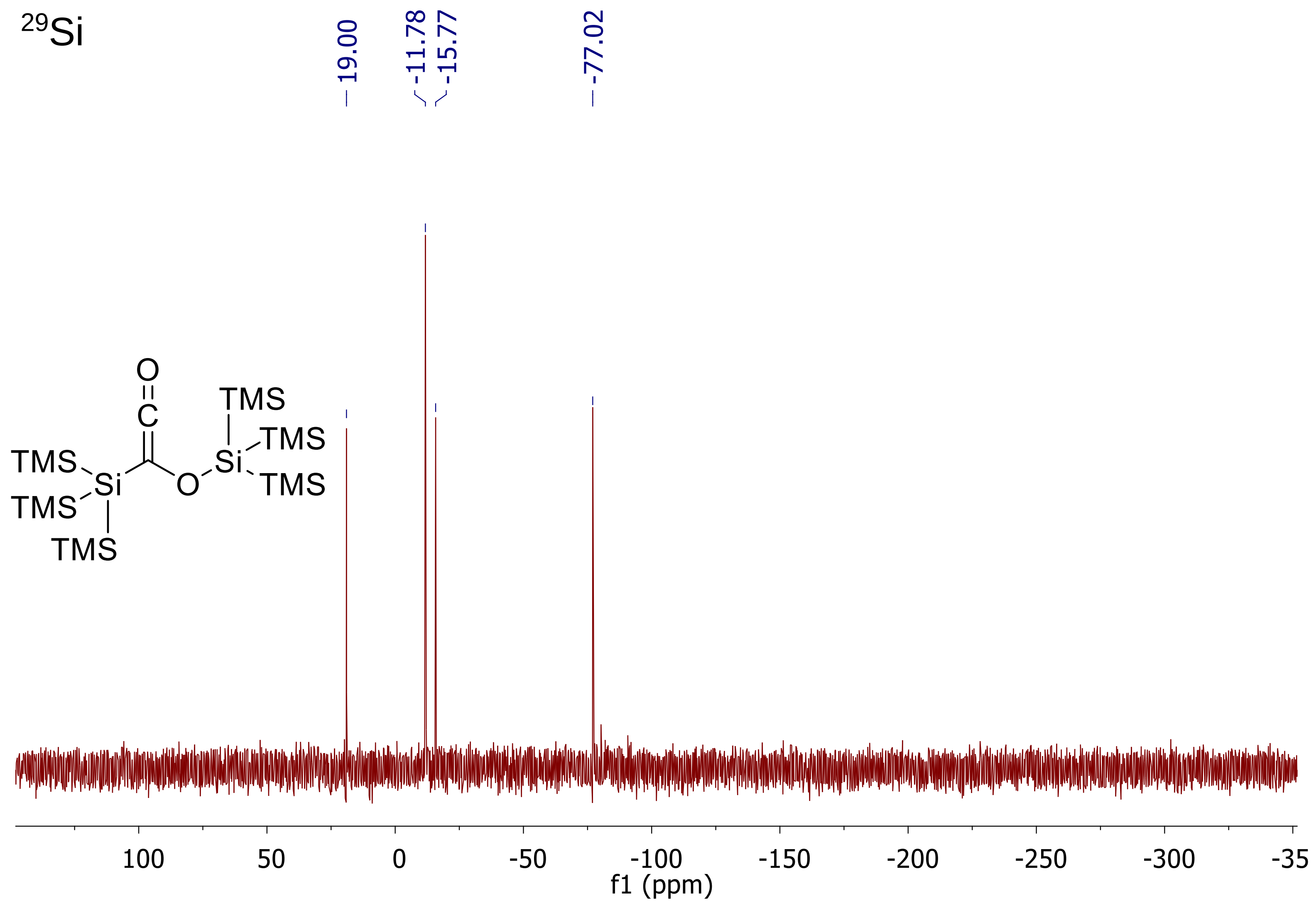

Figure S29. ^{29}Si NMR spectrum of **4** (benzene-d_6 solution, vs ext. tetramethylsilane, ppm)

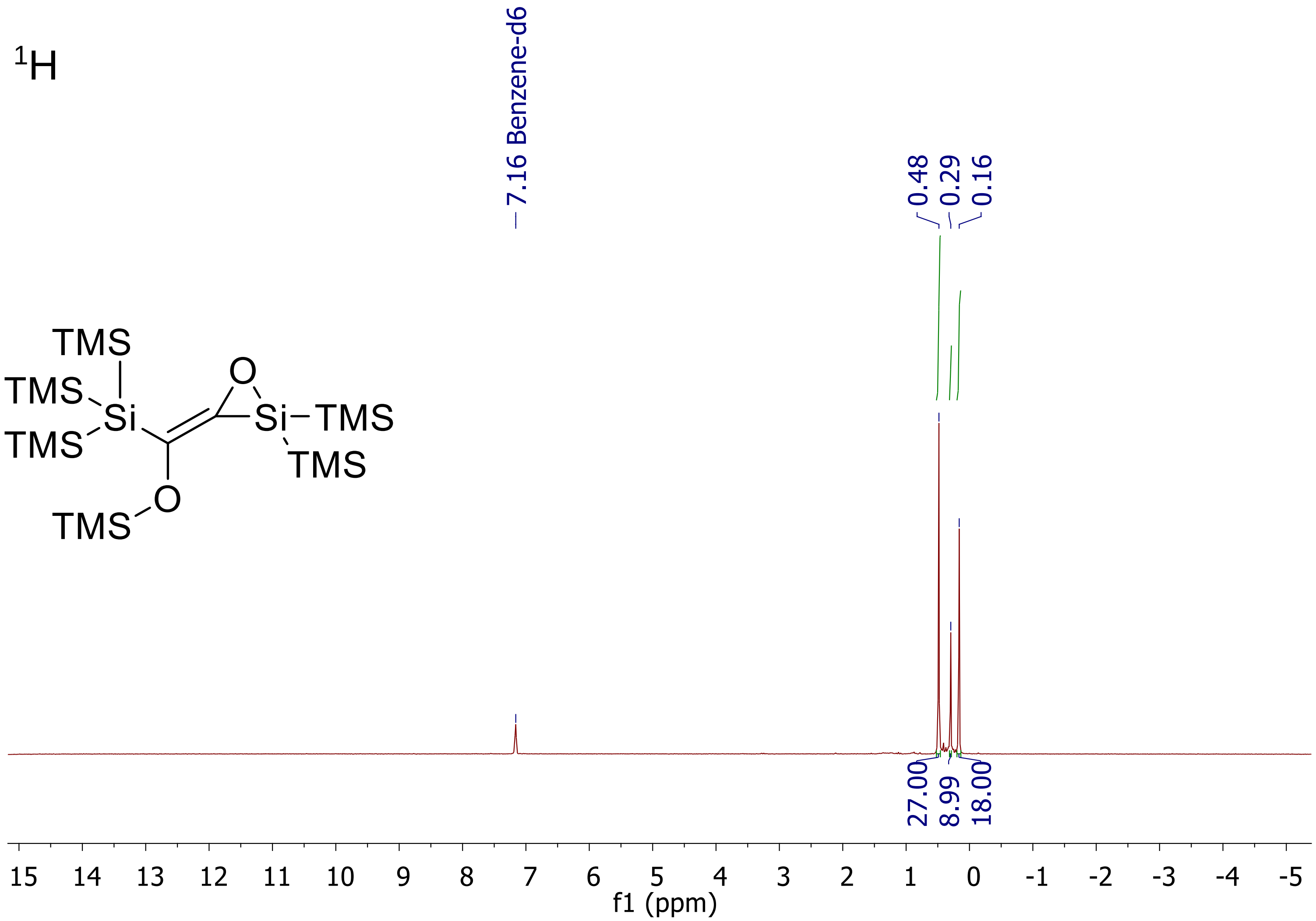

Figure S30. ^{1}H NMR spectrum of **5** (benzene-d_6 solution, vs ext. tetramethylsilane, ppm)

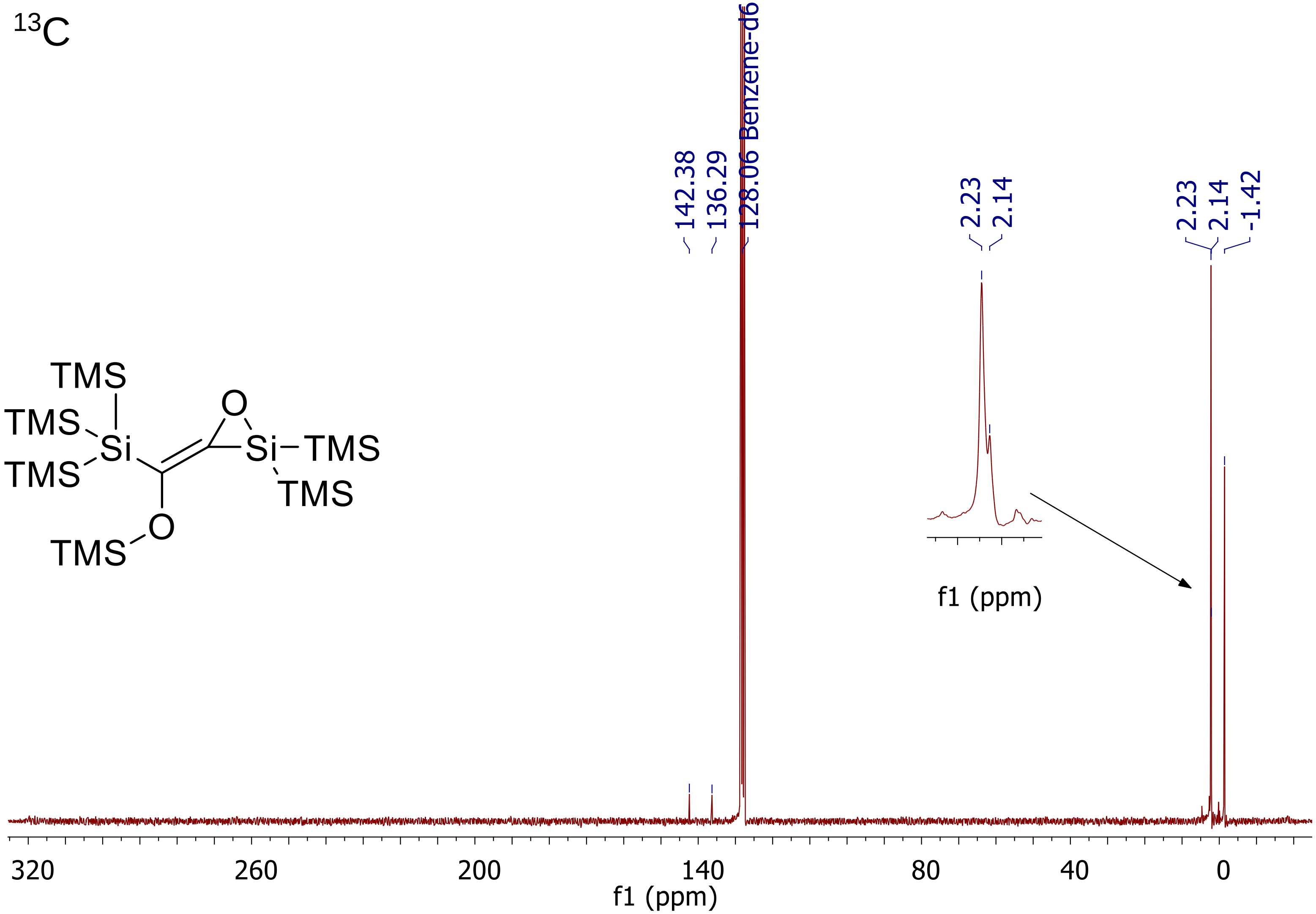

Figure S31. ^{13}C NMR spectrum of **5** (benzene-d_6 solution, vs ext. tetramethylsilane, ppm)

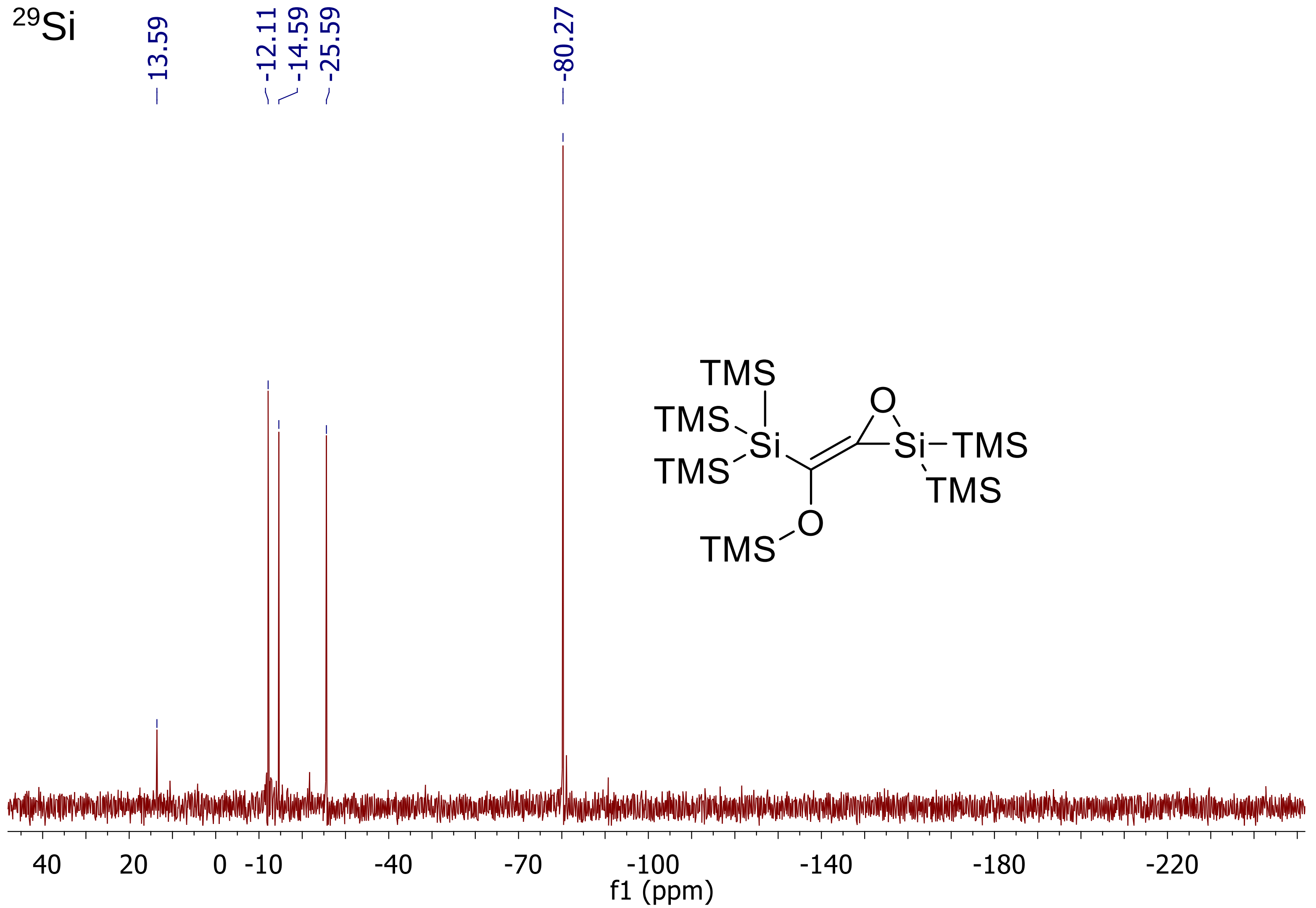

Figure S32. ^{29}Si NMR spectrum of **5** (benzene-d_6 solution, vs ext. tetramethylsilane, ppm)

NMR spectra of 6

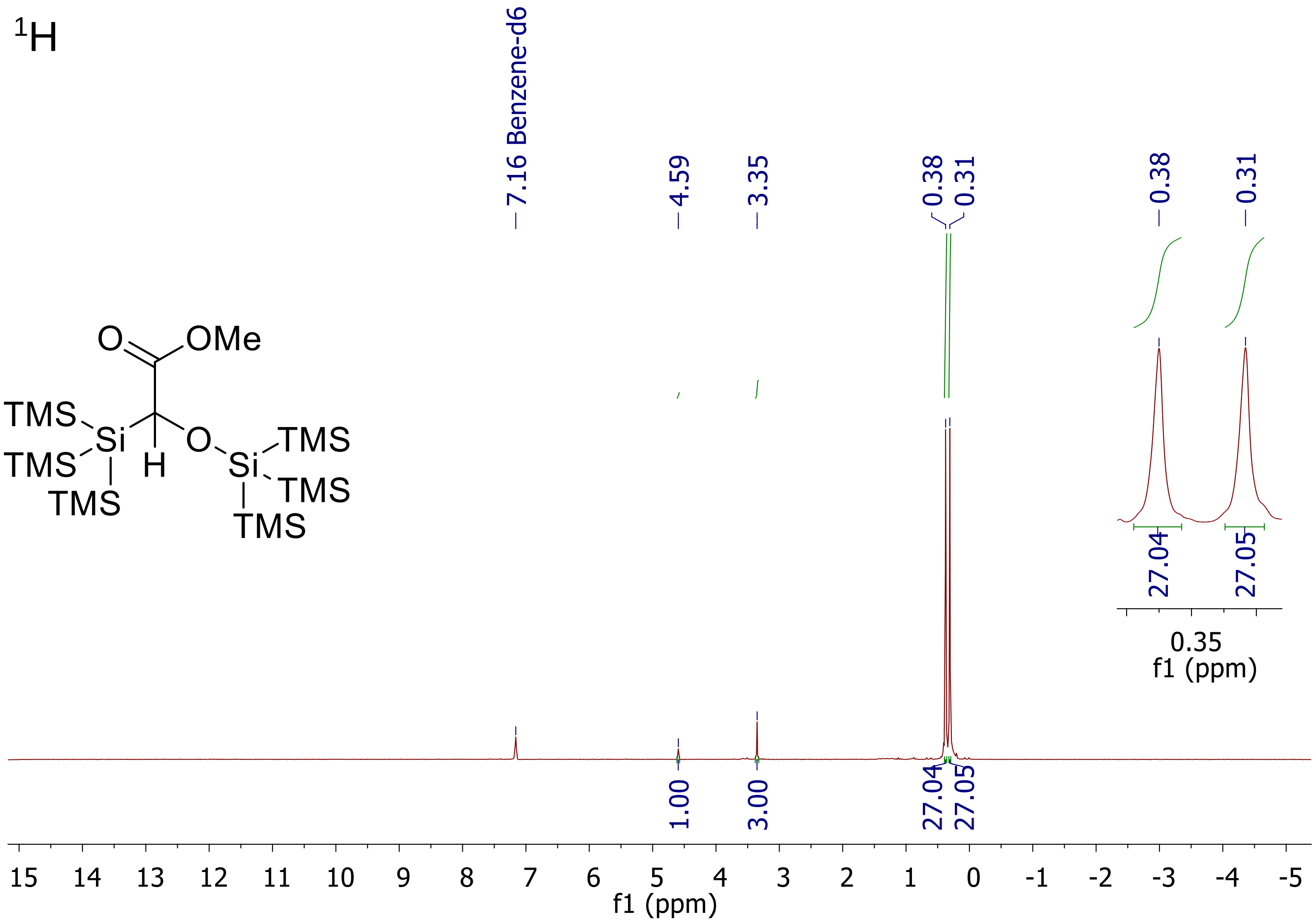

Figure S33. ^{1}H NMR spectrum of **6** (benzene-d_6 solution, vs ext. tetramethylsilane, ppm)

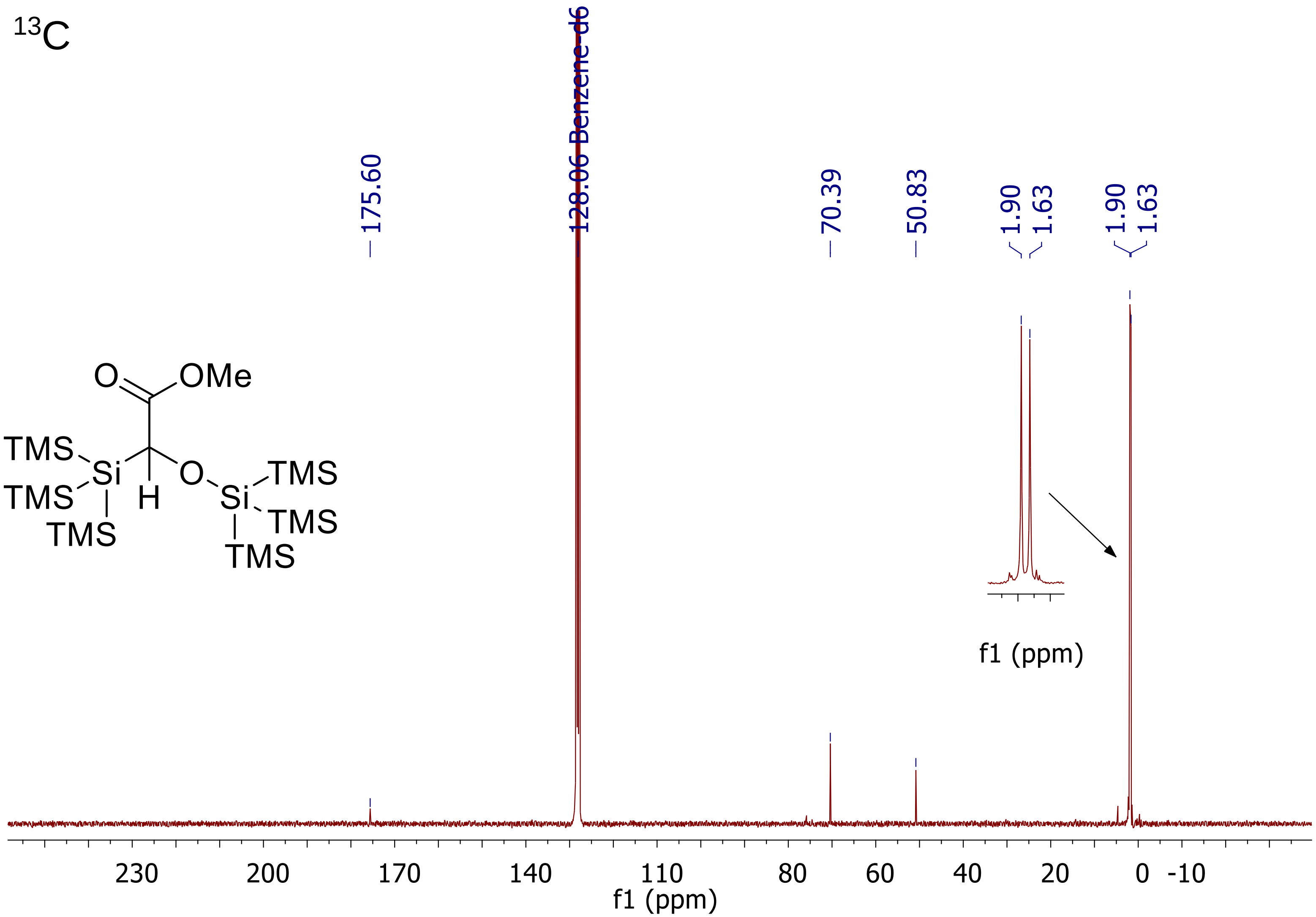

Figure S34. ^{13}C NMR spectrum of **6** (benzene-d_6 solution, vs ext. tetramethylsilane, ppm)

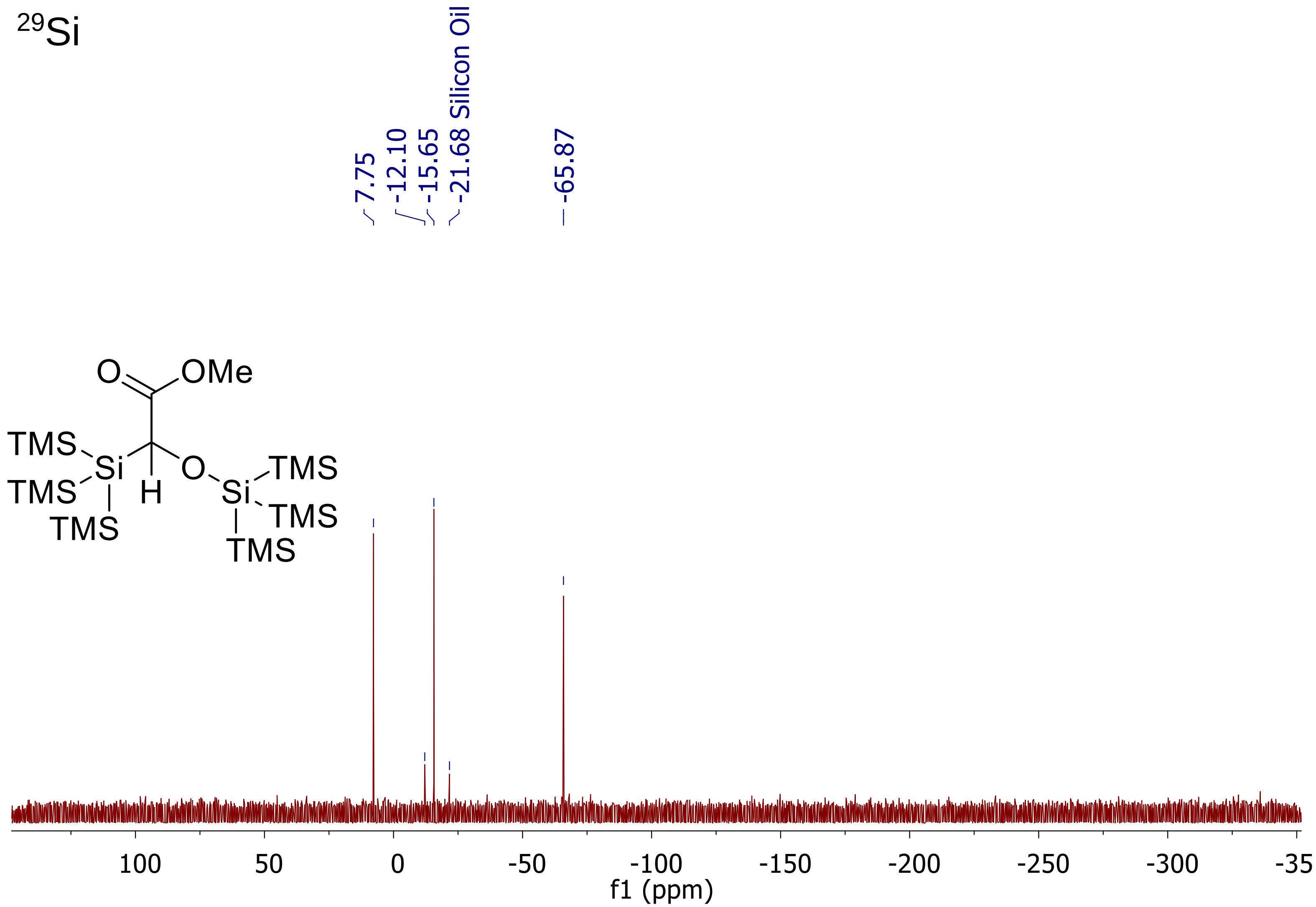

Figure S35. ^{29}Si NMR spectrum of **6** (benzene-d_6 solution, vs ext. tetramethylsilane, ppm)

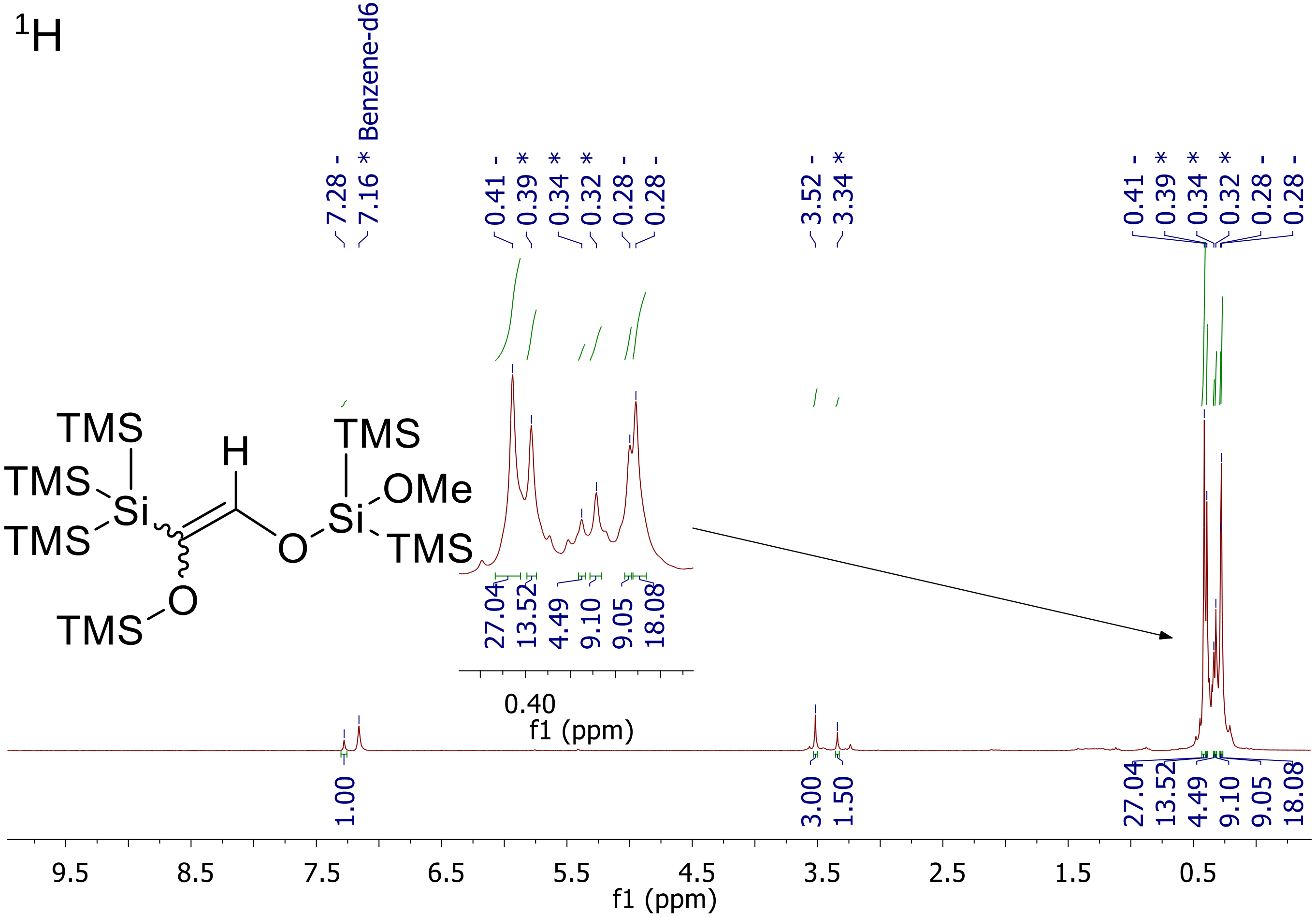

Figure S36. The crude ^{1}H NMR spectra for the product formation **7** after the irradiation of **3** in the presence of MeOH and Et_3N with LED having emission maxima at 590 nm (benzene-d_6 solution, vs ext. tetramethylsilane, ppm). Peaks are marked with – and *, respectively to differentiate between both formed isomers. Integration of the peak areas reveals a 2:1 ration of the isomers.

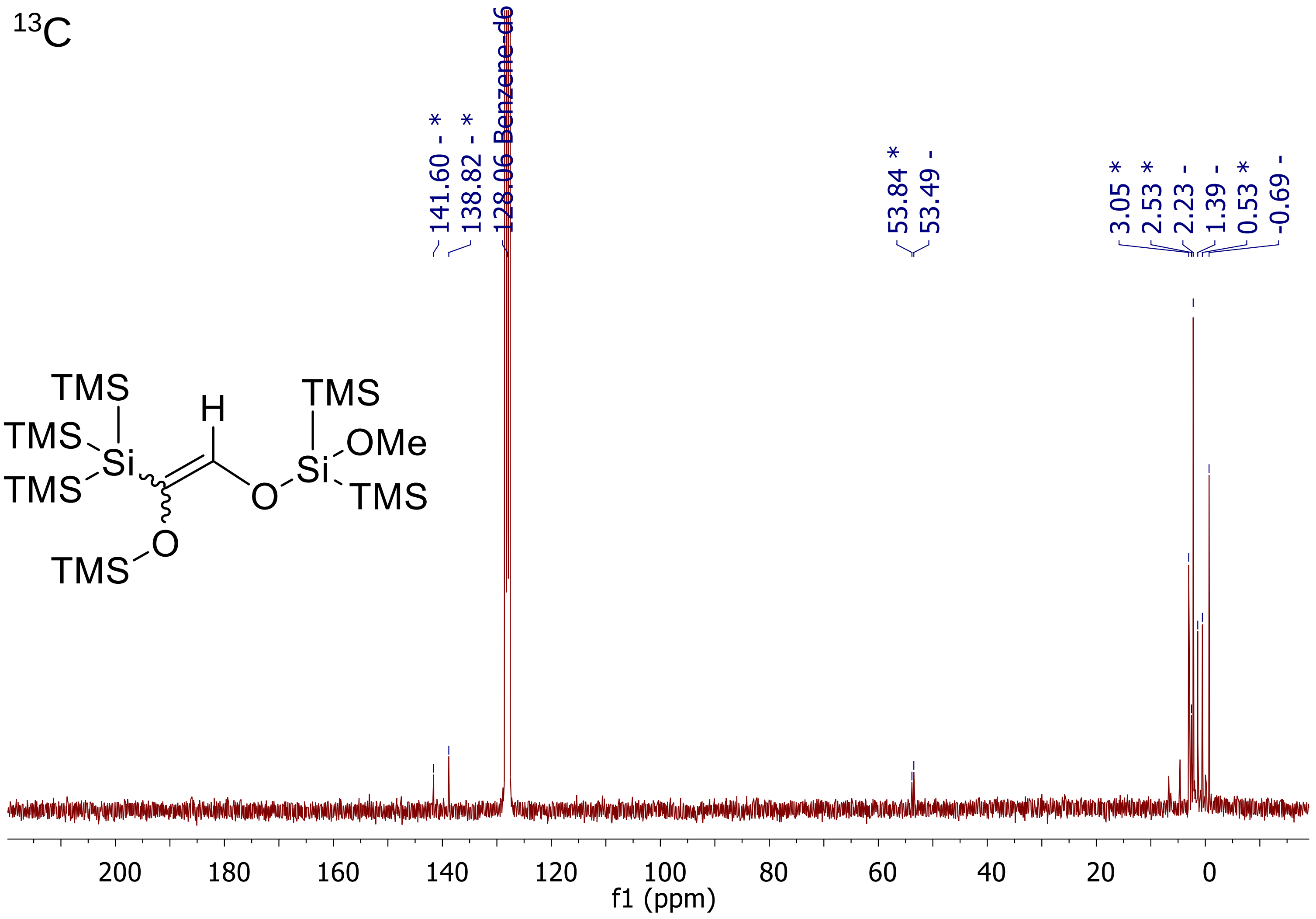

Figure S37. The crude ^{13}C NMR spectra for the product formation **7** after the irradiation of **3** in the presence of MeOH and Et_3N with LED having emission maxima at 590 nm (benzene-d_6 solution, vs ext. tetramethylsilane, ppm). Peaks are marked with – and *, respectively to differentiate between both formed isomers.

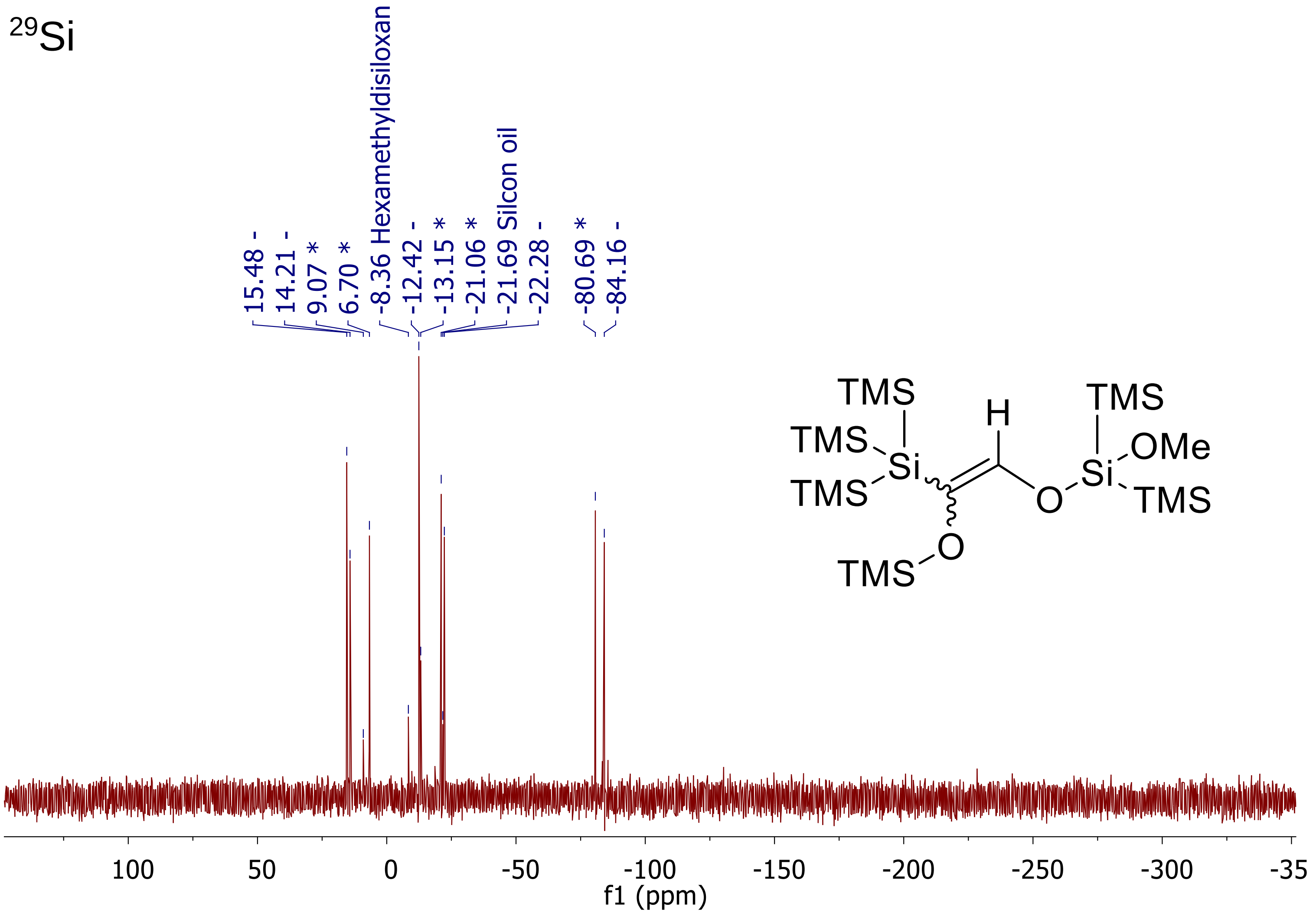

Figure S38. The crude ^{29}Si NMR spectra for the product formation **7** after the irradiation of **3** in the presence of MeOH and Et_3N with LED having emission maxima at 590 nm (benzene-d_6 solution, vs ext. tetramethylsilane, ppm). Peaks are marked with – and *, respectively to differentiate between both formed isomers.

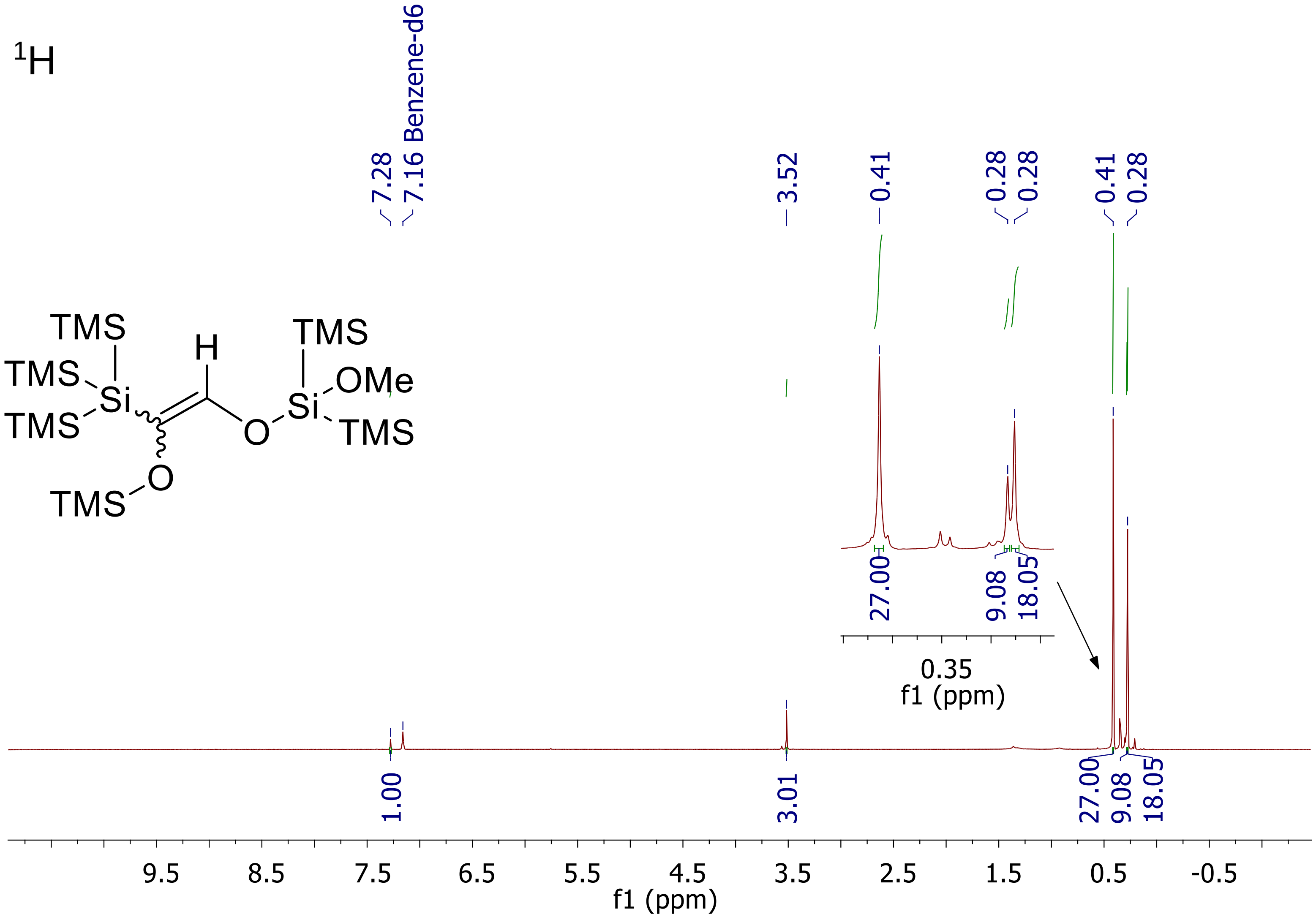

Figure S39. ^{1}H NMR spectrum of *isomer 2* of **7** (benzene-d_6 solution, vs ext. tetramethylsilane, ppm)

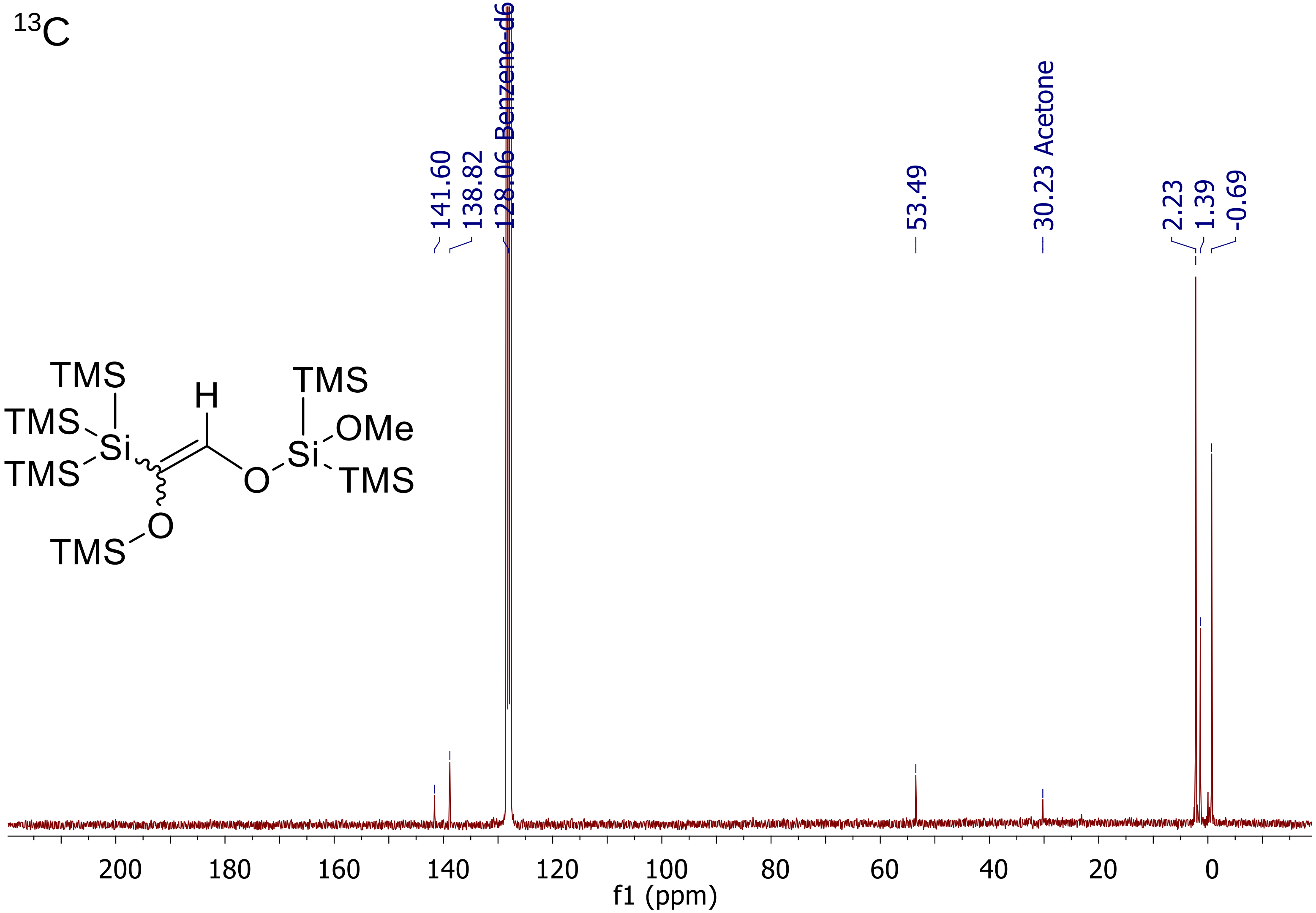

Figure S40. ^{13}C NMR spectrum of *isomer 2* of **7** (benzene-d_6 solution, vs ext. tetramethylsilane, ppm)

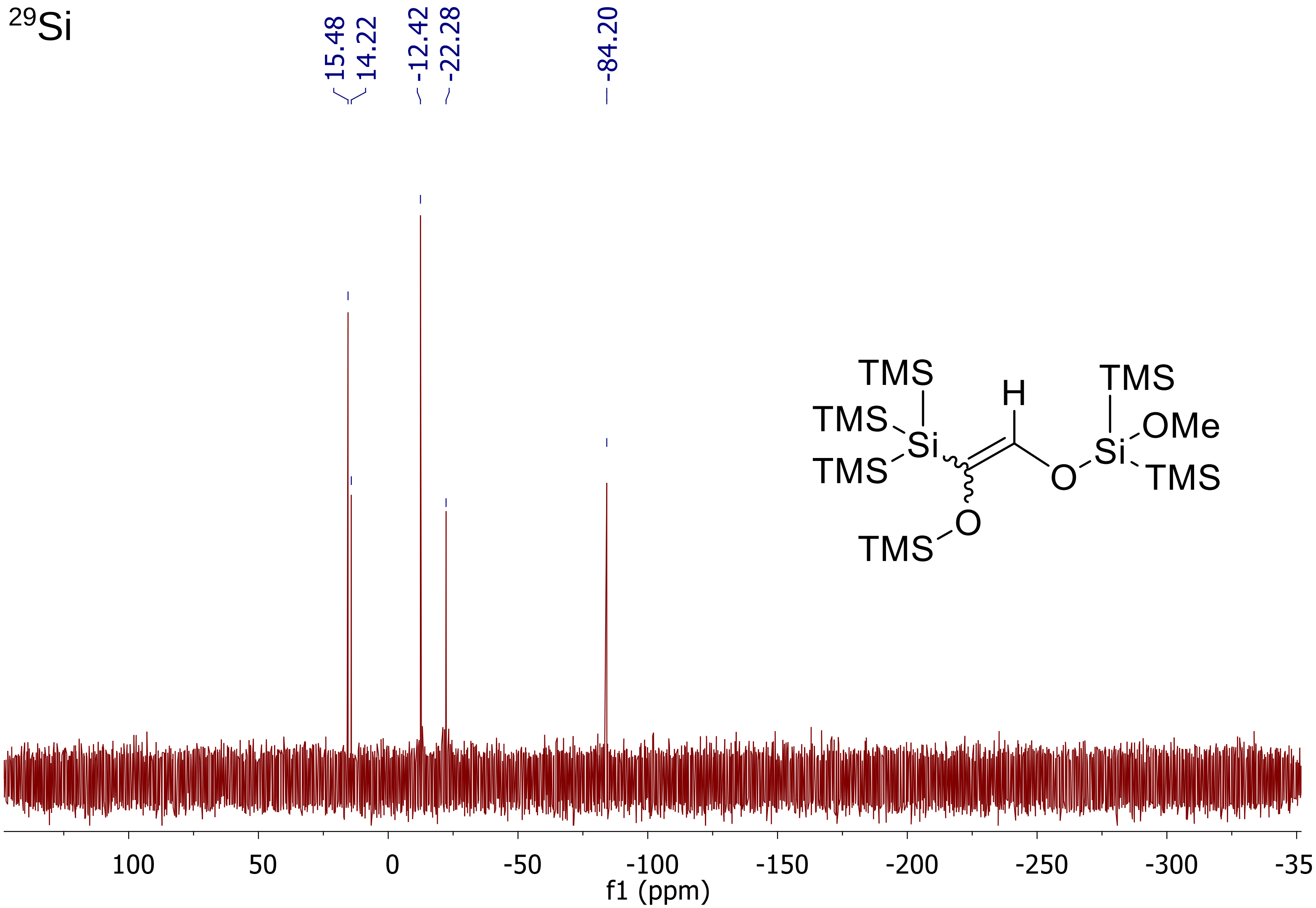

Figure S41. ^{29}Si NMR spectrum of *isomer 2* of **7** (benzene-d_6 solution, vs ext. tetramethylsilane, ppm)

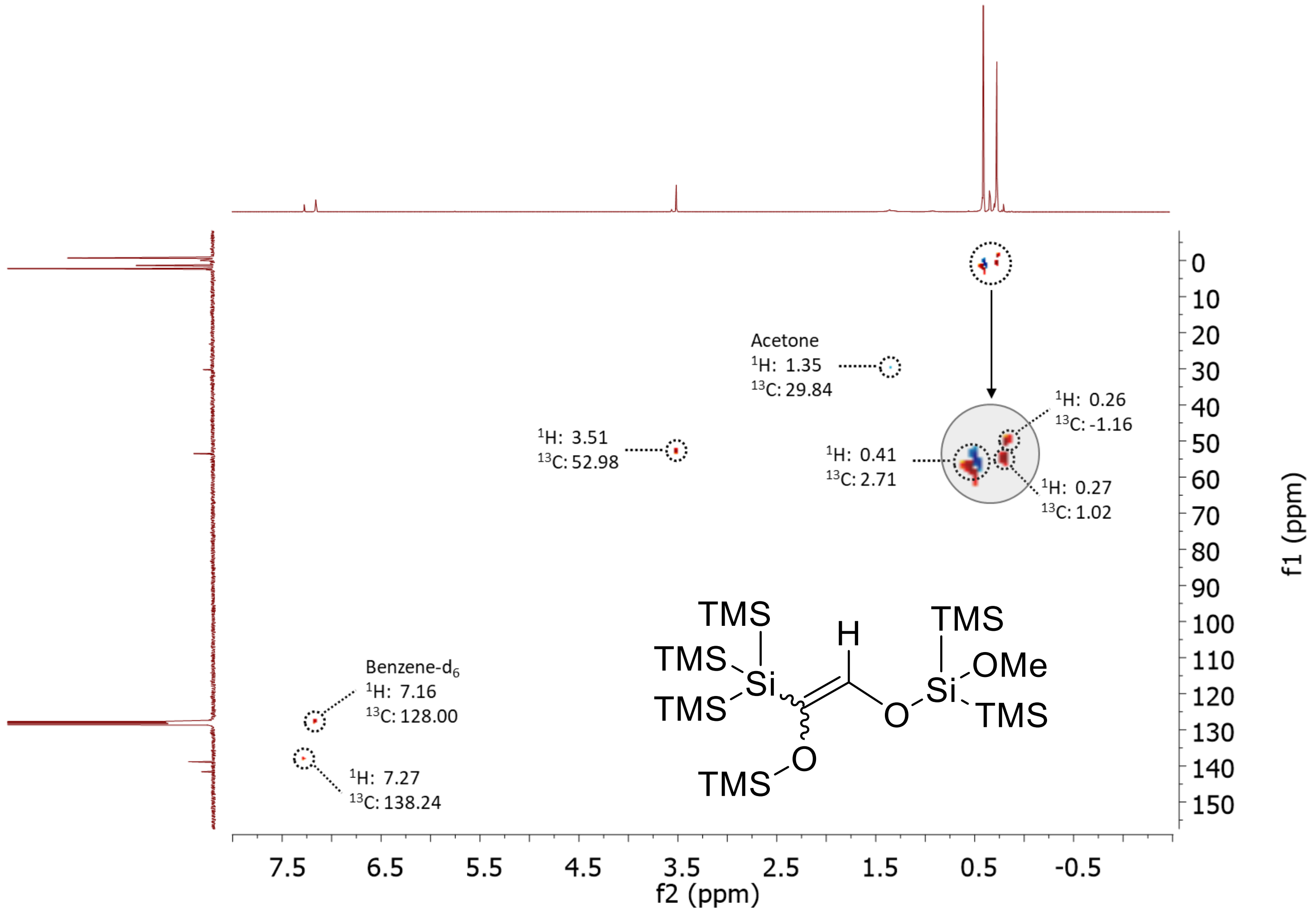

Figure S42. HSQC-NMR spectrum of *isomer 2* of **7** (benzene-d_6 solution, vs ext. tetramethylsilane, ppm)

X-Ray Crystallography

All crystals suitable for single-crystal X-ray diffractometry were removed from a vial or Schlenk flask and immediately covered with a layer of silicone oil. A single crystal was selected, mounted on a glass rod on a copper pin, and placed in a cold N_2 stream. XRD data collection for compounds **3** and **4** was performed on a Bruker APEX II diffractometer with the use of an Incoatec microfocus sealed tube of Mo Kα radiation (λ = 0.71073 Å) and a CCD area detector. Empirical absorption corrections were applied using SADABS or TWINABS.[20] The structures were solved with either the use of direct methods or the intrinsic phasing option in SHELXT and refined by the full-matrix least-squares procedures in SHELXL[21] or Olex2.[22] The space group assignments and structural solutions were evaluated using PLATON.[23] Nonhydrogen atoms were refined anisotropically. Hydrogen atoms were either located in a difference map or in calculated positions corresponding to the standard bond lengths and angles. The disorder was handled by modeling the occupancies of the individual orientations using free variables to refine the respective occupancy of the affected fragments (PART).[24] Table S1 in the Supporting Information contains crystallographic data and details of measurements and refinement for all compounds. Crystallographic data (excluding structure factors) have been deposited with the Cambridge Crystallographic Data Centre (CCDC) under the following numbers (**3**, 2242020, **4**, 2242021).

Crystallographic Table

Table S1. Crystallographic data and details of measurements for compounds **3**, and **4**. Mo Kα (λ=0.71073Å). $R1= \Sigma/ |F_o|- |F_c|/|\Sigma|F_d$; $wR2 = [\Sigma_w(F_o^2-F_2^2)^2/\Sigma_w(F_o^2)^2]^{1/2}$

Compound	**3** (2242020)	**4** (2242021)
Formula	$C_{10}H_{27}OSi_4$	$C_{20}H_{54}O_2Si_8$
Fw (g mol^{-1})	275.67	551.35
a (Å)	9.377 (5)	9.2895 (6)
b (Å)	9.613 (7)	9.5681
c (Å)	11.879 (7)	12.3723 (7)
α (°)	70.53 (2)	93.065 (3)
β (°)	71.722 (15)	108.756 (3)
γ (°)	61.483 (15)	118.620 (3)
V (Å^3)	871.5 (9)	885.12
Z	2	1
Crystal size (mm)	0.05 × 0.11 × 0.14	0.16 × 0.13 0× 0.04
Crystal habit	Block, blue	Plate, purple
Crystal system	Triclinic	Triclinic
Space group	*P*-1	*P*1
d_{calc} (Mg m^{-3})	1.050	1.034
µ (mm^{-1})	0.322	0.317
T (K)	99.68	99.8
2*ϑ* range (°)	1.851–26.997	2.504–25.999
F(000)	302	302
T_{min}, T_{max}	0.5153, 0.7456	0.5270, 0.7456
R_{int}	0.0680	0.0482
No. of measured, independent and observed [$I > 2\sigma(I)$] reflections	34383	34665
independent reflections	3785	6621
No. of parameters, restraints	0	312
Δρmax, Δρmin(e Å^{-3})	1.541, -0.484	0.81, -0.53
R1, wR2 (all data)	R1 = 0.1075<br>wR2 = 0.2205	R1 = 0.0803<br>wR2 = 0.1631
R1, wR2 (>2*σ*)	R1 = 0.0727<br>wR2 = 0.1988	R1 = 0.0592<br>wR2 = 0.1456

Calculations

After optimization of compound **3** at the DFT PBEh-3c level, we can characterize the relevant frontier orbitals (Table S2). The HOMO and HOMO-5 orbitals are the symmetric and asymmetric linear combinations of the non-bonding n-orbitals on both C=O carbonyl group's oxygen atoms with additional contributions of the neighboring C-Si bond. The orbitals HOMO-4 til HOMO-1 are localized at the Si-Si_{TMS} sigma bonds with different orientation towards one of the TMS groups. The LUMO orbital is the positive linear combination of both carbonyl π* orbitals, and their negative linear combination forms the LUMO+2, where in the latter also a large σ* contribution at the neighboring Si atoms contributes. The LUMO+1 orbital is a σ* orbital of the Si atoms with a minor contribution on the carbonyl C=O groups.

Table S2. Relevant orbitals of **3** as computed with the PBEh-3c method. Contour values of 0.05 a.u. were selected in the program *gabedit* for the orbital pictures.

	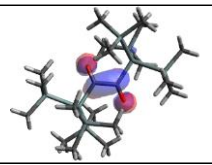	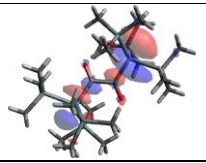	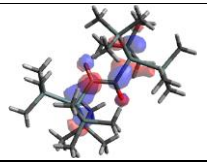		
	LUMO	LUMO+1	LUMO+2		
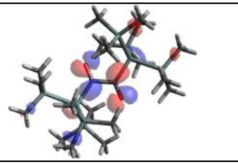	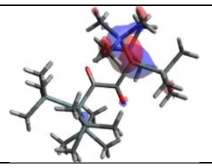	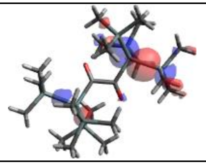	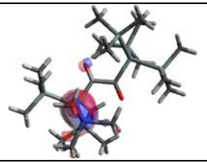	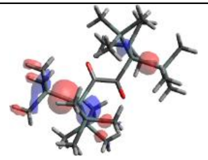	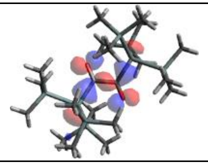
HOMO-5	HOMO-4	HOMO-3	HOMO-2	HOMO-1	HOMO

Although a biradicalic state cannot be properly described with DFT without applying a multireference method, TDDFT is suitable to calculate the vertical transitions from the ground state of **3** in agreement with the experimental data. The PBEh-3c method predicts vertical excitations with a small systematic overestimation of the energies, therefore, the vertical excitations shown in Fig.2 of the main manuscript were corrected by -1500 cm^{-1}, whereas the original computed data are presented in Table **S3** and Figure S43. We have assigned the lowest eight transitions, which form the first three bands in the experimental spectrum (see Figure **S1d** for the simulation of the UV spectrum).

The first two computed TDDFT transitions (F_1 and F_2 in Table **S3**) are n/σ→λ* transitions from HOMO and HOMO-5 to the π* system of the dione moiety (LUMO). Both vertical excitations have a small oscillator strength, where the F_1 transition is relevant for the experimental peak at 650 nm. The F_2 transition arises at the long-wavelength side of the second experimental band at 317 nm, whose intensity is mainly formed by the various σ→π* transitions F_3-F_6 from HOMO-2 til HOMO-4 into the LUMO orbital. Transitions F_7 and F_8 are excitations into the Si σ* orbitals (linear combinations of LUMO+1 and LUMO+2), where the latter is mainly responsible for the intensity of the large experimental band at ca. 210 nm.

Table S3. Computed PBEh-3c TDDFT vertical excitations of compound **3** with their wavelengths, excitation energies, oscillator strength f and the respective orbital contribution (c^2 are listed for coefficients c > 0.25) with assignment of the main contribution. The assigned MOs are shown in Table **S2**.

Vertical transitions (F) (a)	λ/ nm	E / $kJmol^{-1}$	f	MO (c^2)	MO character
F_1	604.2	198.0	0.003	HOMO → LUMO (0.95)	$n_{sym,O} + \sigma_{Si\text{-}C\text{-}C\text{-}Si} \rightarrow \pi^*_{sym\ C=O}$
F_2	316.6	377.9	0.001	HOMO-5 → LUMO (0.53)<br>HOMO → LUMO+1 (0.08)<br>HOMO-17 → LUMO (0.08)	$n_{asym,O} + \sigma_{Si\text{-}C\text{-}C\text{-}Si} \rightarrow \pi^*_{sym\ C=O}$
F_3	313.1	382.1	0.007	HOMO-1 → LUMO (0.95)	$\sigma_{Si\text{-}C\text{-}C\text{-}Si} \rightarrow \pi^*_{sym\ C=O}$
F_4	297.1	402.6	0.003	HOMO-3 → LUMO (0.72), HOMO-4 → LUMO (0.15)	$\sigma_{Si,px} + \sigma_{Si,pyi} \rightarrow \pi^*_{sym\ C=O}$
F_5	289.9	412.7	0.048	HOMO-2 → LUMO (0.63), HOMO-4 → LUMO (0.33)	$\sigma_{Si,px} + \sigma_{Si,pyi} \rightarrow \pi^*_{sym\ C=O}$
F_6	288.6	414.5	0.066	HOMO-4 → LUMO (0.43) HOMO-2 → LUMO (0.27), HOMO-3 → LUMO (0.24)	$\sigma_{Si} + \sigma_{Si} \rightarrow \pi^*_{sym\ C=O}$
F_7	233.7	511.8	0.004	HOMO → LUMO+1 (0.48)<br>HOMO → LUMO+2 (0.26)<br>HOMO-5 → LUMO (0.18)	$n_{sym,O} + \sigma_{Si\text{-}C\text{-}C\text{-}Si} \rightarrow \sigma^*_{Si}$ $(+\pi^*_{asym\ C=O})$
F_8	208.7	573.3	0.448	HOMO → LUMO+2 (0.57)<br>HOMO → LUMO+1 (0.34)	$n_{sym,O} + \sigma_{Si\text{-}C\text{-}C\text{-}Si} \rightarrow \sigma^*_{Si} + \sigma^*_{Si@TMS} + \pi^*_{asym\ C=O}$

(a) The vertical excitations are named F1, F2, etc. in this work so that we do not mix it up with the naming of the optimized S1 and S2 states.

DFT Discussion added to Figure 10: The most important effect of silicon is that the Si σ orbitals on the silyl group are energetically raised and the respective σ* orbitals are lowered. We have tested this behavior on the small model molecule with R=SiH3, where those orbitals are then directly below the n orbitals (as HOMO-2) and above the dione π* orbital (LUMO+1) (see Table S4 and Figure S43) With the bulky $Si(SiMe_3)_3$ groups, this effect becomes even more prominent, and orbitals at the silyl group shift in-between the two n orbitals so that the Si σ orbital becomes the HOMO-1. Additionally, the LUMO+1 has a large contribution of Si σ* character. This leads to the fact that both n/σ–π* transitions are at the long-wavelength scale of molecule **3**, the same transitions for the biacetyl molecule.[25,26]

Table **S4**. Orbital energies (in eV) of various diones R-C(=O)-C(=O)-R, computed with the DFT PBEh-3c method, as depicted in Figure 10. The character of the orbitals is given in the header or the footnote. Energies of orbitals with n-character are printed in italic. The highest Si σ orbital is printed in the first column.

	Si σ	HOMO-1 (n_{asym})	HOMO (n_{sym})	LUMO π_{sym}*)	LUMO+1
R=H		*-10.84*	*-8.87*	-2.25	1.73
R=CH3		*-10.45*	*-8.20*	-1.63	1.96
R=SiH3	-11.06 (HOMO-2)	*-10.10*	*-7.92*	-2.36	0.37
R=Ph		-8.39 *(-10.24)* [(a)]	*-8.07*	-1.63	1.01
R=C(SiMe3)3		-8.50 *(-10.16)* [(b)]	-7.86	-1.43	1.53
R=Si(SiMe3)3	-7.49 (HOMO-1)	-7.49 *(-8.95)* [(c)]	-6.88	-1.74	0.67

(a) energy of the second n-orbital of R=Ph is the HOMO-5.

(b) energy of the second n-orbital of R=C(SiMe3)3 is the HOMO-17.

(c) energy of the second n-orbital of R=S(SiMe3)3 is the HOMO-5.

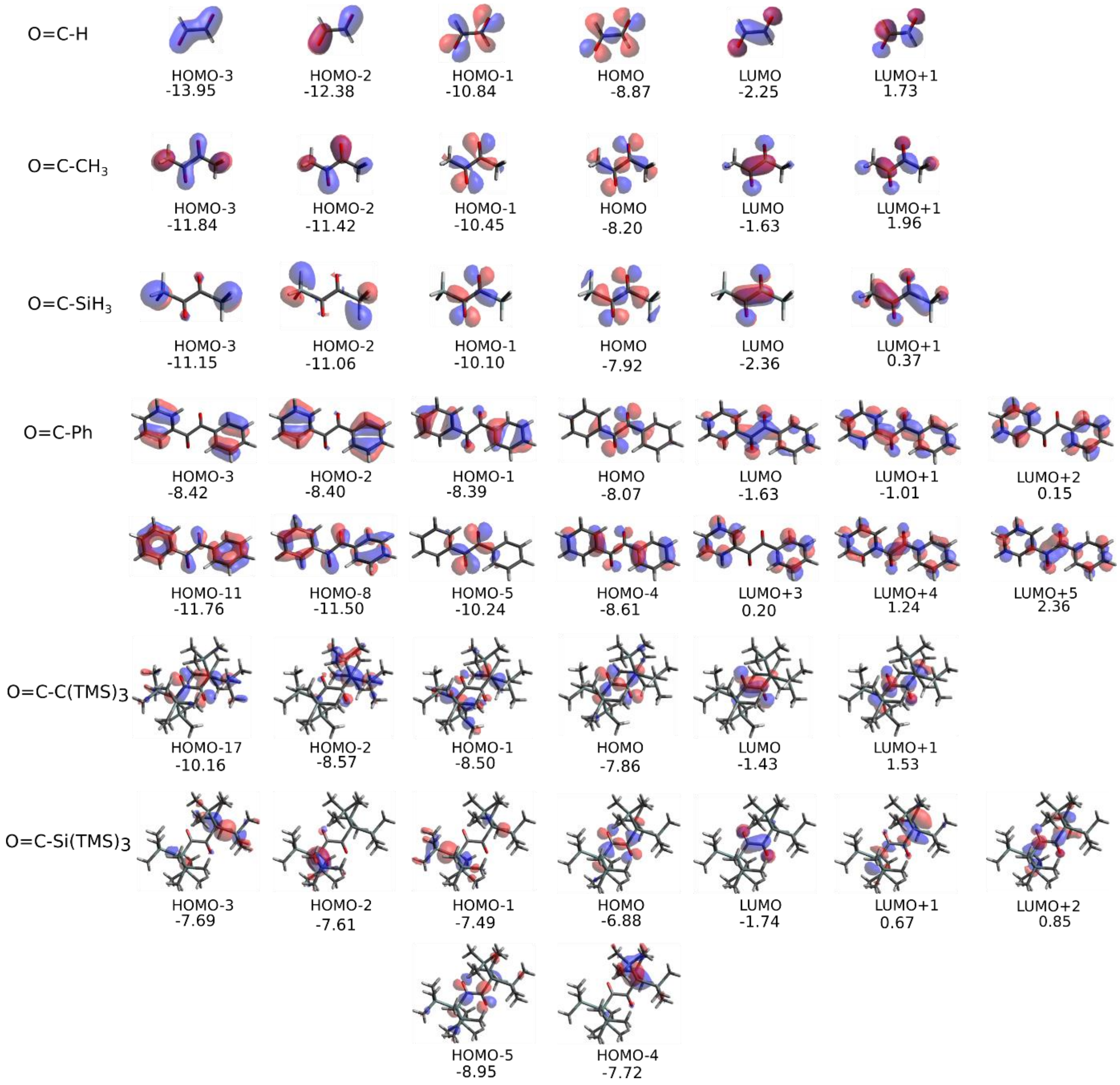

Figure S43 Orbitals of all molecules from Figure 10 with their respective orbital energies (in eV), computed with the PBEh-3c method. Contour values of 0.05 a.u. are selected in the program *gabedit* for the orbital pictures.

Computed IR Spectra for compound 3,4 and 5

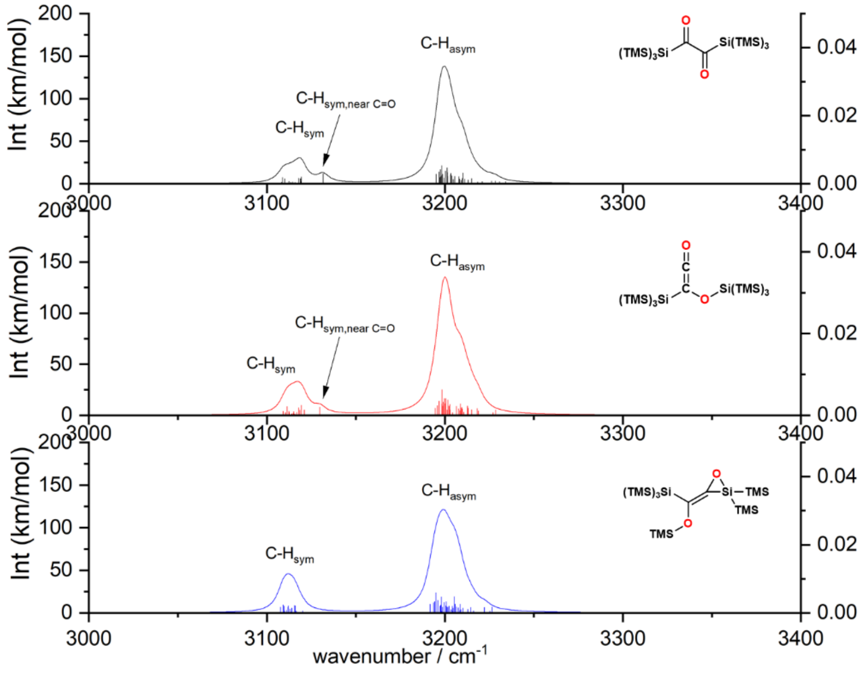

Figure S44. Unscaled computed IR spectra for compounds **3**, **4** and **5**. Region of C-H vibrations is shown with assigned vibrations.

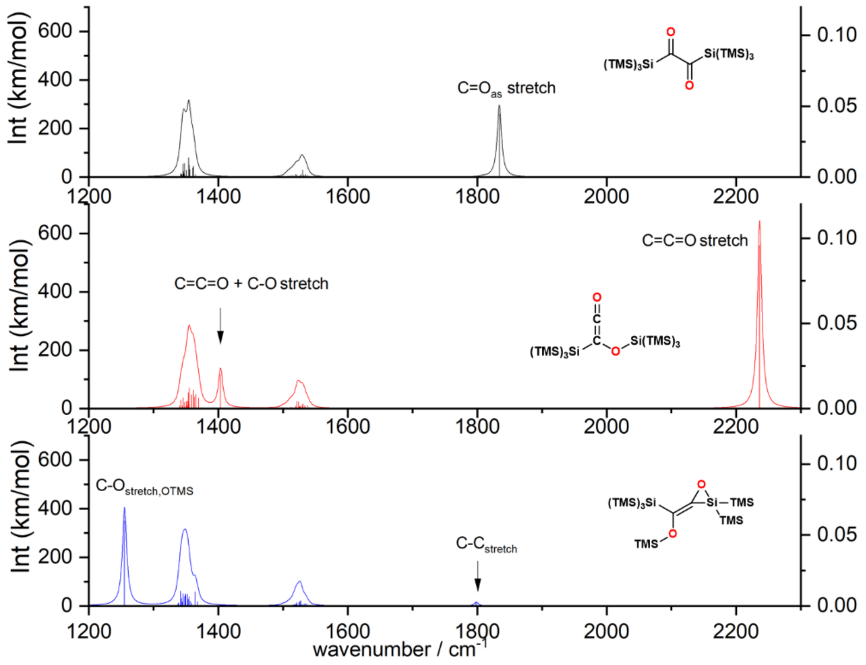

Figure S45. Unscaled computed IR spectra for compounds **3**, **4** and **5**. Region of C=O, and C-O vibrations is shown with assigned vibrations

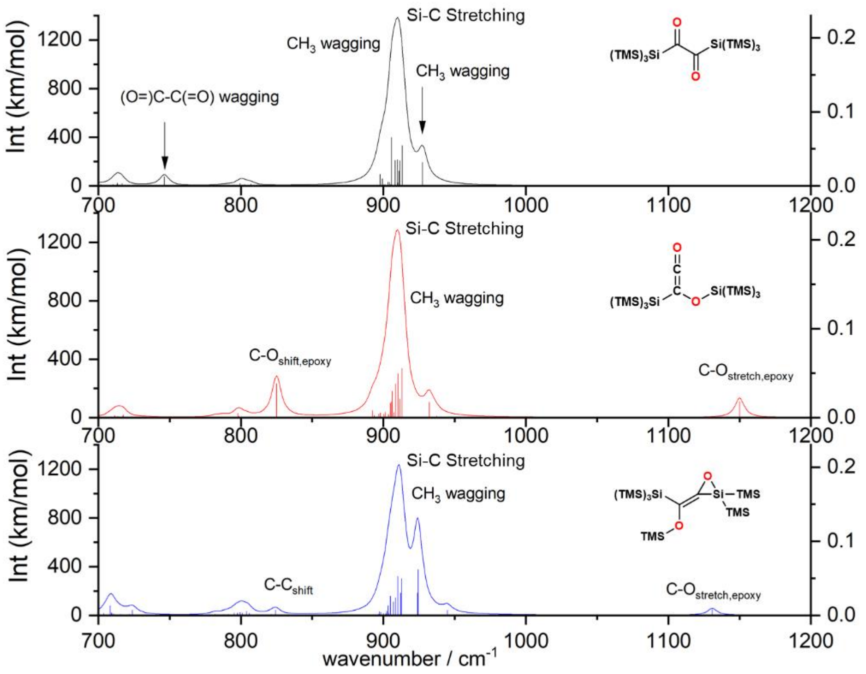

Figure S46. Unscaled computed IR spectra for compounds **3**, **4** and **5**. Region of low energy vibrations (Si-C stretching, CH_3 wagging) is shown with assigned vibrations.

Computed rearrangement mechanism of 3

Photochemical Pathway from 3→4:

DFT cannot locate biradicals, therefore, we applied the ADC(2) method to support the reaction mechanism of compound **3**→**4**. After optimization of the geometries in the S_0, S_1 and S_2 state, we checked the energetics (Figure S46), the geometries (Figure S48) and the natural transition orbitals (NTOs) (Figure S49).

After vertical excitation of **3** to the S_2 state (E_{rel} = 344 kJmol^{-1}) with 365 nm light, the molecule gains so much energy that it will performing large molecular vibrations that lead to the relaxation to the $S_{2,opt}$ state (E_{rel} = 286 kJmol^{-1}) that is ~58 kJmol^{-1} lower in energy (Figure S47). The bond lengths of this relaxed $S_{2,opt}$ state confirm a biradicalic character (Figure S48): one of the C-O bonds is strongly elongated (C-O = 1.318 Å), more than the other one (C-O = 1.309 Å), and the central C-C bond is strongly shortened by 0.127 Å relative to the ground state. From this $S_{2,opt}$ geometry, the molecule may relax by a transfer of the bulky hypersilyl group towards the oxygen atom forming compound **4**. A conical intersection (CI) between the S_2 and S_0 state was not calculated, because the molecule is too large for an CI search with ADC(2). Nevertheless, DFT calculations could locate a transition state at the S_0 potential energy surface (PES) (not shown) for the hypersilyl shift (see Figure S48), which has a relative PBEh-3c energy of 181 kJmol^{-1}. A single point energy calculation of this transition state at the ADC(2) level shows a vertical energy difference of 0.34 eV (~33 kJ/mol) between its S_0 and S_2 states, which is too much for a nearby conical intersection. Thus, we can only conclude that the CI must be at another point at the potential energy surface.

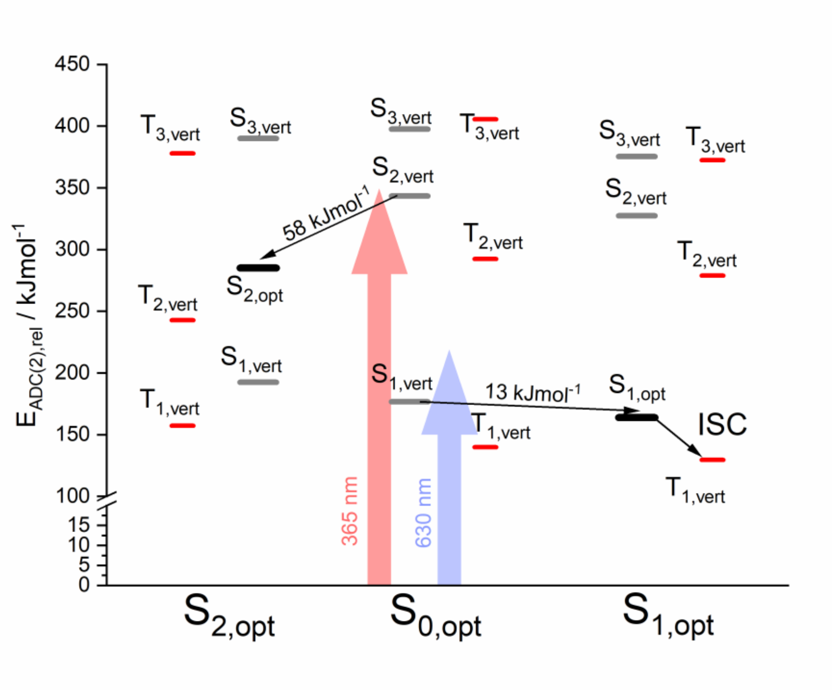

Figure S47. The vertical ADC(2) excitation energies of **3** for the MP2 optimized S_0 geometry ($S_{0,opt}$) and the SOS-ADC(2) optimized S_1 and S_2 states ($S_{1,opt}$ and $S_{2,opt}$, respectively). The singlet energies of the vertical states (grey) and optimized states (black) as well as the vertical triplet energies (red bars) are shown.

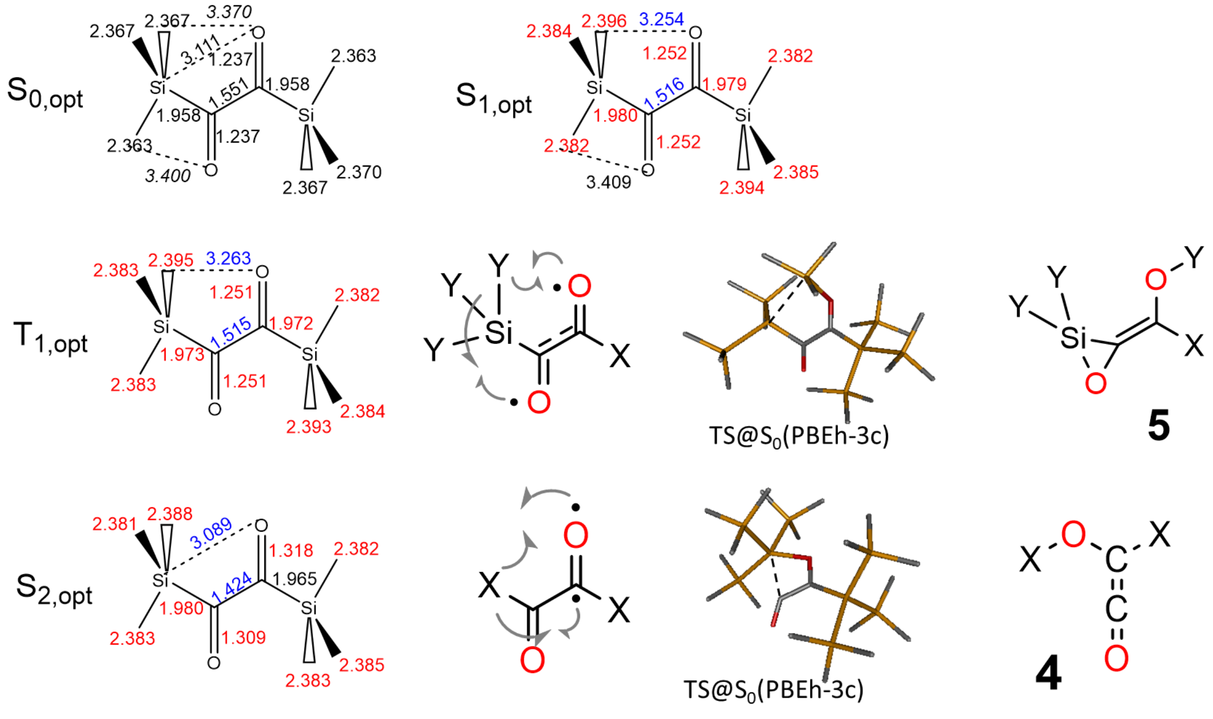

Figure S48. The bond lengths of the optimized MP2 S_0 geometry and SOS-ADC(2) S_1, S_2 and T_1 geometries of **3** (from top to bottom) with the Lewis structures, the PBEh-3c ground state transition state structures forming **5** (middle line) and **4** (bottom line), respectively.

After excitation of **3** with 630 nm light, the molecule reaches the S_1 state (177 kJmol^{-1}, ADC(2) vertical energy), which relaxes to $S_{1,opt}$ with 164 kJmol^{-1}. The relaxed S_1 state shows already a geometry with symmetrically elongated C=O bonds (Figure S48), which supports the mechanistic proposed in Figure 9 of the main manuscript. This S_1 state has n-π* character. After intersystem crossing (ISC), it relaxes to the triplet state (138 kJmol^{-1}, vertical ADC(2) energy), from where it can transfer one TMS group forming product **5**. The geometry of the relaxed

T_1 state (Figure S48) is similar to S_{1opt}, thus a fast ISC without geometry relaxation justifies an effective ISC. To understand the rearrangement pathway from **3** to **5**, PBEh-3c single point energies were calculated for the singlet and triplet excited states on the climbing-image nudged elastic band (CI-NEB) pathway, which connects the minima at the ground state PES with the transition state (TS) from both sides. The CI-NEB method calculates a linear reaction pathway between both minima as a first step, then computes the NEB energies and finally locates the transition state (TS). Please note that the optimized T_1 state is not shown at the NEB-CI curve in Figure S49.
After reaching the transition state of 28 kJmol^{-1} at the triplet state curve (green in Figure S49), the T_1 curve meets the ground state (black) curve, which is a strong indication of a conical intersection. Further, another possible region of conical intersection is present after the molecule has reached an intermediate structure (INT1). Thus, the relaxation to the ground state can occur via conical intersection (which was not located computationally) confirming the experimental findings of product **5** with a fast rate.

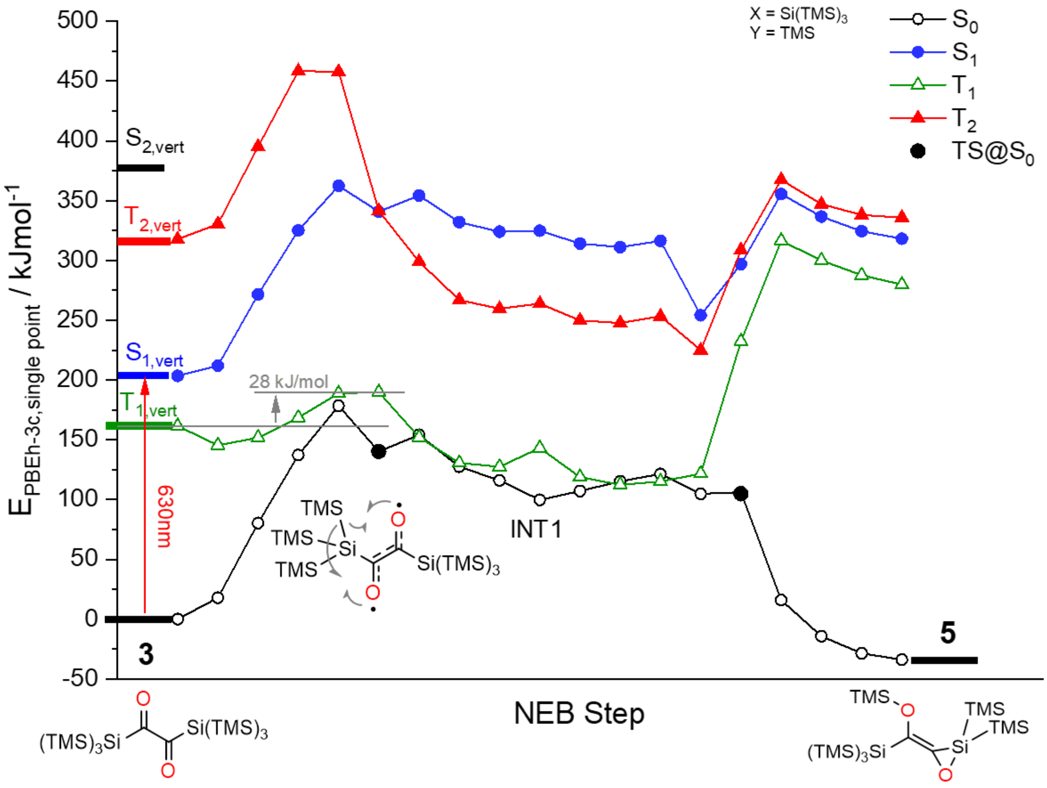

FigureS49. PBEh-3c ground state potential energy curve and vertical excited states for the CI-NEB steps of the reaction **3**→**5** *via* the intermediate (INT1). The relaxed transition states at the S_0 potential energy surface (black full circles) are marked. Note that the calculated NEB steps are printed equidistantly.

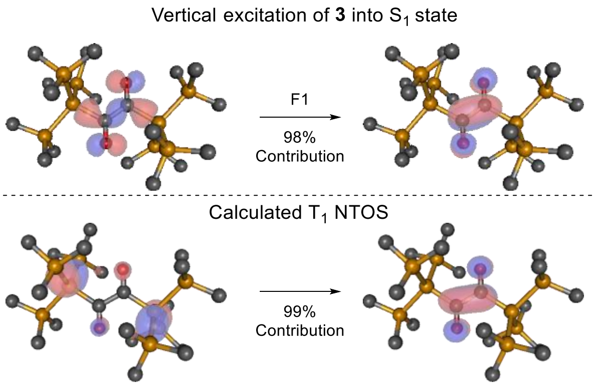

Figure S50. NTOs of the vertical S_1 singlet (top) and T_1 triplet (bottom) states of **3** at the Franck-Condon region ($S_{0,opt}$).

References

[1] A. B. Pangborn, M. A. Giardello, R. H. Grubbs, R. K. Rosen, F. J. Timmers, *Organometallics* 1996, *15*, 1518.
[2] *Purification of Laboratory Chemicals*, Elsevier, 2009.
[3] H. Gilman, C. L. Smith, *J. Organomet. Chem.* 1967, *8*, 245.
[4] S. Grimme, J. G. Brandenburg, C. Bannwarth, A. Hansen, *J. Chem. Phys.* 2015, *143*, 54107.
[5] F. Neese, *WIREs Comput Mol Sci* 2018, *8*, 33.
[6] A.-R. Allouche, *J. Comput. Chem.* 2011, *32*, 174.
[7] T. H. Dunning, *J. Chem. Phys.* 1989, *90*, 1007.
[8] a) A. Dreuw, M. Wormit, *WIREs Comput Mol Sci* 2015, *5*, 82; b) A. B. Trofimov, J. Schirmer, *J. Phys. B: At. Mol. Opt. Phys.* 1995, *28*, 2299; c) J. Schirmer, *Phys. Rev. A* 1982, *26*, 2395.
[9] a) I. Mayer, *Chem. Phys. Lett.* 2007, *437*, 284; b) R. L. Martin, *J. Chem. Phys.* 2003, *118*, 4775.
[10] M. Sapunar, T. Piteša, D. Davidović, N. Došlić, *J. Chem. Theory Comput.* 2019, *15*, 3461.
[11] F. Furche, R. Ahlrichs, C. Hättig, W. Klopper, M. Sierka, F. Weigend, *WIREs Comput Mol Sci* 2014, *4*, 91.
[12] a) V. Balzani, F. Bolletta, F. Scandola, *J. Am. Chem. Soc.* 1980, *102*, 2152; b) M. K. Kuimova, M. Hoffmann, M. U. Winters, M. Eng, M. Balaz, I. P. Clark, H. A. Collins, S. M. Tavender, C. J. Wilson, B. Albinsson et al., *Photochem. Photobiol. Sci.* 2007, *6*, 675.
[13] W. G. Herkstroeter, *J. Am. Chem. Soc.* 1975, *97*, 4161.
[14] W. G. Herkstroeter, P. B. Merkel, *J. Photochem.* 1981, *16*, 331.
[15] S. L. Murov, *Handbook of photochemistry*, Dekker, New York, 1993.
[16] S. P. McGlynn, T. Azumi, M. Kasha, *J. Chem. Phys.* 1964, *40*, 507.
[17] R. H. Clarke, R. M. Hochstrasser, *J. Mol. Spectr.* 1969, *32*, 309.
[18] D. F. Evans, *J. Chem. Soc.* 1957, *0*, 1351.
[19] J. Saltiel, G.-E. Khalil, K. Schanze, *Chem. Phys. Lett.* 1980, *70*, 233.
[20] a) Bruker APEX2 and SAINT, Bruker AXS Inc.: Madison, Wisconsin, USA, 2012.; b) R. H. Blessing, *Acta Crystallogr. A: Found. Adv.* 1995, *51 (Pt 1)*, 33.
[21] a) G. M. Sheldrick, Acta Crystallogr. A: Found. Adv. 1990, *46*, 467; b) G. M. Sheldrick, *Acta Crystallogr. A: Found. Adv.* 2008, *64*, 112; c) G. M. Sheldrick, *Acta Crystallogr. A: Found. Adv.* 2015, *71*, 3.
[22] O. V. Dolomanov, L. J. Bourhis, R. J. Gildea, J. A. K. Howard, H. Puschmann, *J. Appl. Crystallogr.* 2009, *42*, 339.
[23] a) A. L. Spek, *J. Appl. Crystallogr.* 2003, *36*, 7; b) A. L. Spek, *Acta Crystallogr. D: Bio. cryst.* 2009, *65*, 148.
[24] P. Müller, R. Herbst-Irmer, A. L. Spek, T. R. Schneider, Sawaya, M. R., Crystal Structure Refinement: A Crystallographer's Guide to SHELXL Oxford University Press: 2006, p 232.
[25] T. K. Hy, *Chem. Phys. Lett.* 1978, *57*, 64.
[26] J. W. Sidman, D. S. McClure *J. Am. Chem. Soc.* 1955, 77, 24, 6461.
